# Supplementary material for: Early high rates and disparity in the evolution of ichthyosaurs
Source: Commun Biol. 2020 Feb 13;3:68. doi: 10.1038/s42003-020-0779-6 (PMC7018711; doi:10.1038/s42003-020-0779-6)
Supplement: Supplementary file 1 — Reporting Summary [file 42003_2020_779_MOESM1_ESM.pdf]

nature research

Corresponding author(s): Benjamin Moon

Last updated by author(s): Dec 19, 2019

Reporting Summary

Nature Research wishes to improve the reproducibility of the work that we publish. This form provides structure for consistency and transparency in reporting. For further information on Nature Research policies, see Authors & Referees and the Editorial Policy Checklist.

Statistics

For all statistical analyses, confirm that the following items are present in the figure legend, table legend, main text, or Methods section.

n/a	Confirmed	
☒	☐	The exact sample size (n) for each experimental group/condition, given as a discrete number and unit of measurement
☐	☒	A statement on whether measurements were taken from distinct samples or whether the same sample was measured repeatedly
☐	☒	The statistical test(s) used AND whether they are one- or two-sided<br>*Only common tests should be described solely by name; describe more complex techniques in the Methods section.*
☒	☐	A description of all covariates tested
☐	☒	A description of any assumptions or corrections, such as tests of normality and adjustment for multiple comparisons
☐	☒	A full description of the statistical parameters including central tendency (e.g. means) or other basic estimates (e.g. regression coefficient) AND variation (e.g. standard deviation) or associated estimates of uncertainty (e.g. confidence intervals)
☐	☒	For null hypothesis testing, the test statistic (e.g. F, t, r) with confidence intervals, effect sizes, degrees of freedom and P value noted<br>*Give P values as exact values whenever suitable.*
☐	☒	For Bayesian analysis, information on the choice of priors and Markov chain Monte Carlo settings
☒	☐	For hierarchical and complex designs, identification of the appropriate level for tests and full reporting of outcomes
☒	☐	Estimates of effect sizes (e.g. Cohen's d, Pearson's r), indicating how they were calculated

Our web collection on statistics for biologists contains articles on many of the points above.

Software and code

Policy information about availability of computer code

Data collection	No software was used
Data analysis	Custom code in R was used to run the disparity, tree time-scaling and discrete rates analyses, incorporating open source packages. BayesTraits (http://www.evolution.rdg.ac.uk/BayesTraitsV2.0.2.html) was used for analysis of continuous rates analyses. All code has been included in the supplementary information and is hosted at on Zenodo and GitHub at DOI 10.5281/zenodo.3584386.

For manuscripts utilizing custom algorithms or software that are central to the research but not yet described in published literature, software must be made available to editors/reviewers. We strongly encourage code deposition in a community repository (e.g. GitHub). See the Nature Research guidelines for submitting code & software for further information.

Data

Policy information about availability of data

All manuscripts must include a data availability statement. This statement should provide the following information, where applicable:

- Accession codes, unique identifiers, or web links for publicly available datasets
- A list of figures that have associated raw data
- A description of any restrictions on data availability

Cladistic is taken from Moon (2018) and is available with that paper. Phenomic data is included in the supplementary information and hosted in Zenodo and GitHub DOI 10.5281/zenodo.3584386.

Field-specific reporting

Please select the one below that is the best fit for your research. If you are not sure, read the appropriate sections before making your selection.

☐ Life sciences ☐ Behavioural & social sciences ☒ Ecological, evolutionary & environmental sciences

For a reference copy of the document with all sections, see nature.com/documents/nr-reporting-summary-flat.pdf

Ecological, evolutionary & environmental sciences study design

All studies must disclose on these points even when the disclosure is negative.

Study description	We study the evolution of ichthyosaurs through the use of discrete and continuous character evolution. Using a combination of computation disparity and comparative phylogenetics we aim to study the trends through 160 Ma of evolution. Our methods include disparity analysis through distance matrices and variation was tested using 500 bootstrap replicates and the 95% confidence interval for each time bin used. Morphospace occupation with visualied using principal coordinate ordination, with pariwise PERMANOVA tests showing separation between groupings. Discrete character evolution with maximum likelihood optimisation on a sample of trees taken from the analysis of Moon (2018). Continuous character evolution using the variable rates model in BayesTraits on the same sample sample of trees. All these procedures are described in the Methods section of the typescript.
Research sample	114 ichthyosauriform taxa from the phylogenetic analysis of Moon (2018) that were coded for 287 characters; 67 taxa for which accurate skull length data could be collected from personal observation and the literature.
Sampling strategy	All 114 ichthyosauriform taxa from the study of Moon (2018) were included in the initial disparity analyses; some taxa were excluded based on incompleteness and lack of comparable data. 104 ichthyosauriform taxa that were included in the resultant phylogenetic trees of Moon (2018) were included in the rates analyses. 67 taxa for which skull length data could be collected were included in the continuous rates analysis.
Data collection	Occurrence data were collected by BCM using published records where possible to the nearest ammonite or conodont biozone. Bio- and lithostratigraphic data were converted to chronostratigraphic dates using Geologic Time Scale 2012 (Gradstein et al. 2012). Continuous skull length measurements were take from the primary literature or measurements by BCM using calipers or measuring tape (Supplementary Information).
Timing and spatial scale	The data were collected during a 2 year period by BCM visiting museums in the UK and Germany.
Data exclusions	Data were excluded only following Moon (2018) where taxa were not included within the resultant phylogenetic trees.
Reproducibility	A detailed account of the measurements is included in the Supplementary information.
Randomization	Taxa were allocated to groups based on occurrence dates and the Geologic Time Scale. Bootstrap replicates (with replacement) for each bin were used to assess variation in the disparity analyses.
Blinding	N/A

Did the study involve field work? ☐ Yes ☒ No

Reporting for specific materials, systems and methods

We require information from authors about some types of materials, experimental systems and methods used in many studies. Here, indicate whether each material, system or method listed is relevant to your study. If you are not sure if a list item applies to your research, read the appropriate section before selecting a response.

Materials & experimental systems

n/a	Involved in the study	
☒	☐	Antibodies
☒	☐	Eukaryotic cell lines
☐	☒	Palaeontology
☒	☐	Animals and other organisms
☒	☐	Human research participants
☒	☐	Clinical data

Methods

n/a	Involved in the study	
☒	☐	ChIP-seq
☒	☐	Flow cytometry
☒	☐	MRI-based neuroimaging

Palaeontology

Specimen provenance	All specimens come from internationally recognised institutions that were visited by BCM personally with the permission of the curators, or from published scientific literature.

Specimen deposition

Specimen data were collected from the literature or personal observation. Specimens are deposited in BRSMG Bristol City Museum and Art Gallery, Bristol UK; CAMSM Sedgwick Museum of Earth Sciences, Cambridge UK; GLAHM Hunterian Museum < Glasgow UK; NHMUK Natural history Museum, London UK; PETMG Peterborough Museum and Art Gallery, Peterborough UK; SMNS Saatliches Museum für Naturkunde, Stuttgart, Germany.

Dating methods

Specimens were assigned to different time bins based on the geological formations and biozone horizons in which they were found. Absolute dates were calculated using the Geologic Time Scale 2012 (Gradstein et al. 2012).

☒ Tick this box to confirm that the raw and calibrated dates are available in the paper or in Supplementary Information.
