# Supplementary material for: Early high rates and disparity in the evolution of ichthyosaurs
Source: Commun Biol. 2020 Feb 13;3:68. doi: 10.1038/s42003-020-0779-6 (PMC7018711; doi:10.1038/s42003-020-0779-6)
Supplement: Supplementary file 3 — Supplementary Information [file 42003_2020_779_MOESM3_ESM.pdf]

Supplementary material for ‘Early high rates and disparity in the evolution of ichthyosaurs’

Benjamin C. Moon Thomas L. Stubbs

18 December 2019 1.0.1380

Contents

Supplementary figures **1**

Supplementary tables **78**

Supplementary code **86**

Supplementary methods **86**

Supplementary results **87**

Supplementary figures

1 Per-bin discrete skeletal disparity of Ichthyosauriformes though the Mesozoic . . 1
2 Per-bin rarefaction curves for each disparity-time curve shown in Supplementary Figure 1 . . . 3
3 Cumulative variance described by axes of the ordinated data and the correlation of these axes with the original data set . . . 20
4 Per-bin discrete skeletal disparity of Ichthyosauriformes through the Mesozoic from ordinated data . . . 20
5 Per-bin rarefaction curves for each disparity-time curve shown in Supplementary Figure 4 . . . 24
6 Morphospace occupation of Ichthyosauriformes through the Mesozoic . . . 73
7 Rates of discrete skeletal character evolution in Ichthyosauriformes . . . 76
8 Rates of skull size evolution in Ichthyosauriformes . . . 76

Supplementary figure 1. (following page) **Per-bin discrete skeletal disparity of Ichthyosauriformes though the Mesozoic.** Pairwise and weighted pairwise dissimilarity measured from raw Euclidean (RAW), generalised Euclidean (GED), Gower (GOW), and maximum observed rescaled (MAX) distances between taxa in the cladistic dataset of Moon [1] binned into epochs and equal 10-million-year bins. Also, pairwise number of comparable characters between taxa (CHAR) indicating the variation in completeness and comparability in each bin. Mean values and 95% confidence intervals are shown from 500 bootstrap replicates.

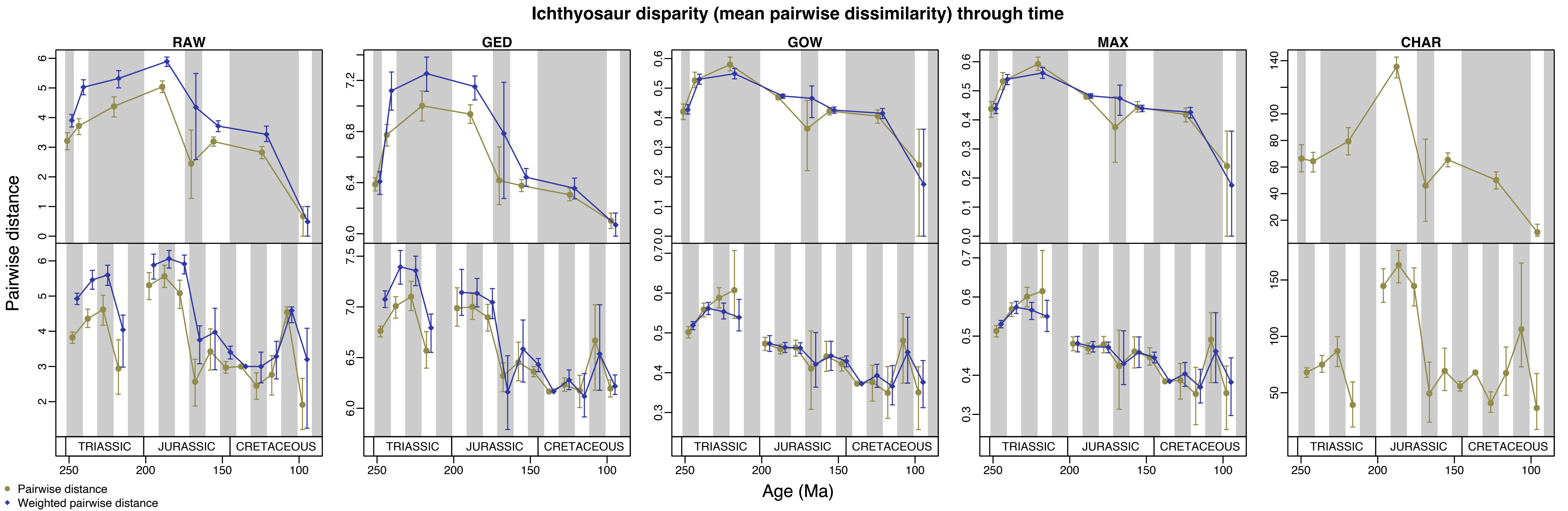

Ichthyosaur disparity (mean pairwise dissimilarity) through time
RAW
GED
GOW
MAX
CHAR
Pairwise distance
TRIASSIC
JURASSIC
CRETACEOUS
250
200
150
100
Age (Ma)
Pairwise distance
Weighted pairwise distance

Supplementary figure 2. (following pages) **Per-bin rarefaction curves for each disparity-time curve shown in Supplementary Figure 1.** Disparity for each pin is sequentiall rarefied on taxon occurrnece. Error polygon gives 95% confidence interval from 500 replicates.

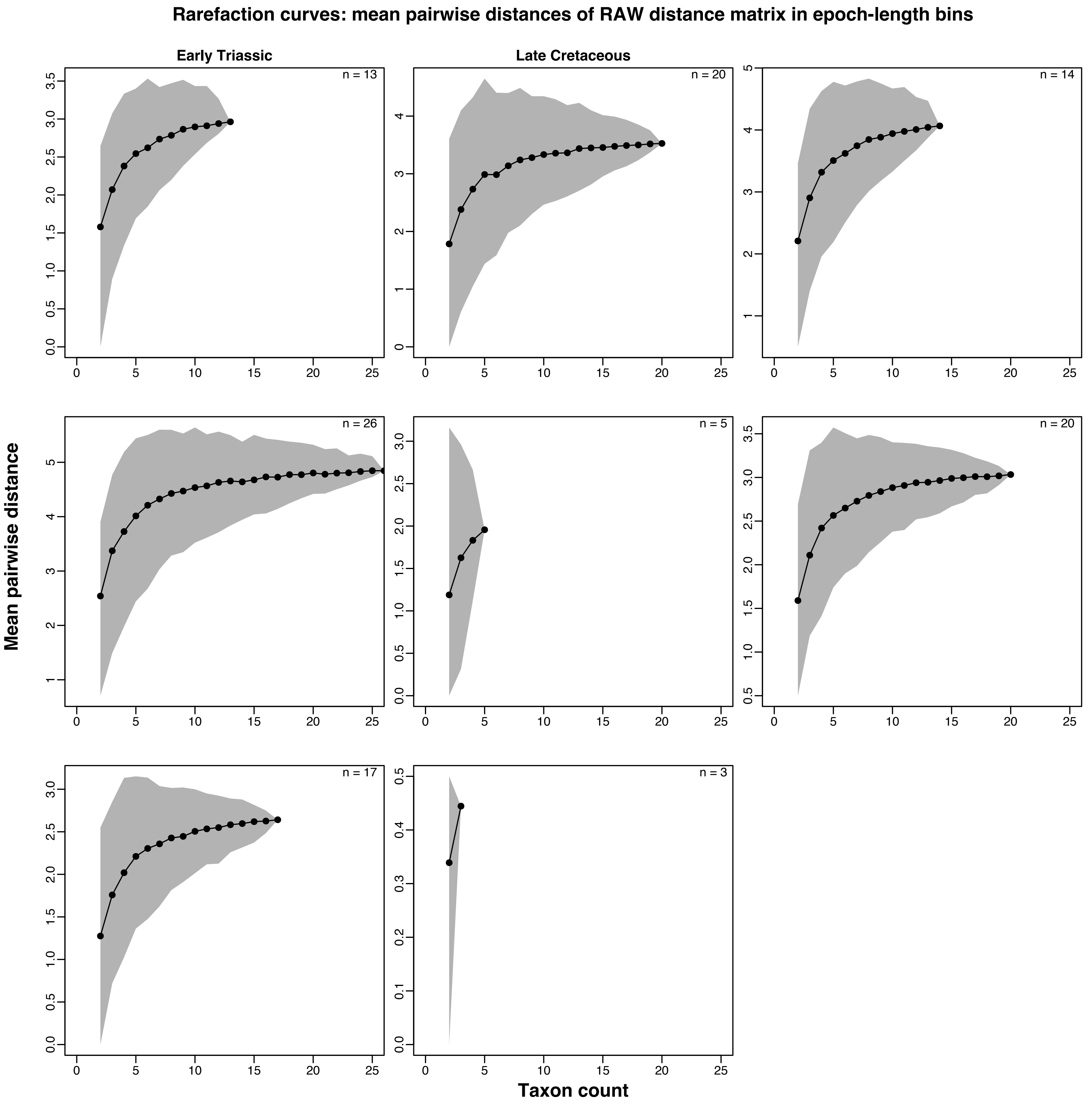

Rarefaction curves: mean pairwise distances of RAW distance matrix in epoch-length bins
Early Triassic
Late Cretaceous
n = 13
n = 20
n = 14
n = 26
n = 5
n = 20
n = 17
n = 3
Mean pairwise distance
Taxon count

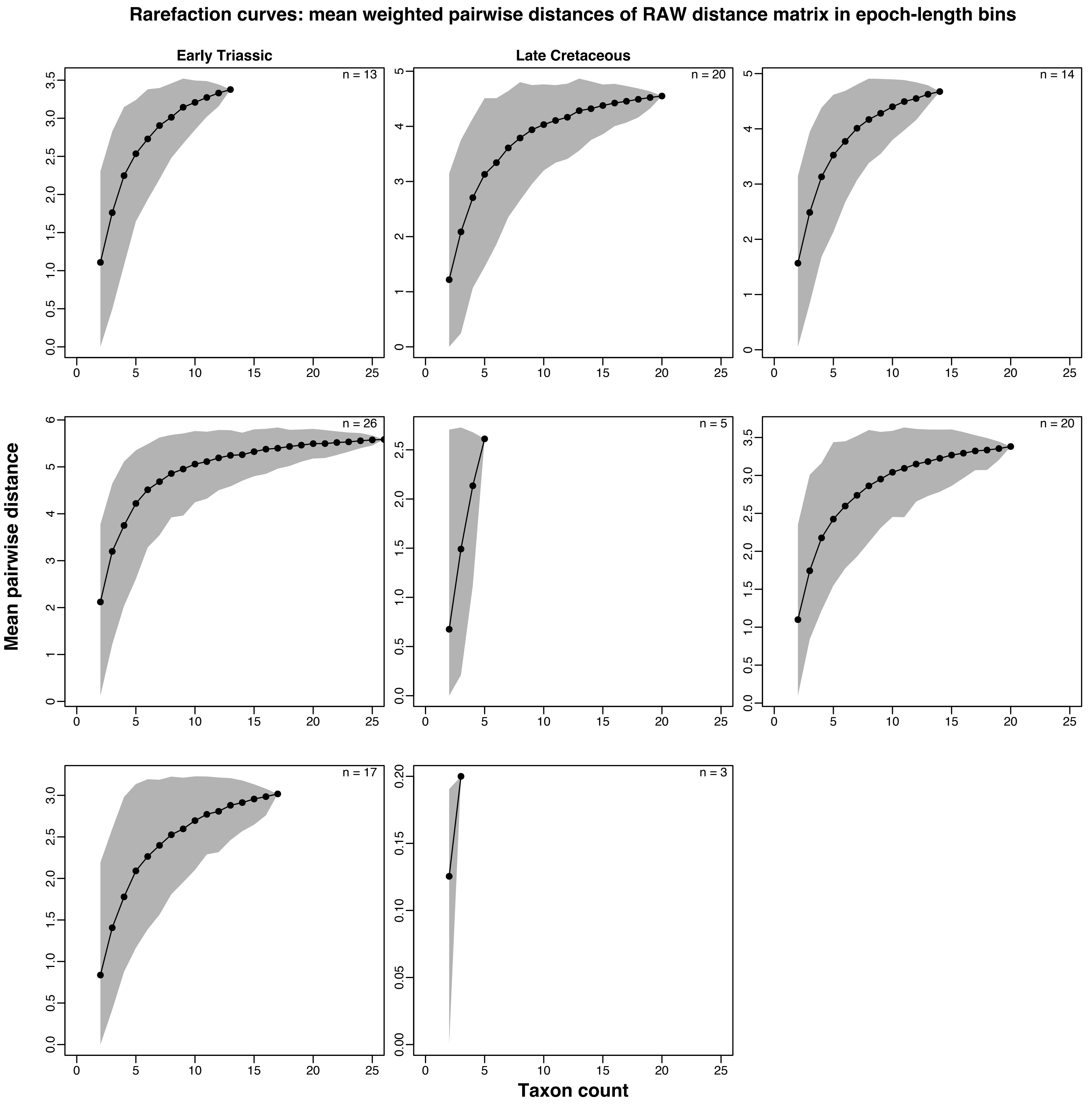

Rarefaction curves: mean weighted pairwise distances of RAW distance matrix in epoch-length bins
Early Triassic
Late Cretaceous
n = 13
n = 20
n = 14
n = 26
n = 5
n = 20
n = 17
n = 3
Mean pairwise distance
Taxon count

Rarefaction curves: mean pairwise distances of RAW distance matrix in 10 Ma bins

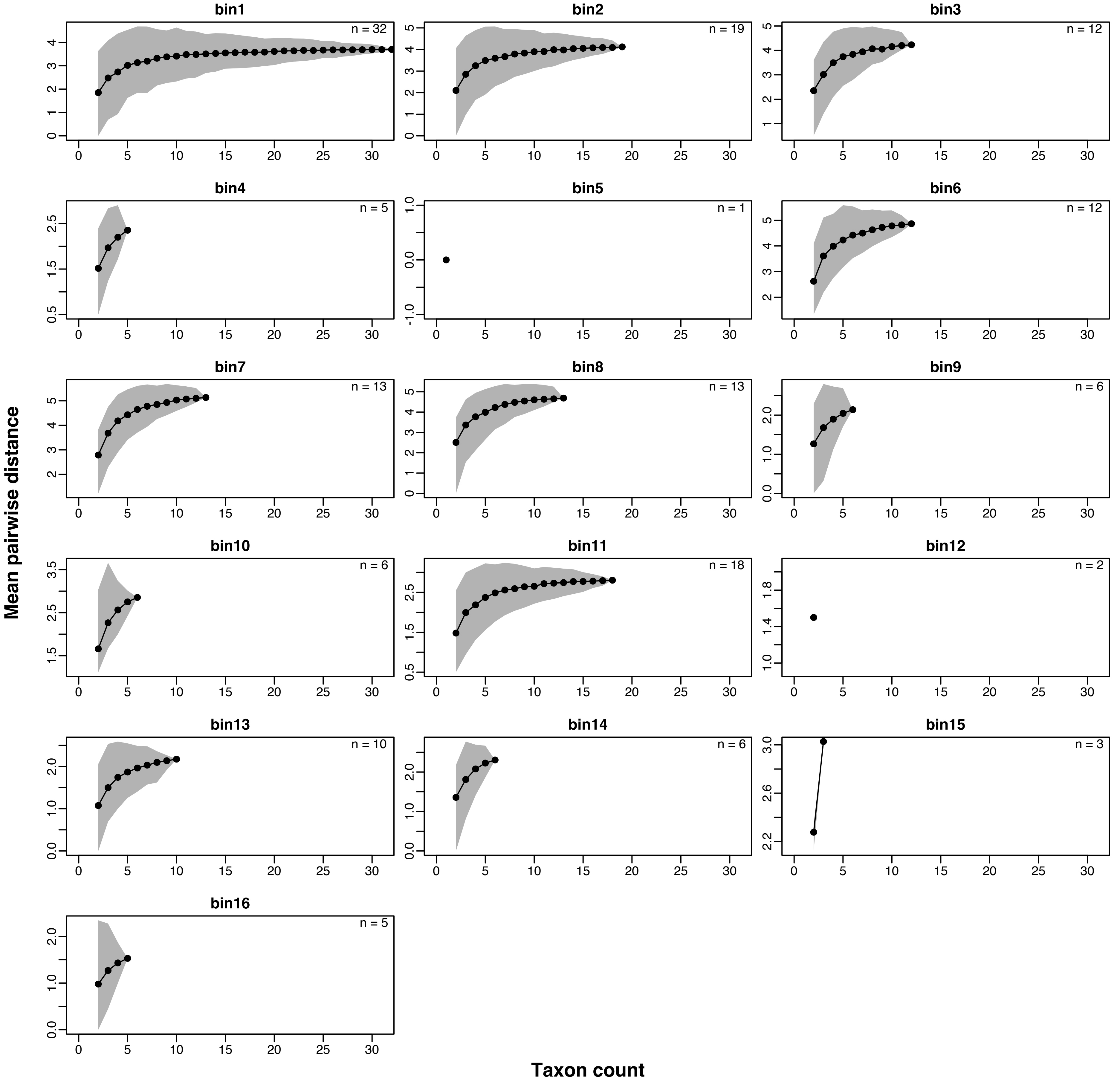

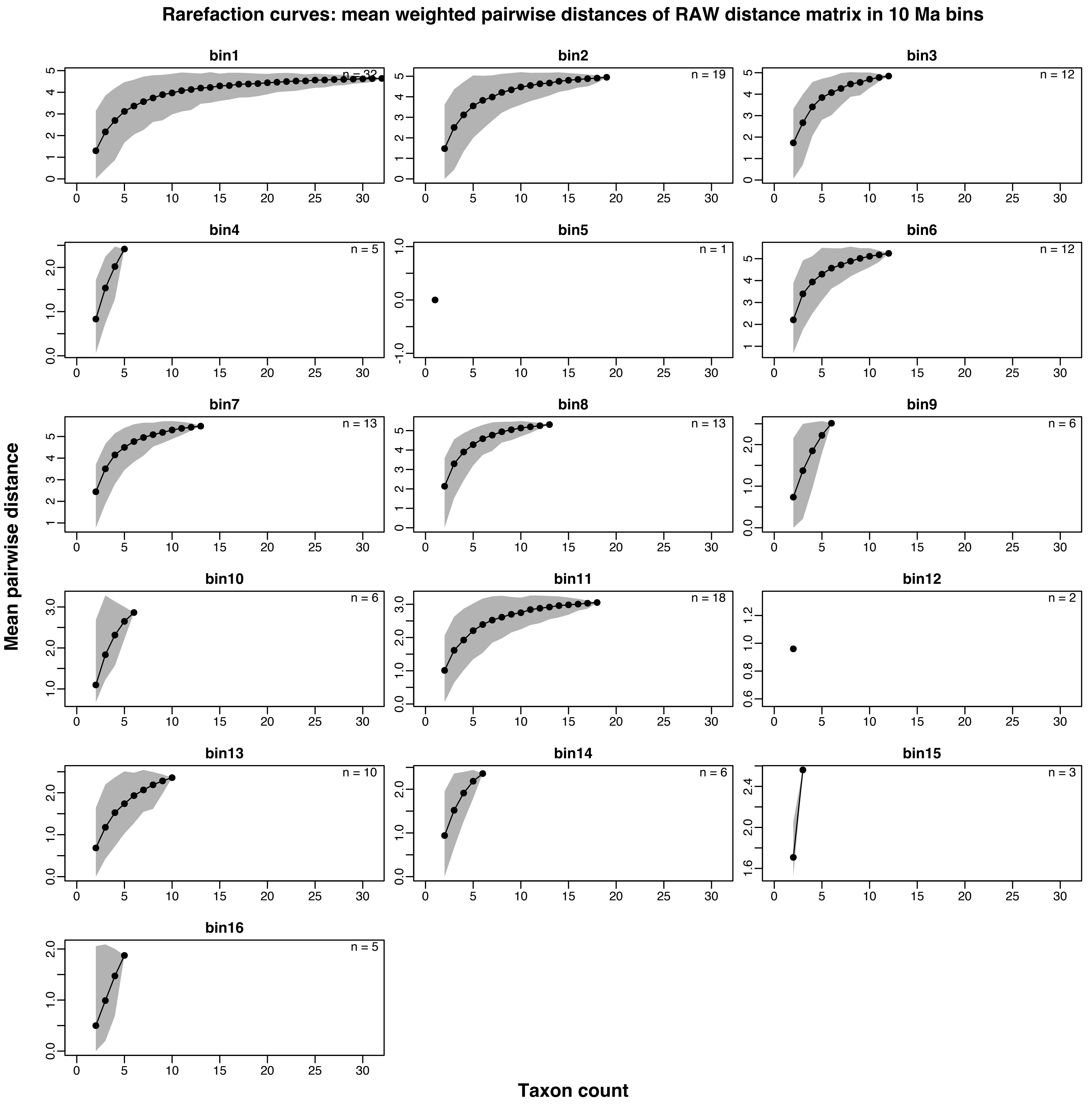

Rarefaction curves: mean weighted pairwise distances of RAW distance matrix in 10 Ma bins
bin1
n = 32
bin2
n = 19
bin3
n = 12
bin4
n = 5
bin5
n = 1
bin6
n = 12
bin7
n = 13
bin8
n = 13
bin9
n = 6
bin10
n = 6
bin11
n = 18
bin12
n = 2
bin13
n = 10
bin14
n = 6
bin15
n = 3
bin16
n = 5
Mean pairwise distance
Taxon count

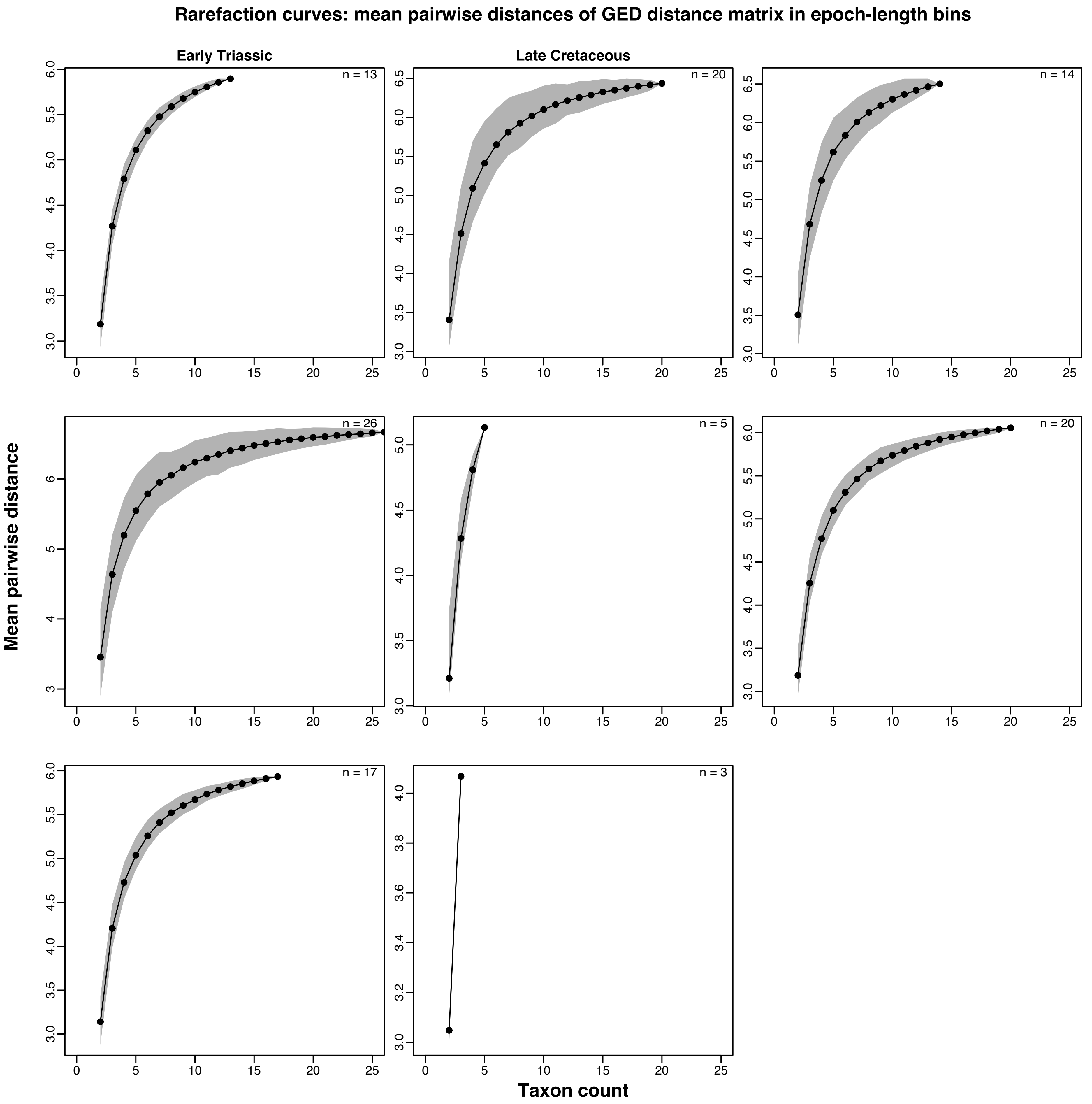

Rarefaction curves: mean pairwise distances of GED distance matrix in epoch-length bins
Early Triassic
Late Cretaceous
n = 13
n = 20
n = 14
n = 26
n = 5
n = 20
n = 17
n = 3
Mean pairwise distance
Taxon count

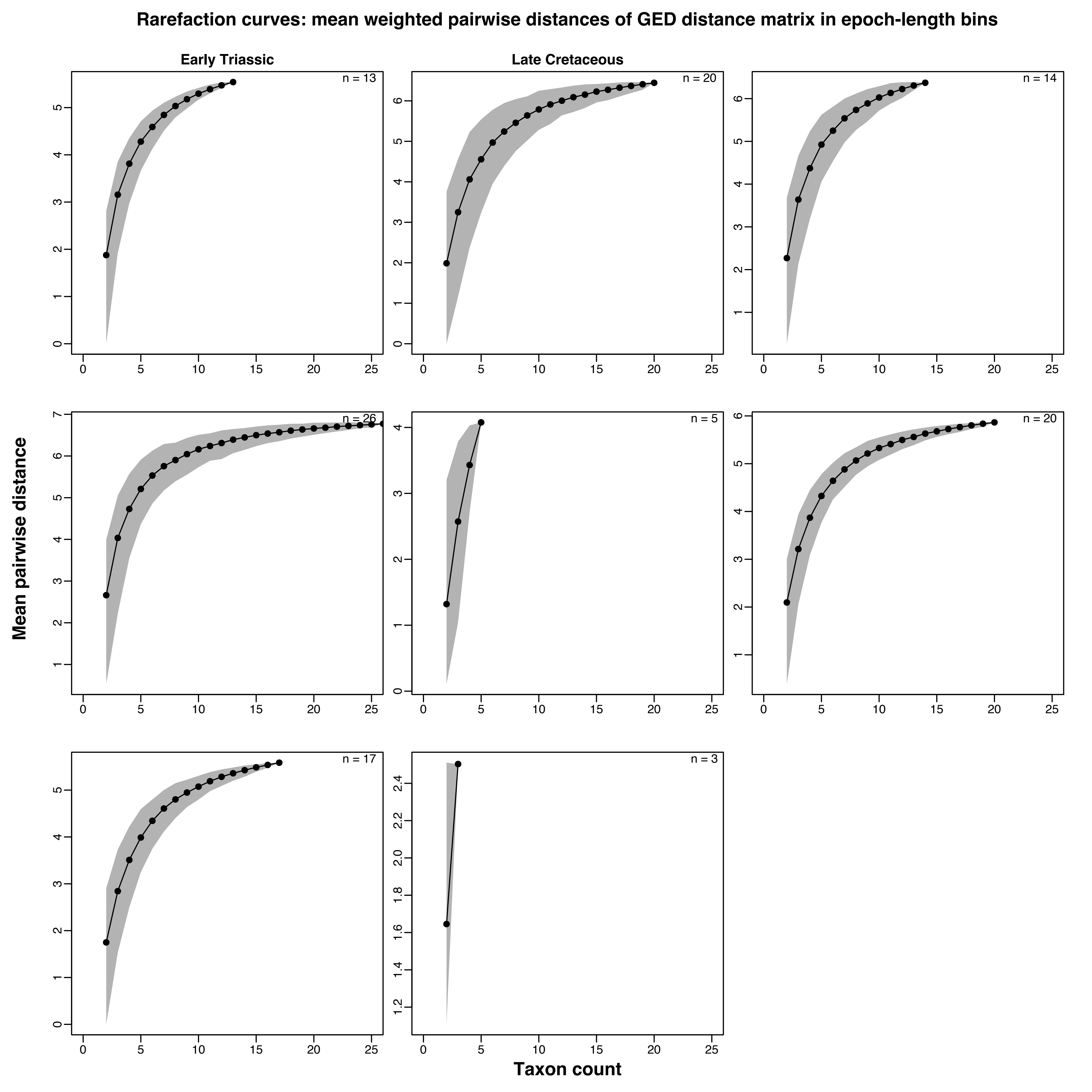
Rarefaction curves: mean weighted pairwise distances of GED distance matrix in epoch-length bins
Early Triassic
Late Cretaceous
n = 13
n = 20
n = 14
n = 26
n = 5
n = 20
n = 17
n = 3
Mean pairwise distance
Taxon count

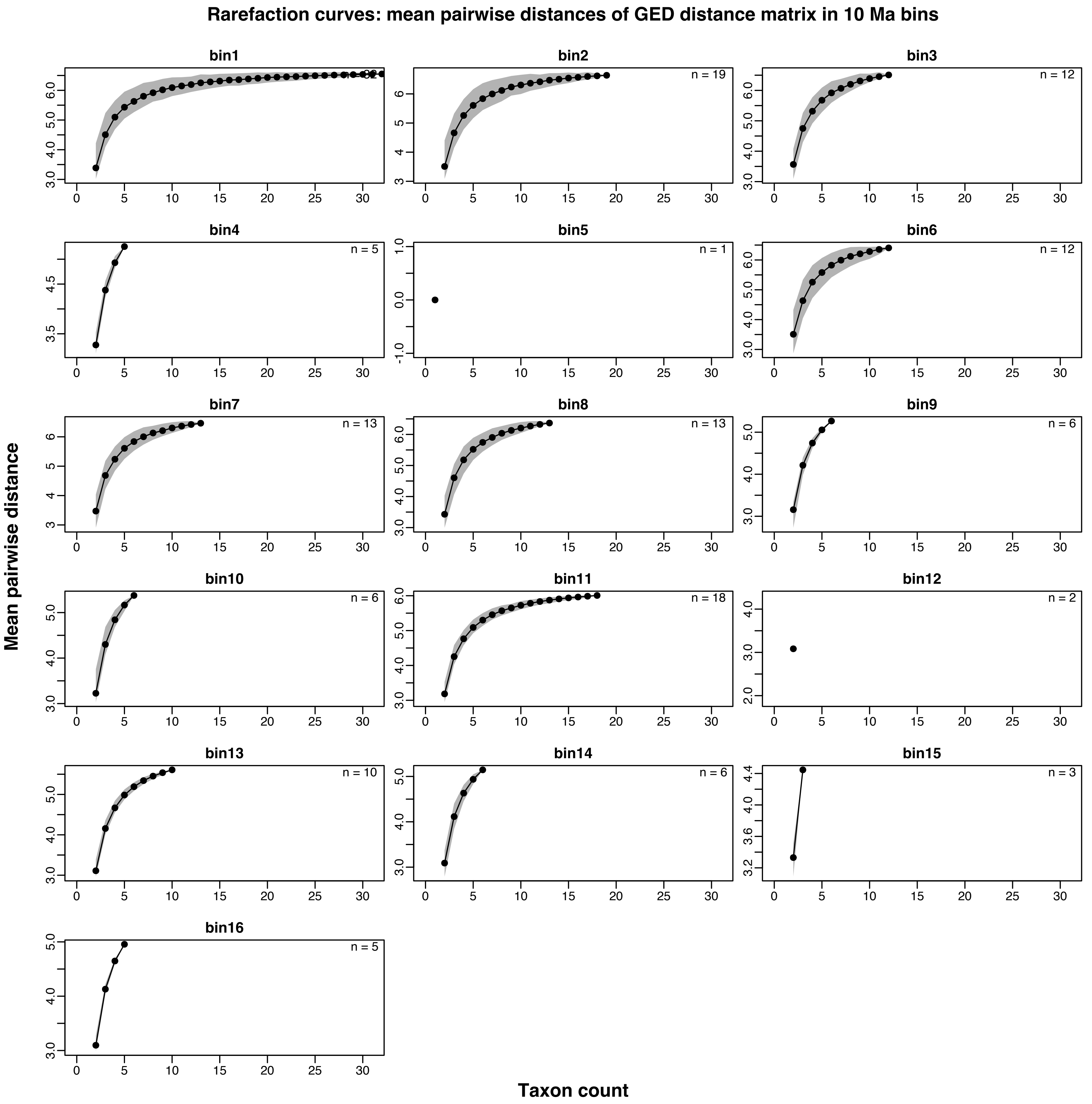

Rarefaction curves: mean pairwise distances of GED distance matrix in 10 Ma bins
bin1
n = 32
bin2
n = 19
bin3
n = 12
bin4
n = 5
bin5
n = 1
bin6
n = 12
bin7
n = 13
bin8
n = 13
bin9
n = 6
bin10
n = 6
bin11
n = 18
bin12
n = 2
bin13
n = 10
bin14
n = 6
bin15
n = 3
bin16
n = 5
Mean pairwise distance
Taxon count

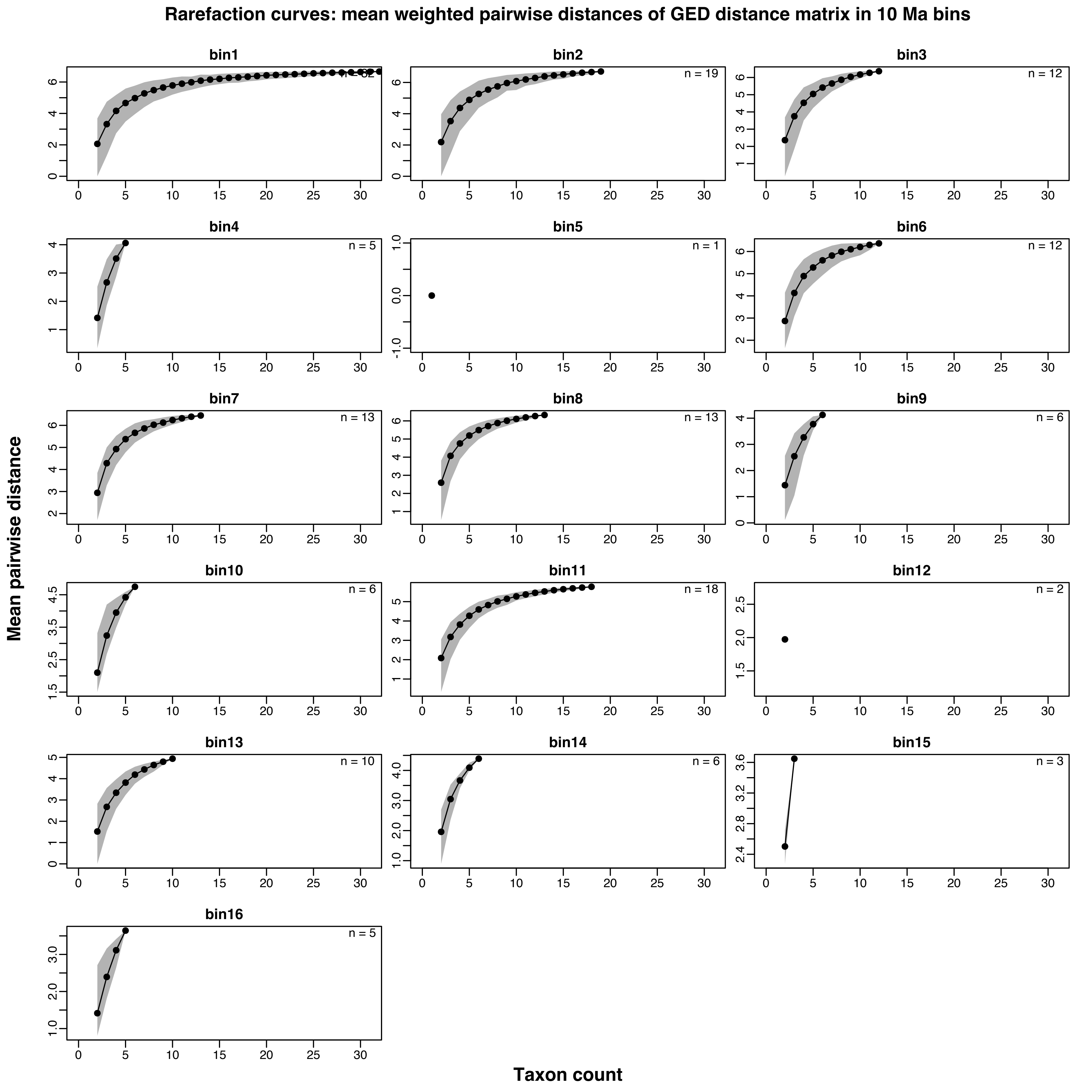
Rarefaction curves: mean weighted pairwise distances of GED distance matrix in 10 Ma bins
bin1
bin2
n = 19
bin3
n = 12
bin4
n = 5
bin5
n = 1
bin6
n = 12
bin7
n = 13
bin8
n = 13
bin9
n = 6
bin10
n = 6
bin11
n = 18
bin12
n = 2
bin13
n = 10
bin14
n = 6
bin15
n = 3
bin16
n = 5
Mean pairwise distance
Taxon count

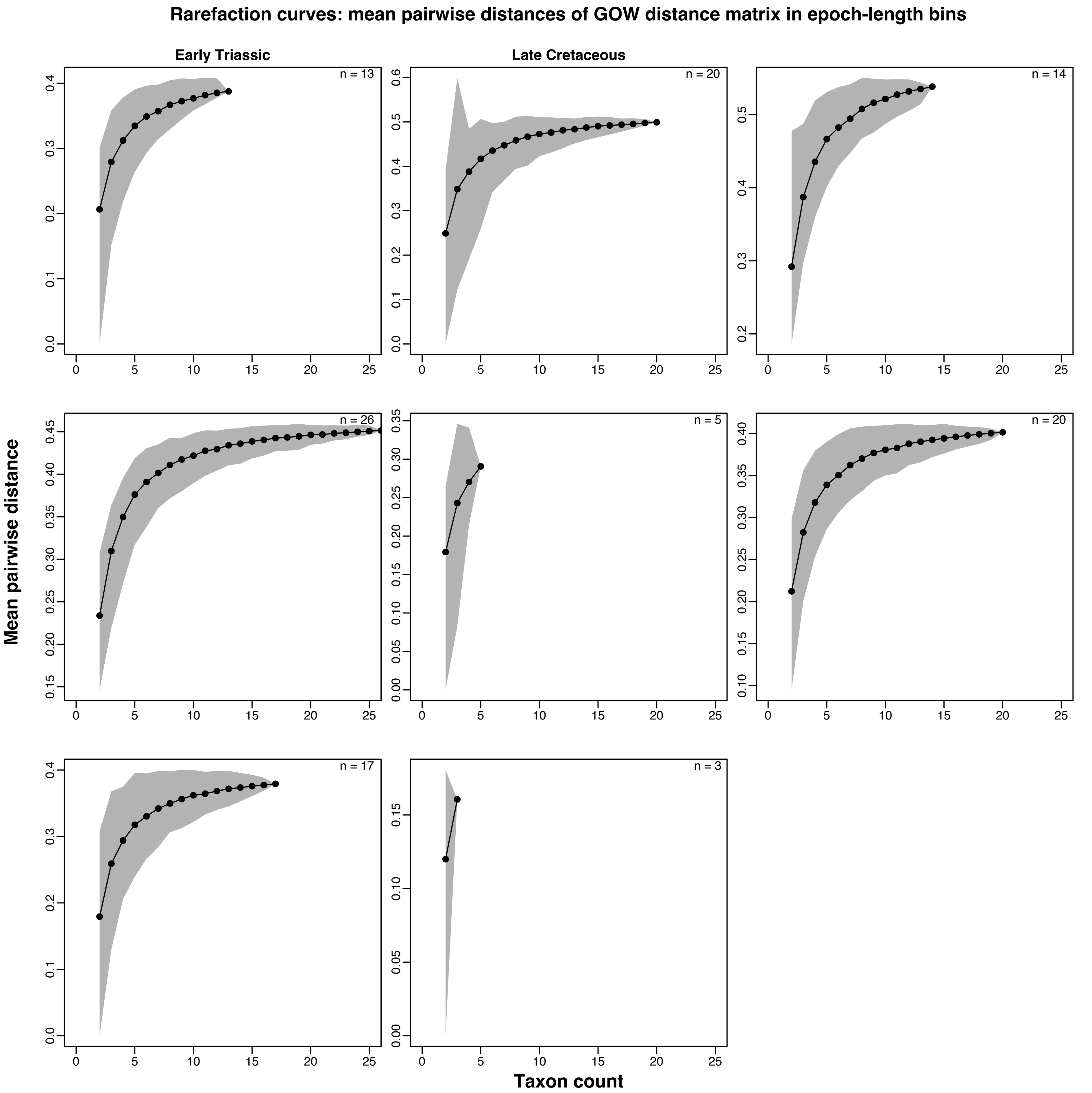
Rarefaction curves: mean pairwise distances of GOW distance matrix in epoch-length bins
Early Triassic
Late Cretaceous
n = 13
n = 20
n = 14
n = 26
n = 5
n = 20
n = 17
n = 3
Mean pairwise distance
Taxon count

Rarefaction curves: mean weighted pairwise distances of GOW distance matrix in epoch-length bins

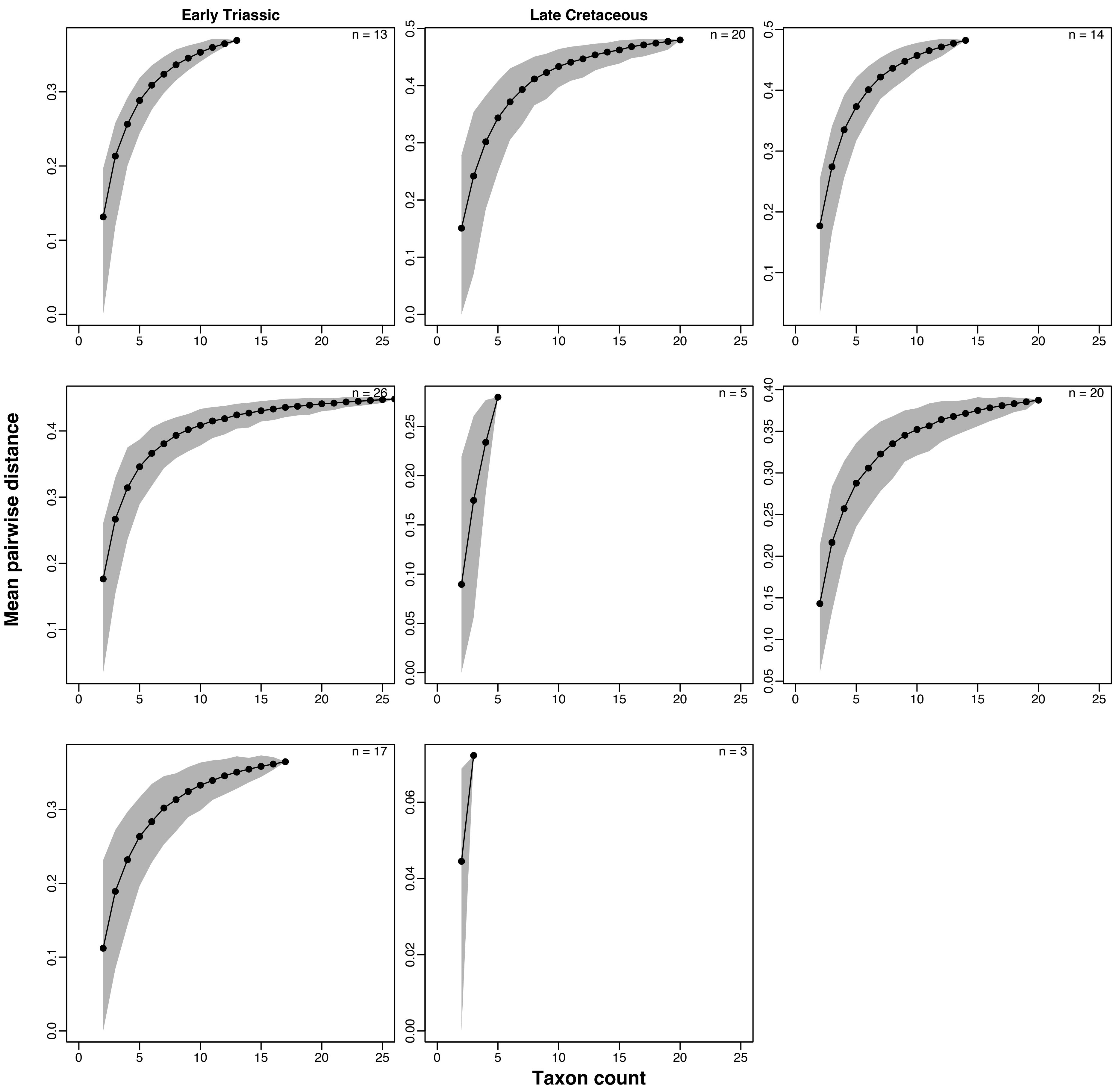

Rarefaction curves: mean pairwise distances of GOW distance matrix in 10 Ma bins

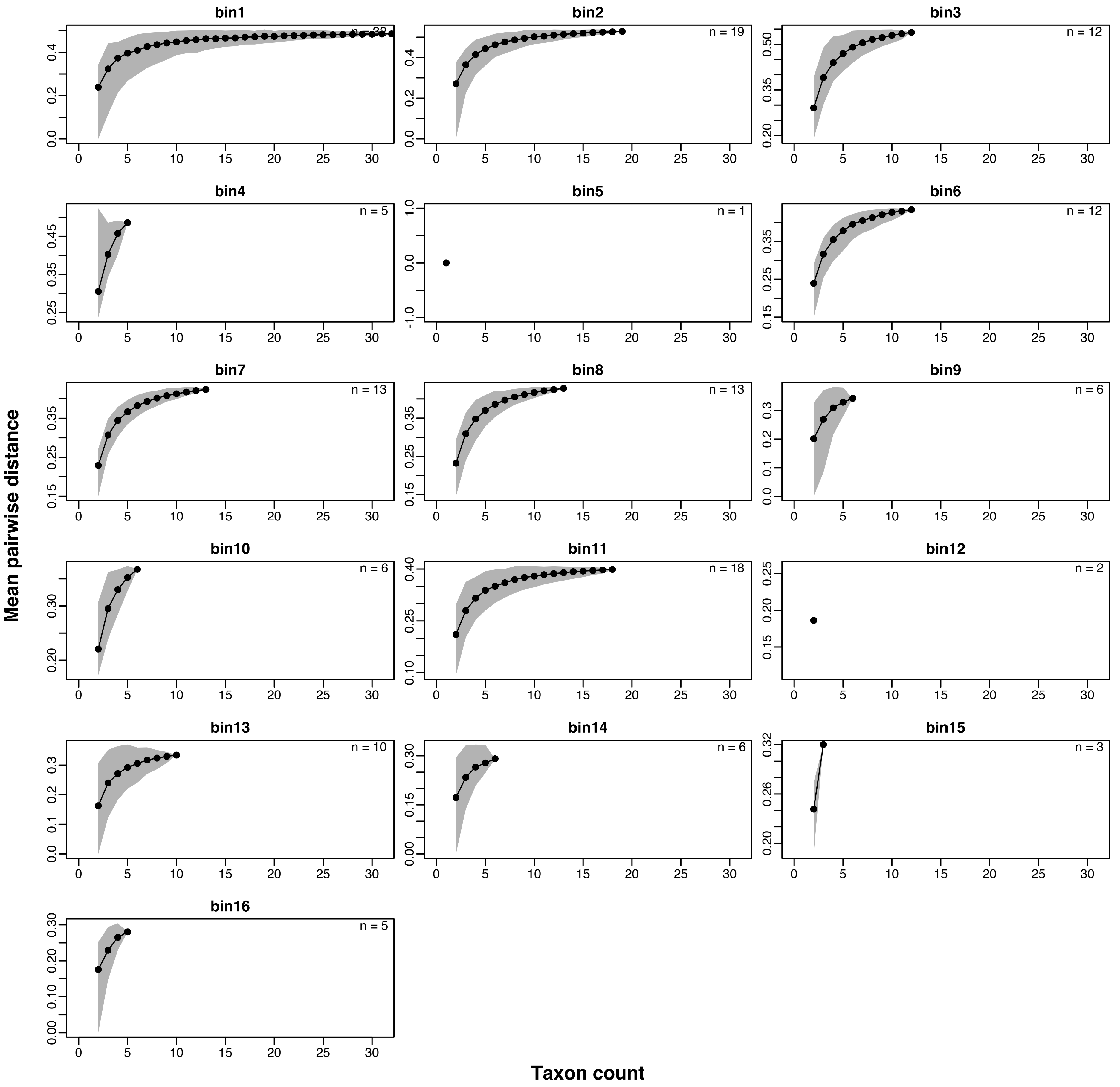

Rarefaction curves: mean weighted pairwise distances of GOW distance matrix in 10 Ma bins

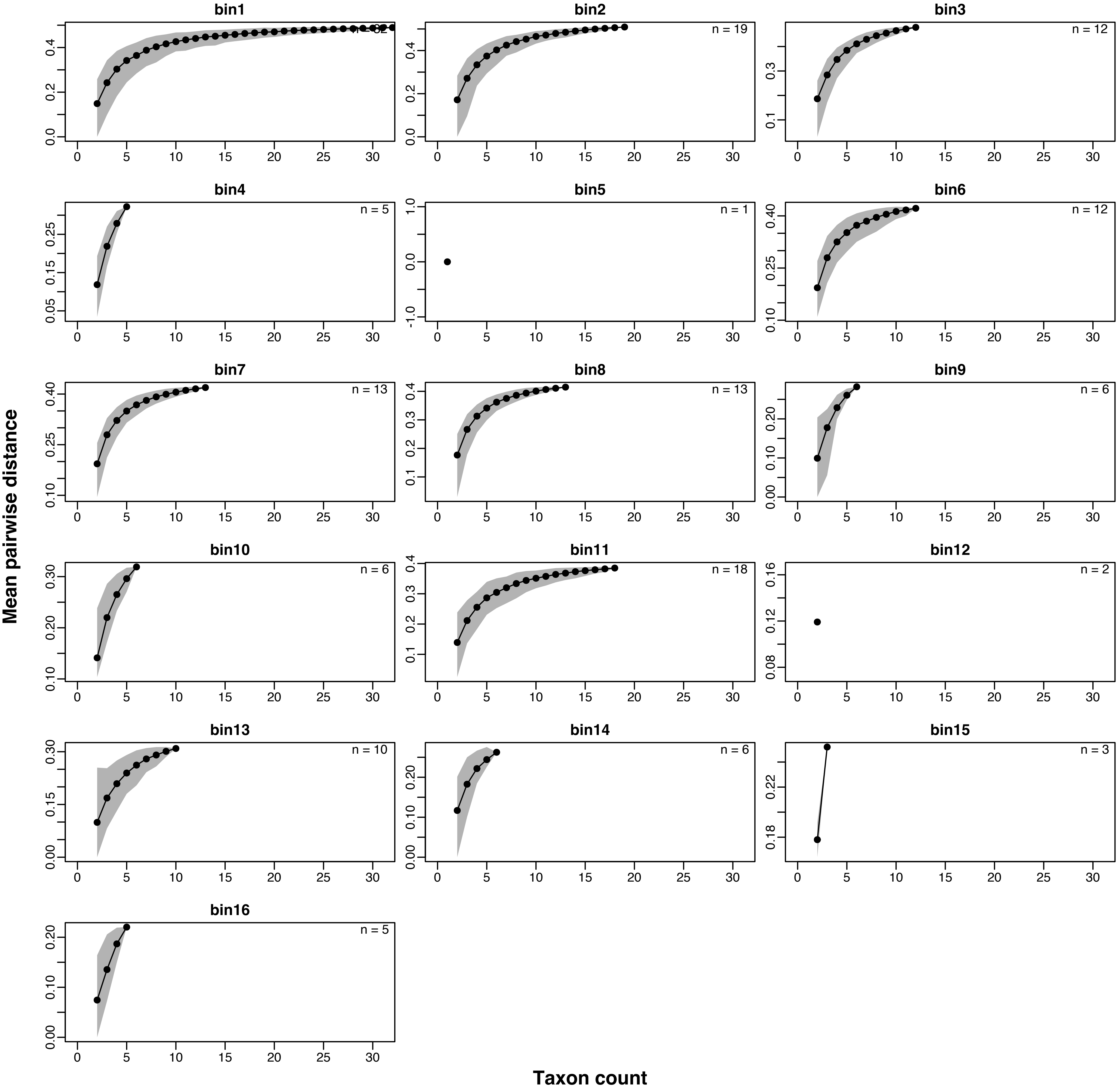

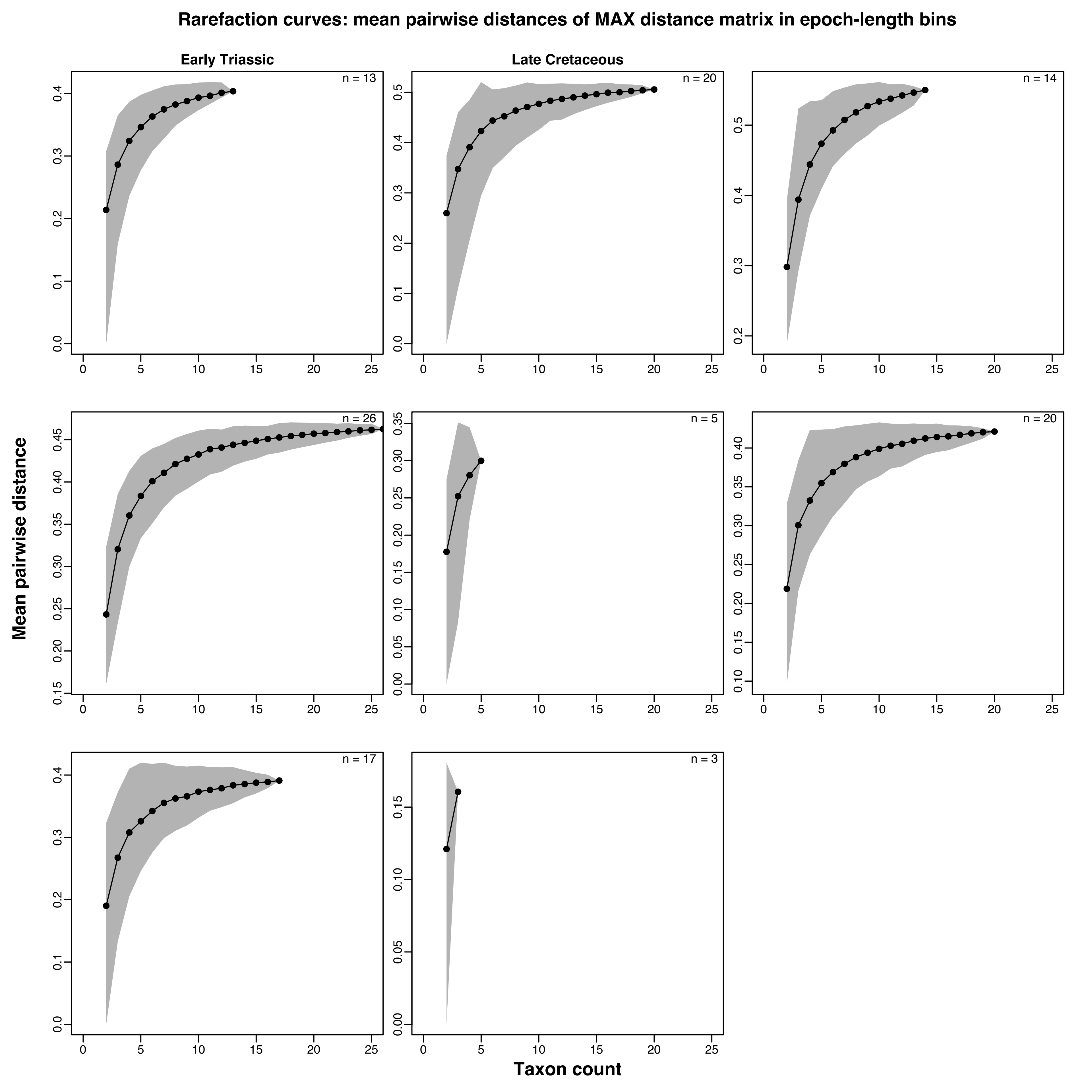
Rarefaction curves: mean pairwise distances of MAX distance matrix in epoch-length bins
Early Triassic
Late Cretaceous
n = 13
n = 20
n = 14
n = 26
n = 5
n = 20
n = 17
n = 3
Mean pairwise distance
Taxon count

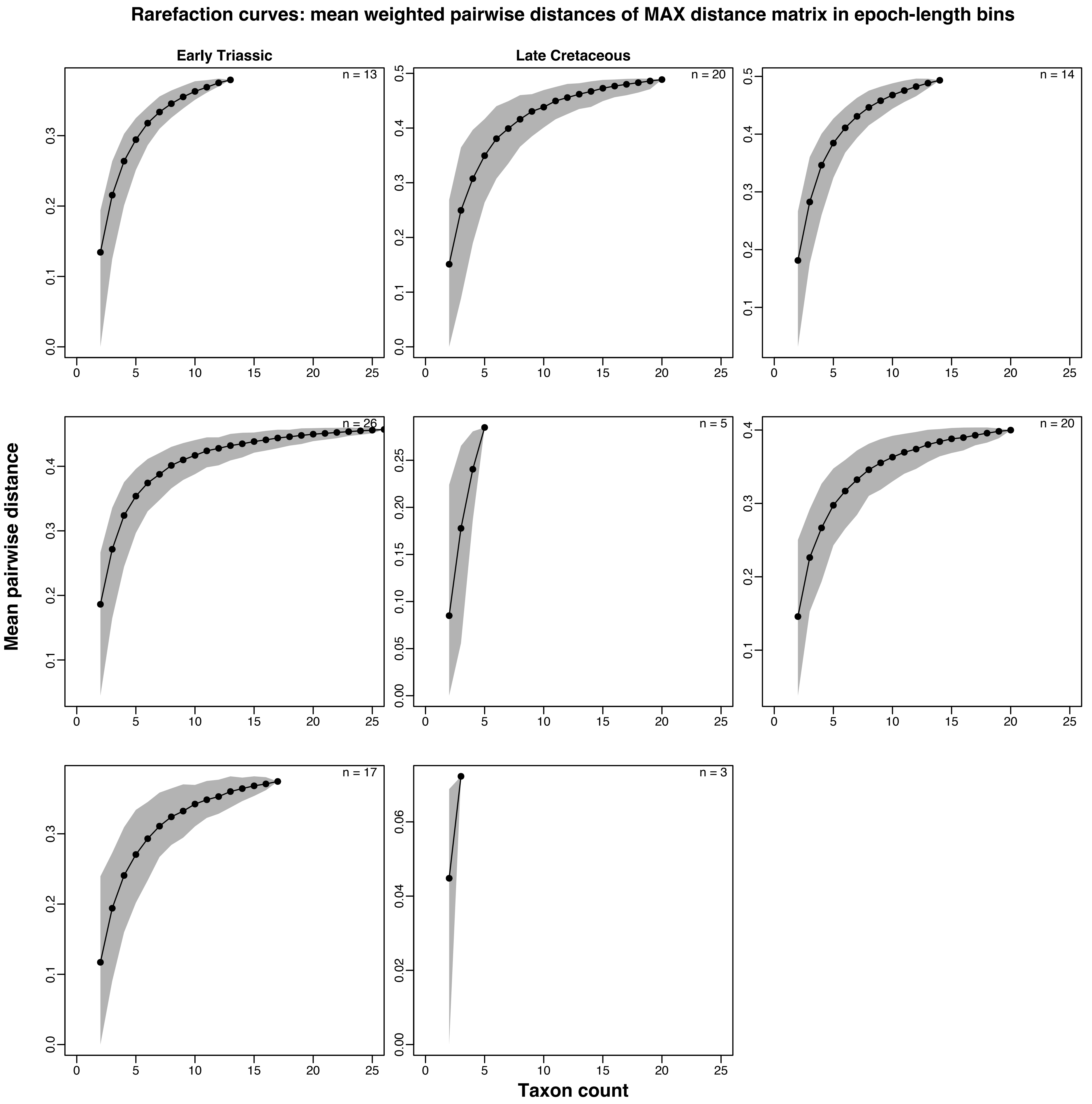
Rarefaction curves: mean weighted pairwise distances of MAX distance matrix in epoch-length bins
Early Triassic
Late Cretaceous
n = 13
n = 20
n = 14
n = 26
n = 5
n = 20
n = 17
n = 3
Mean pairwise distance
Taxon count

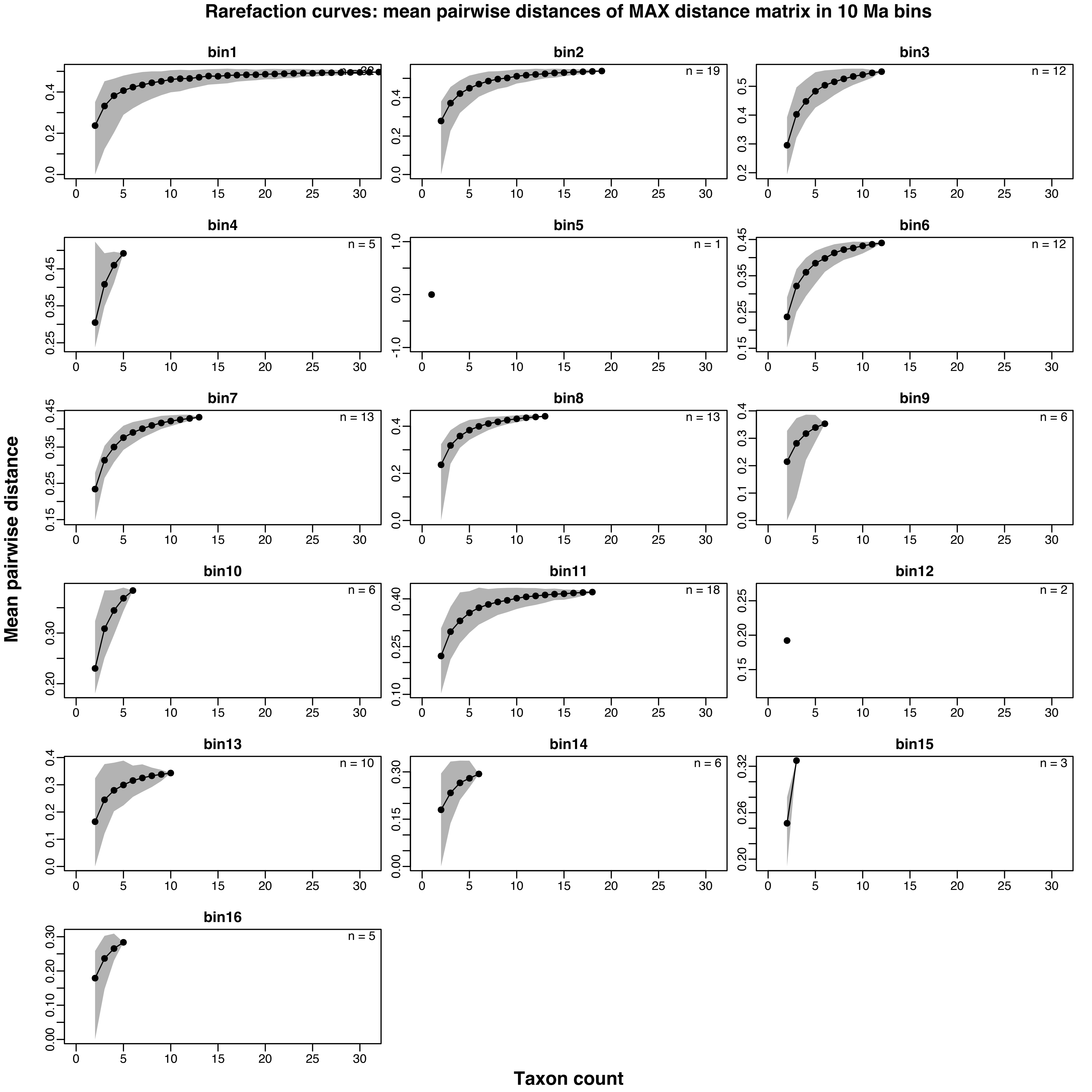

Rarefaction curves: mean pairwise distances of MAX distance matrix in 10 Ma bins
bin1
bin2
n = 19
bin3
n = 12
bin4
n = 5
bin5
n = 1
bin6
n = 12
bin7
n = 13
bin8
n = 13
bin9
n = 6
bin10
n = 6
bin11
n = 18
bin12
n = 2
bin13
n = 10
bin14
n = 6
bin15
n = 3
bin16
n = 5
Mean pairwise distance
Taxon count

Rarefaction curves: mean weighted pairwise distances of MAX distance matrix in 10 Ma bins

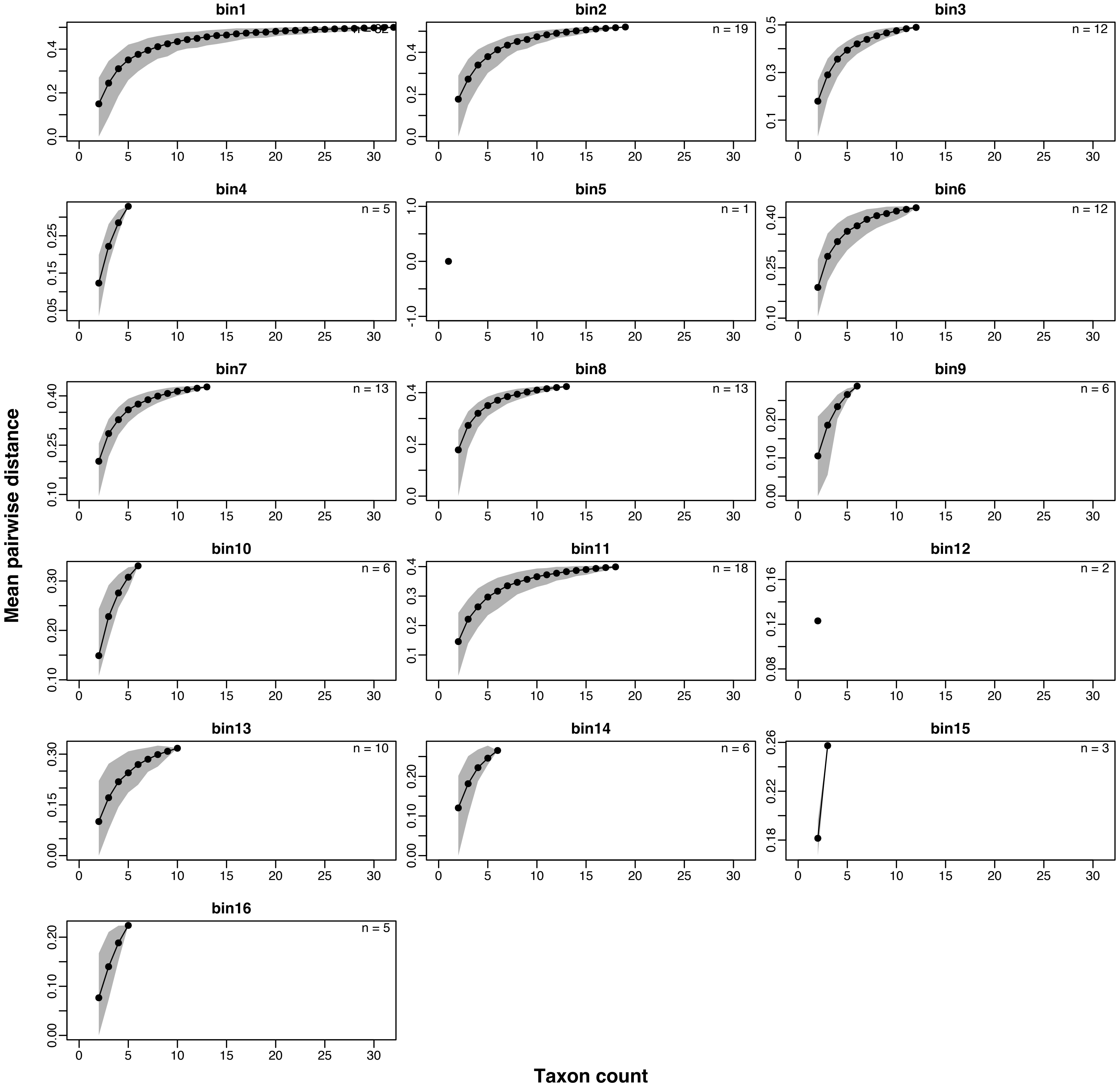

Supplementary figure 3. **Cumulative variance described by axes of the ordinated data and the correlation of these axes with the original data.** Axes from principal coordinates analysis of the four distance matrices used here derived from the cladistic data set of Moon [1].

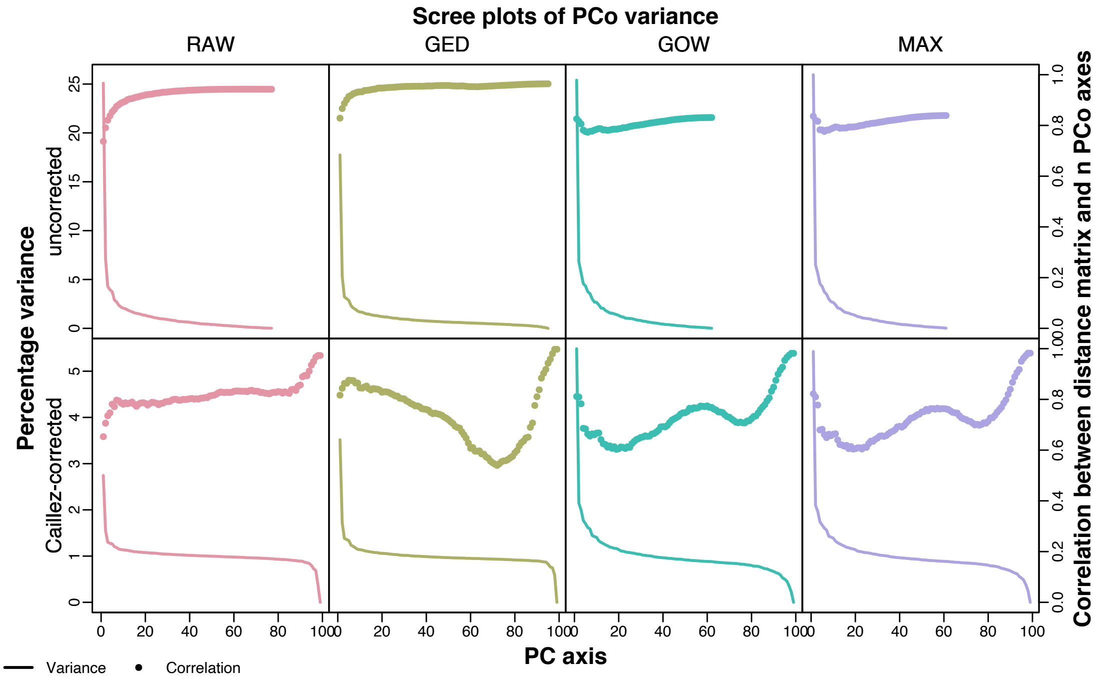

Supplementary figure 4. (following pages) **Per-bin discrete skeletal disparity of Ichthyosauriformes through the Mesozoic from ordinated data.** Ichthyosaur disparity represented by mean sum of variances, mean sum of ranges, and mean centroid distance from each of eight PCA (four distance matrices: RAW, GED, GOW, MAX; with and without negative eigenvalue correction) on the cladistic matrix of Moon [1]. Error bars show 95% confidence intervals from 500 bootstrap replicates.

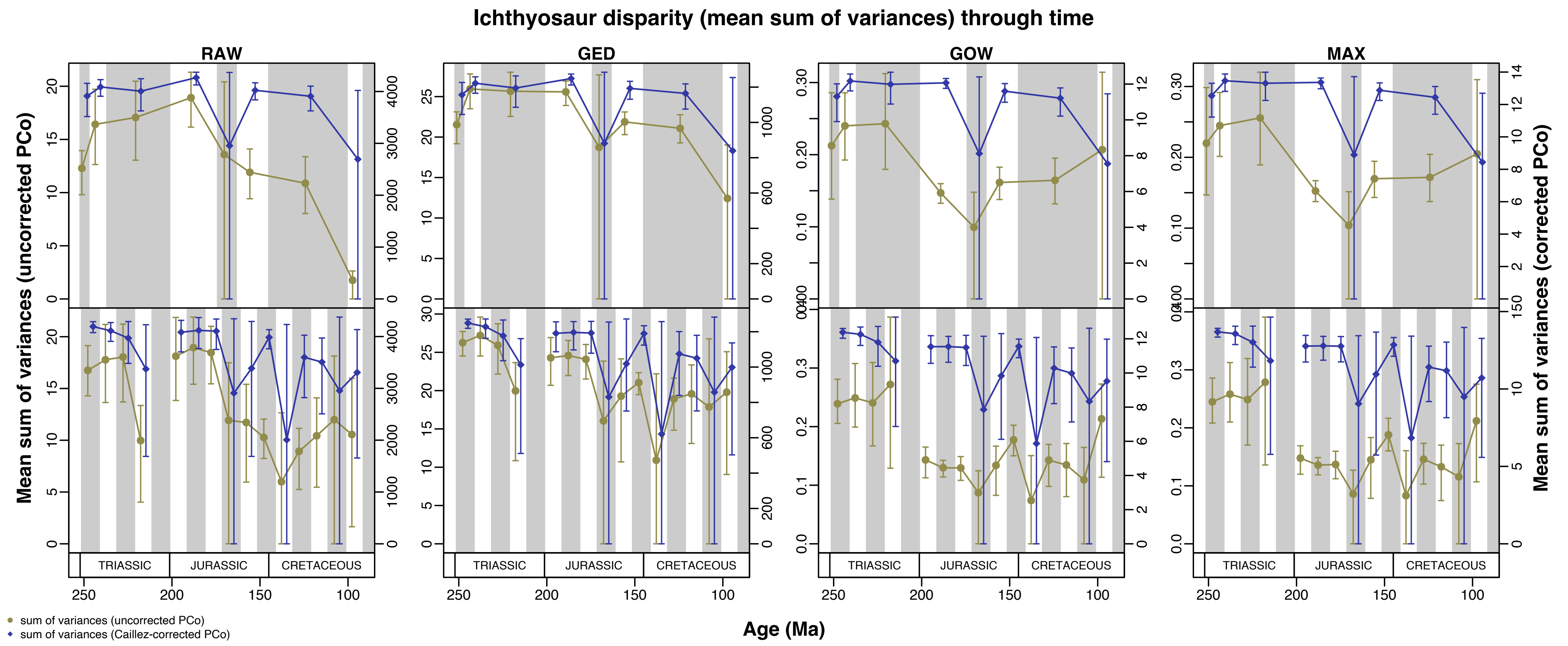

Ichthyosaur disparity (mean sum of variances) through time
RAW
GED
GOW
MAX
Mean sum of variances (uncorrected PCo)
Mean sum of variances (corrected PCo)
TRIASSIC
JURASSIC
CRETACEOUS
Age (Ma)
sum of variances (uncorrected PCo)
sum of variances (Caillez-corrected PCo)

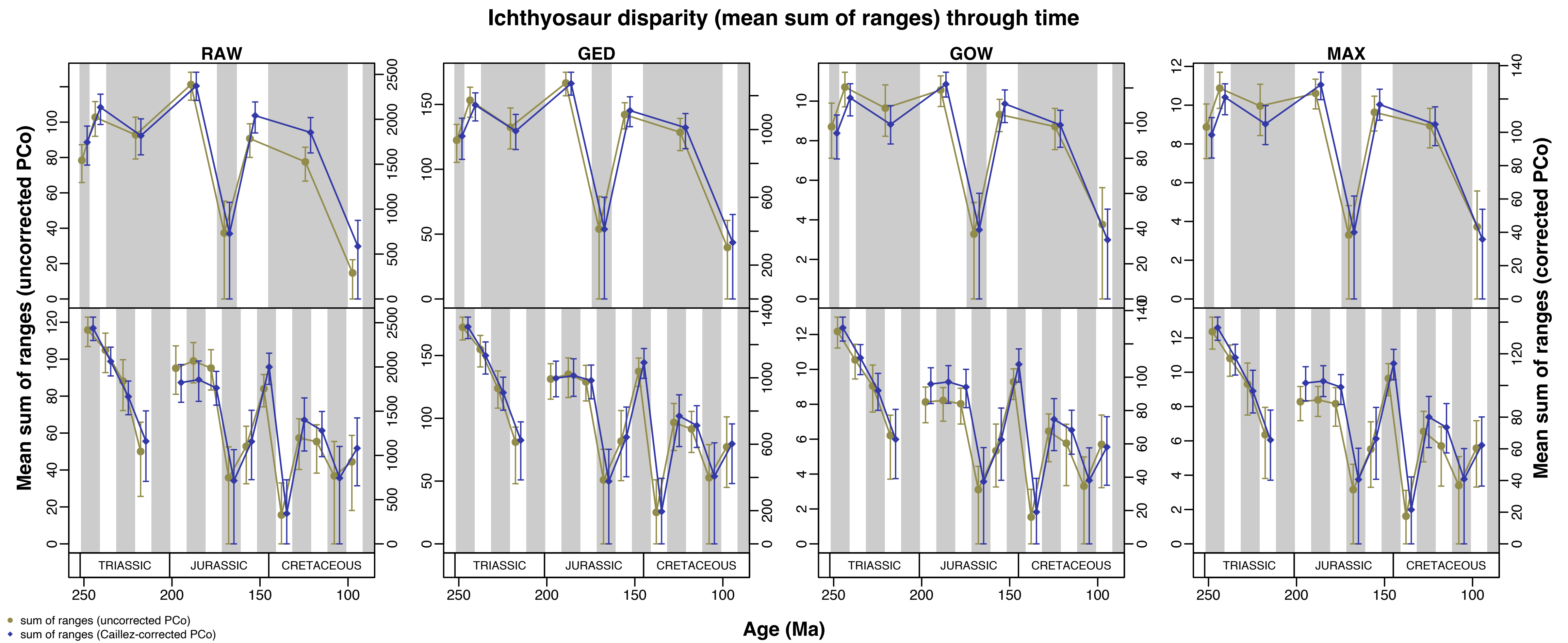
Ichthyosaur disparity (mean sum of ranges) through time
RAW
GED
GOW
MAX
Mean sum of ranges (uncorrected PCo)
Mean sum of ranges (corrected PCo)
TRIASSIC
JURASSIC
CRETACEOUS
Age (Ma)
sum of ranges (uncorrected PCo)
sum of ranges (Caillez-corrected PCo)

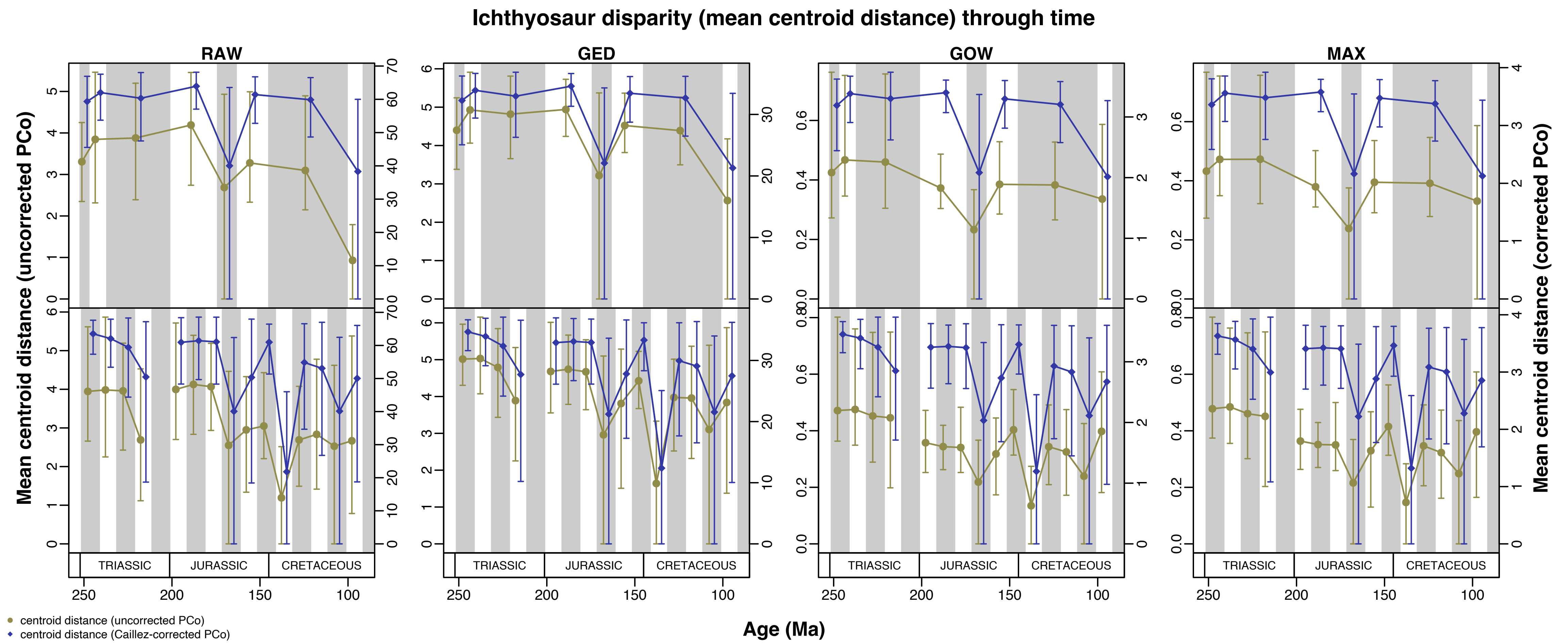

Ichthyosaur disparity (mean centroid distance) through time
RAW
GED
GOW
MAX
Mean centroid distance (uncorrected PCo)
Mean centroid distance (corrected PCo)
TRIASSIC
JURASSIC
CRETACEOUS
Age (Ma)
centroid distance (uncorrected PCo)
centroid distance (Caillez-corrected PCo)

Supplementary figure 5. (following pages) **Per-bin rarefaction curves for each disparity-time curve shown in Supplementary Figure 4** Disparity for each bin is sequentially rarefied on taxon occurrences. Error polygon gives 95% confidence interval from 500 replicates.

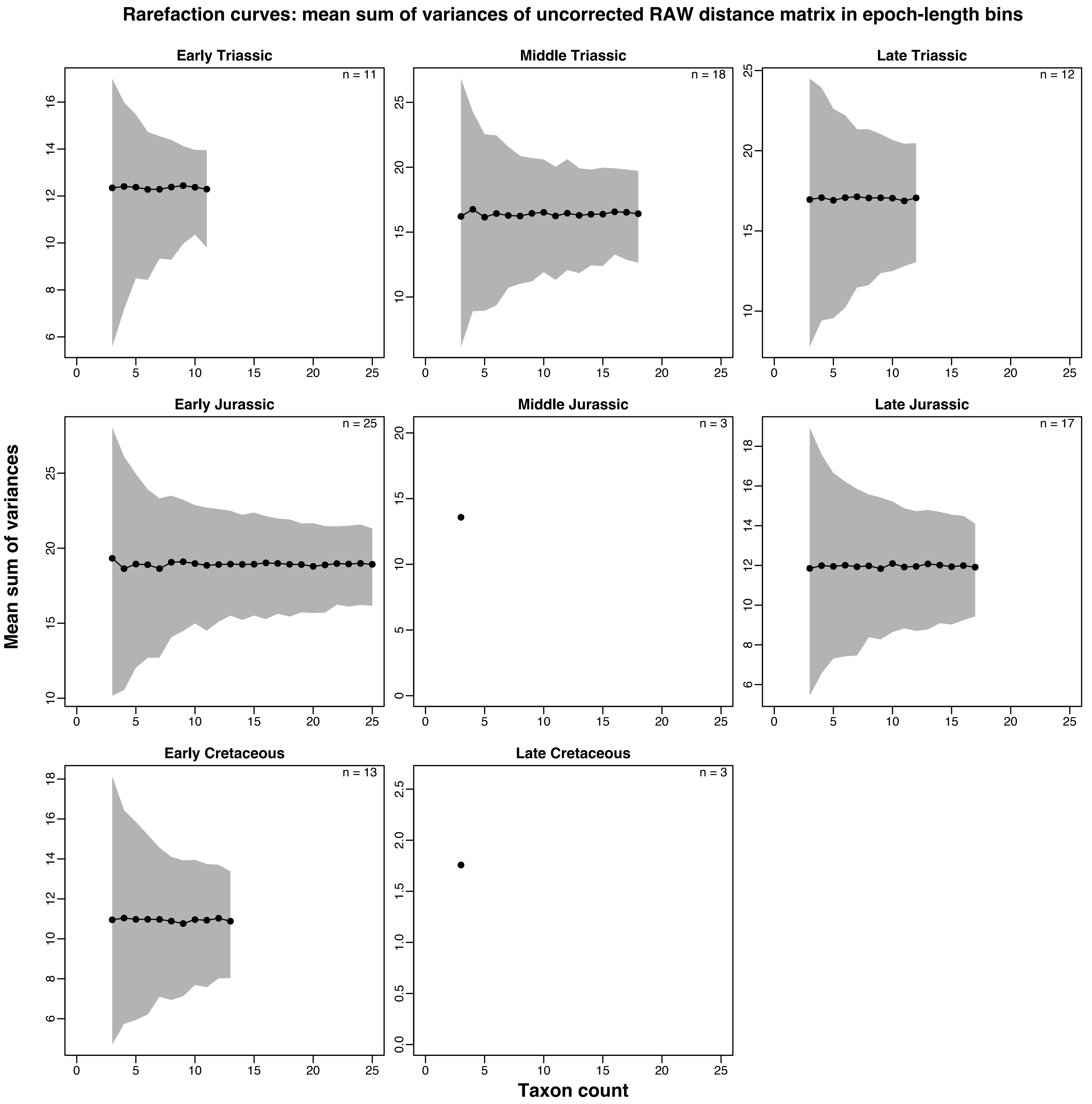
Rarefaction curves: mean sum of variances of uncorrected RAW distance matrix in epoch-length bins
Early Triassic
n = 11
Middle Triassic
n = 18
Late Triassic
n = 12
Early Jurassic
n = 25
Middle Jurassic
n = 3
Late Jurassic
n = 17
Early Cretaceous
n = 13
Late Cretaceous
n = 3
Mean sum of variances
Taxon count

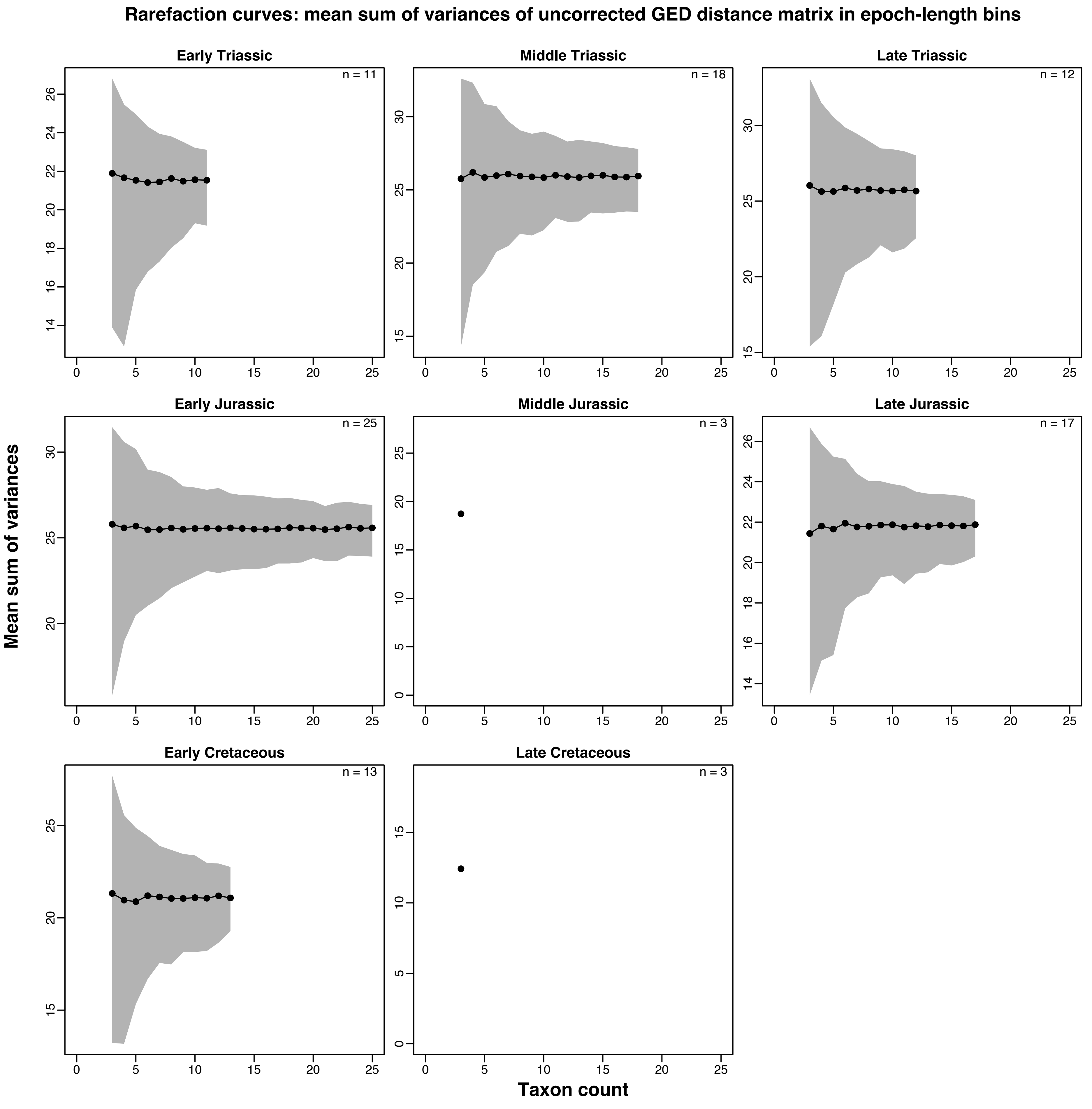

Rarefaction curves: mean sum of variances of uncorrected GED distance matrix in epoch-length bins
Early Triassic
n = 11
Middle Triassic
n = 18
Late Triassic
n = 12
Early Jurassic
n = 25
Middle Jurassic
n = 3
Late Jurassic
n = 17
Early Cretaceous
n = 13
Late Cretaceous
n = 3
Mean sum of variances
Taxon count

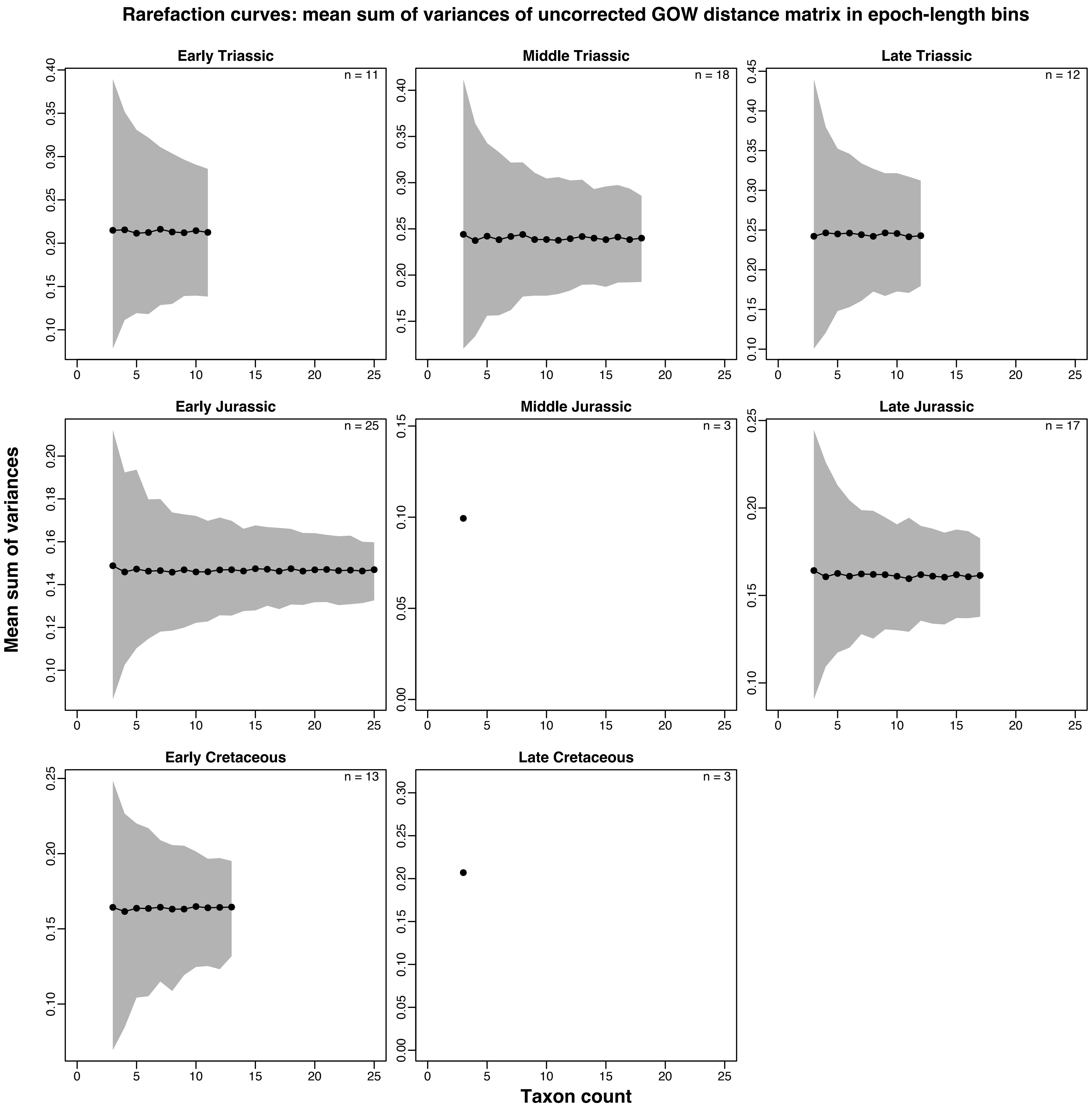

Rarefaction curves: mean sum of variances of uncorrected GOW distance matrix in epoch-length bins
Early Triassic
n = 11
Middle Triassic
n = 18
Late Triassic
n = 12
Early Jurassic
n = 25
Middle Jurassic
n = 3
Late Jurassic
n = 17
Early Cretaceous
n = 13
Late Cretaceous
n = 3
Mean sum of variances
Taxon count

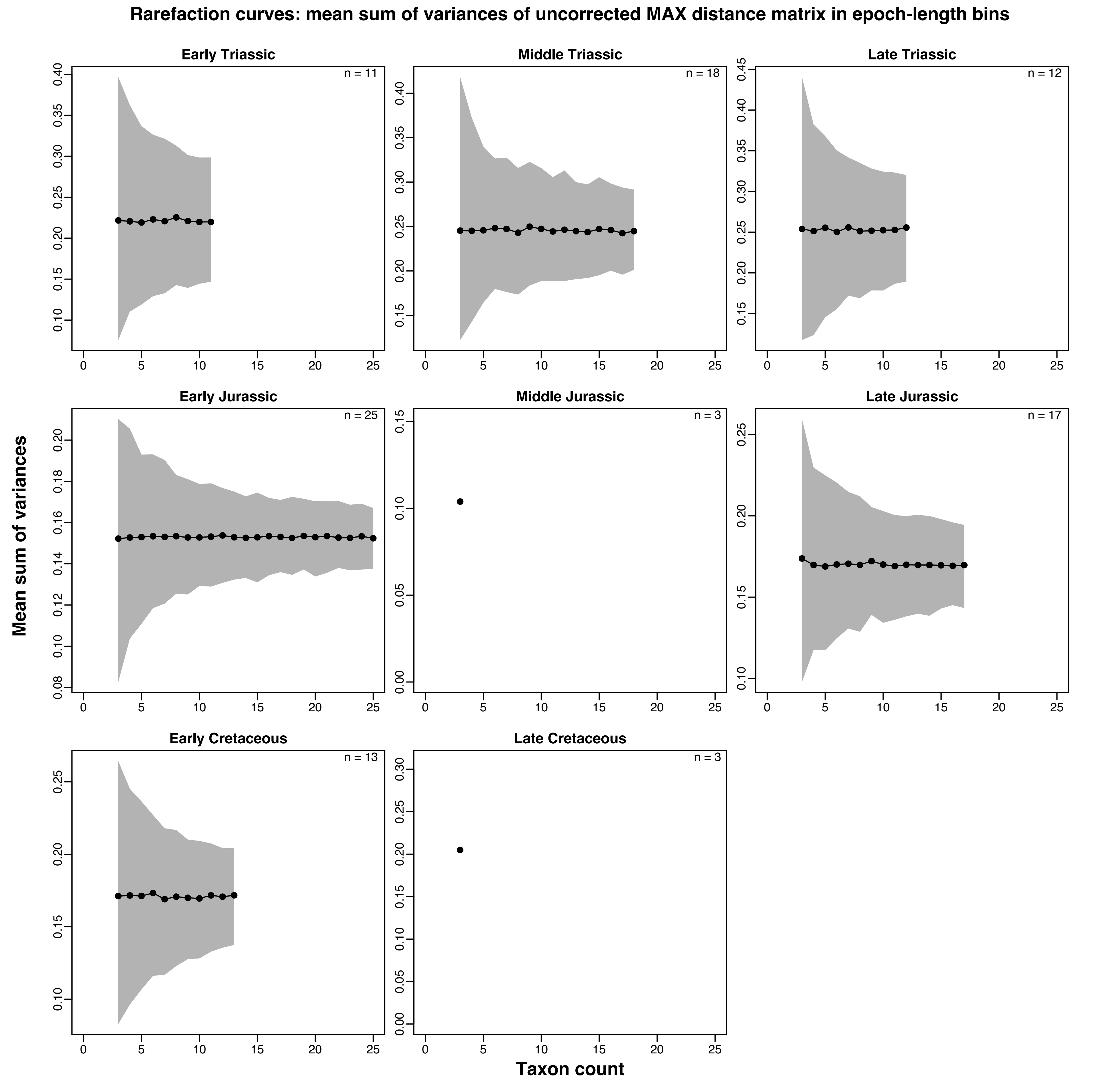

Rarefaction curves: mean sum of variances of uncorrected MAX distance matrix in epoch-length bins
Early Triassic
n = 11
Middle Triassic
n = 18
Late Triassic
n = 12
Early Jurassic
n = 25
Middle Jurassic
n = 3
Late Jurassic
n = 17
Early Cretaceous
n = 13
Late Cretaceous
n = 3
Mean sum of variances
Taxon count

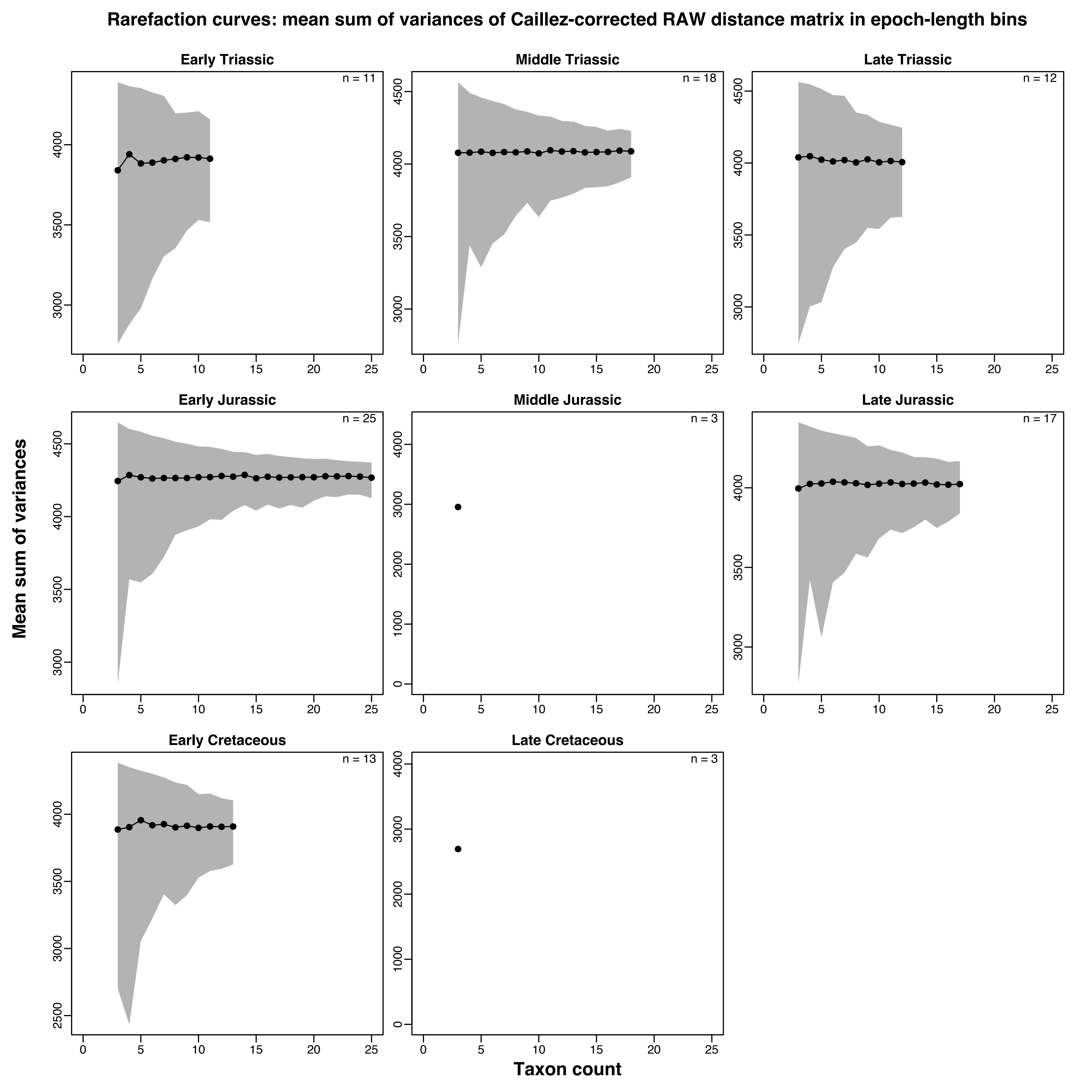
Rarefaction curves: mean sum of variances of Caillez-corrected RAW distance matrix in epoch-length bins
Early Triassic
n = 11
Middle Triassic
n = 18
Late Triassic
n = 12
Early Jurassic
n = 25
Middle Jurassic
n = 3
Late Jurassic
n = 17
Early Cretaceous
n = 13
Late Cretaceous
n = 3
Mean sum of variances
Taxon count

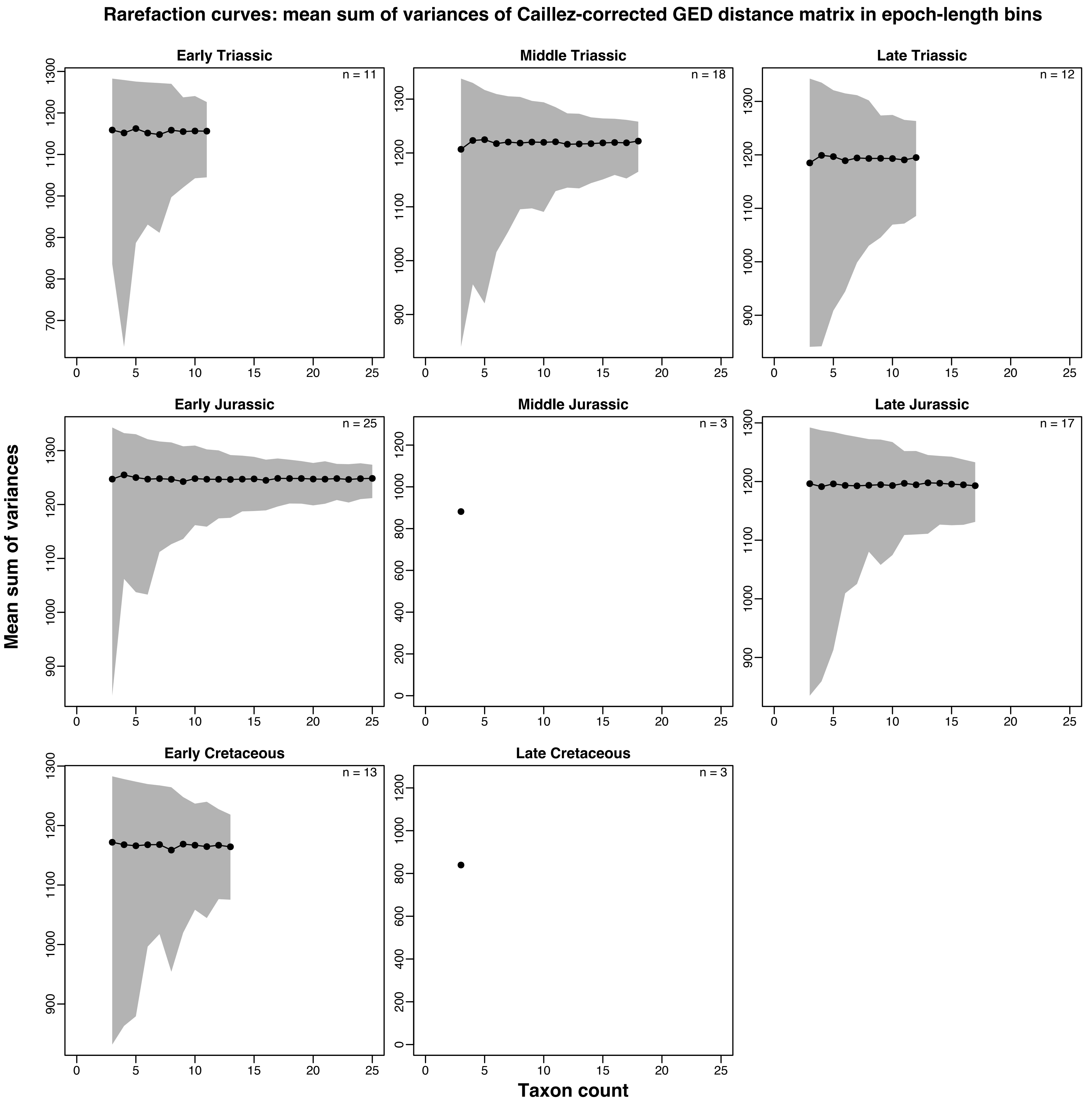

Rarefaction curves: mean sum of variances of Caillez-corrected GED distance matrix in epoch-length bins
Early Triassic
n = 11
Middle Triassic
n = 18
Late Triassic
n = 12
Early Jurassic
n = 25
Middle Jurassic
n = 3
Late Jurassic
n = 17
Early Cretaceous
n = 13
Late Cretaceous
n = 3
Mean sum of variances
Taxon count

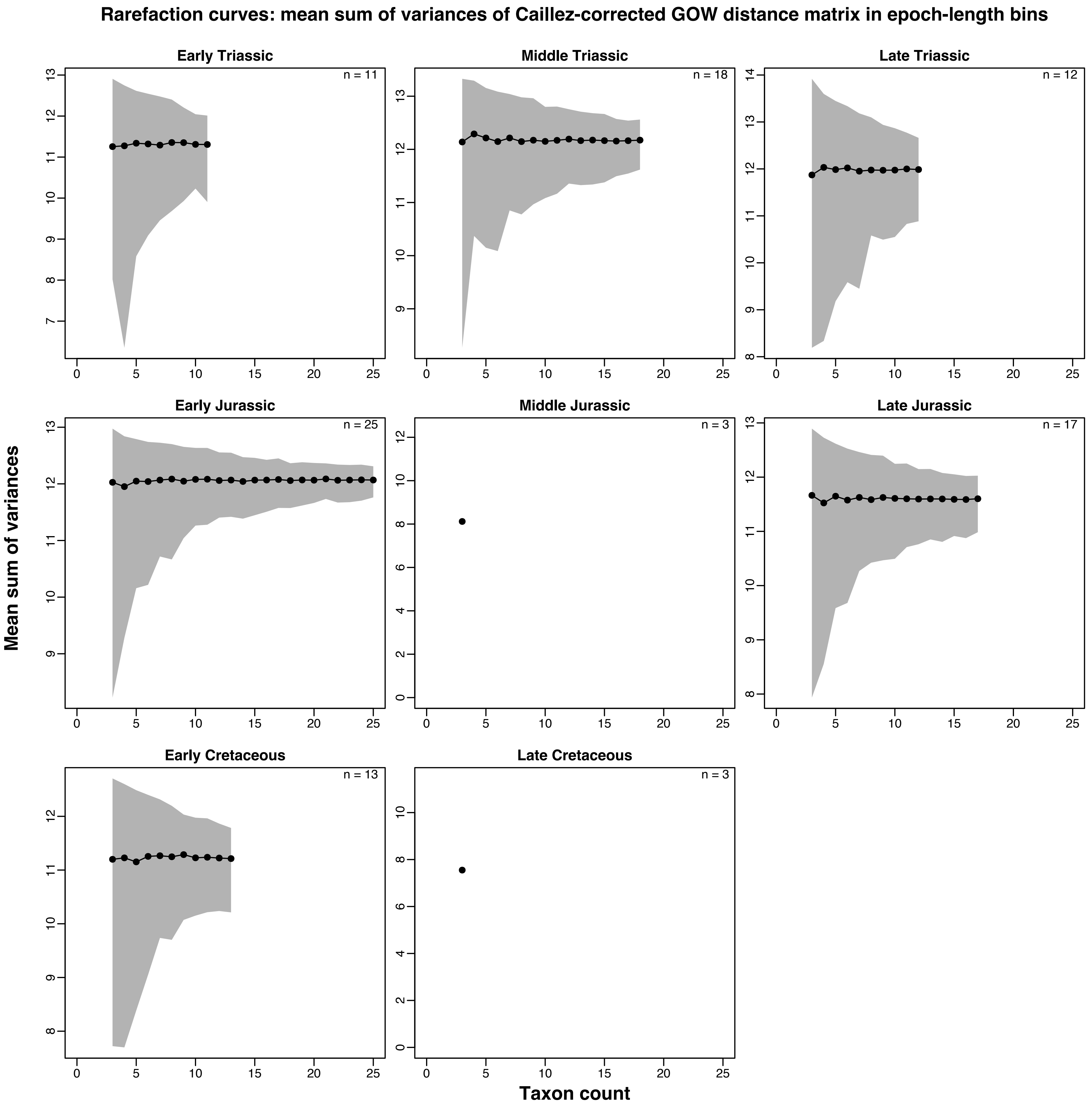

Rarefaction curves: mean sum of variances of Caillez-corrected GOW distance matrix in epoch-length bins
Early Triassic
n = 11
Middle Triassic
n = 18
Late Triassic
n = 12
Early Jurassic
n = 25
Middle Jurassic
n = 3
Late Jurassic
n = 17
Early Cretaceous
n = 13
Late Cretaceous
n = 3
Mean sum of variances
Taxon count

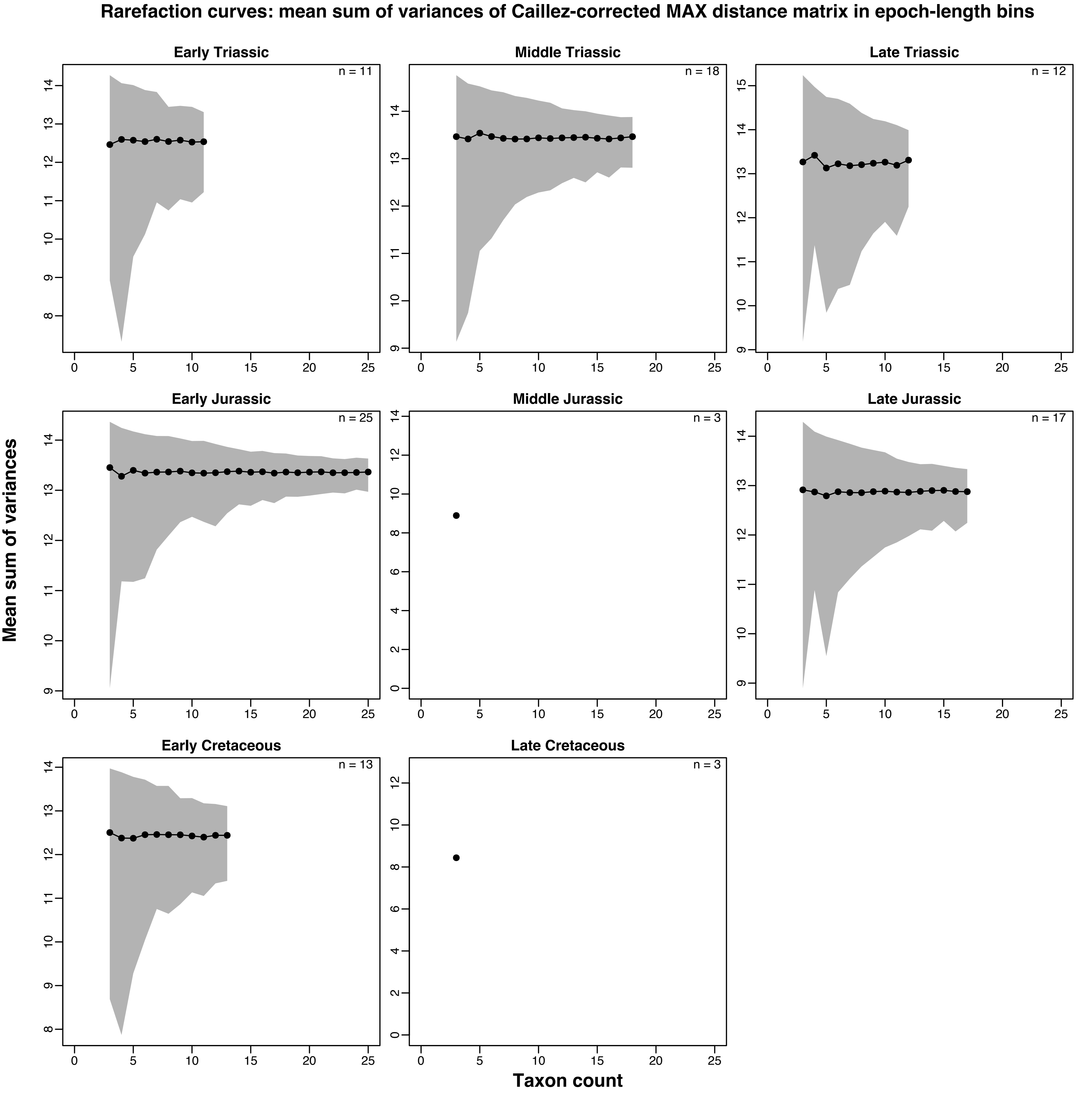

Rarefaction curves: mean sum of variances of Caillez-corrected MAX distance matrix in epoch-length bins
Early Triassic
n = 11
Middle Triassic
n = 18
Late Triassic
n = 12
Early Jurassic
n = 25
Middle Jurassic
n = 3
Late Jurassic
n = 17
Early Cretaceous
n = 13
Late Cretaceous
n = 3
Mean sum of variances
Taxon count

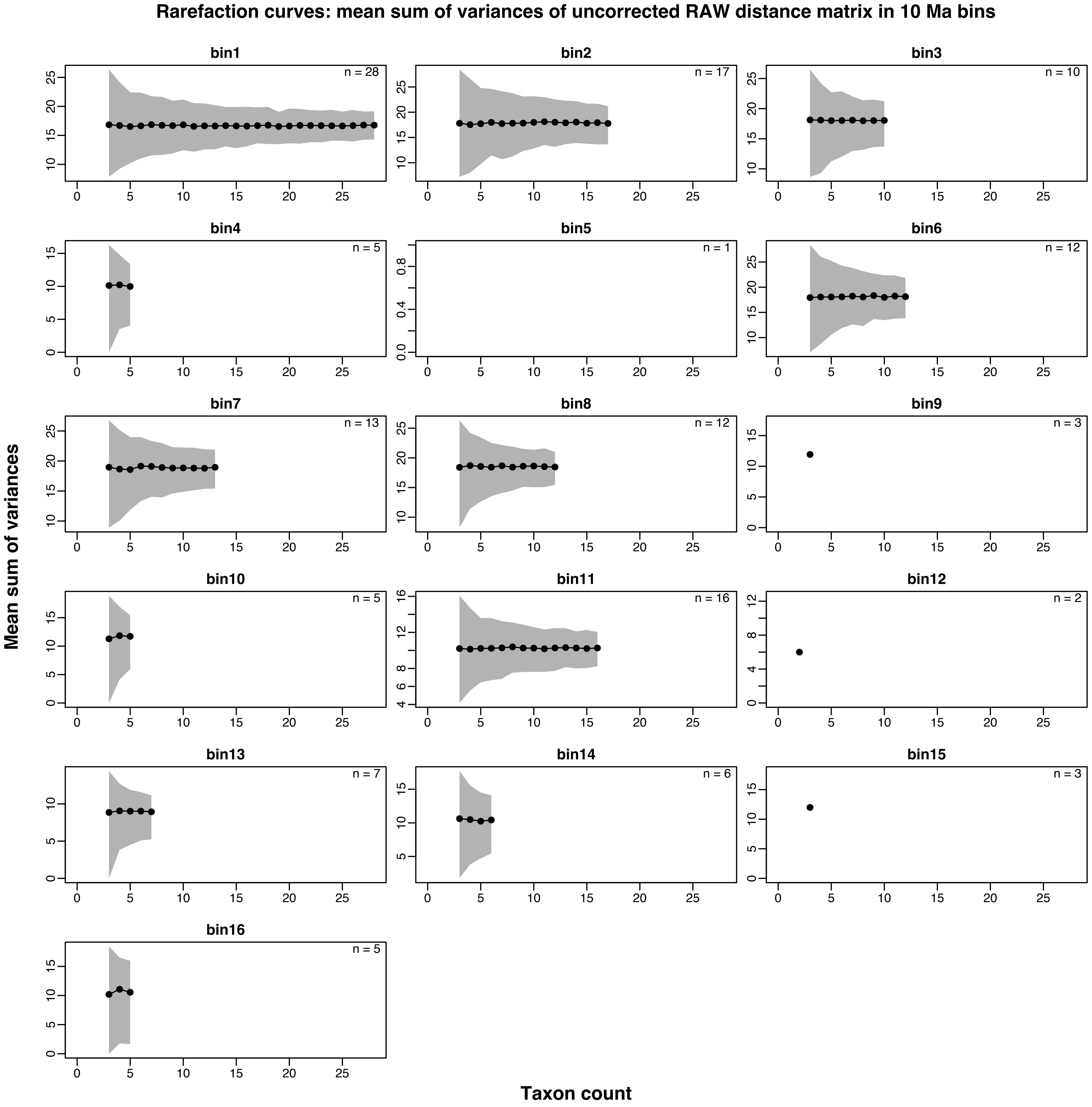
Rarefaction curves: mean sum of variances of uncorrected RAW distance matrix in 10 Ma bins
bin1
n = 28
bin2
n = 17
bin3
n = 10
bin4
n = 5
bin5
n = 1
bin6
n = 12
bin7
n = 13
bin8
n = 12
bin9
n = 3
bin10
n = 5
bin11
n = 16
bin12
n = 2
bin13
n = 7
bin14
n = 6
bin15
n = 3
bin16
n = 5
Mean sum of variances
Taxon count

Rarefaction curves: mean sum of variances of uncorrected GED distance matrix in 10 Ma bins

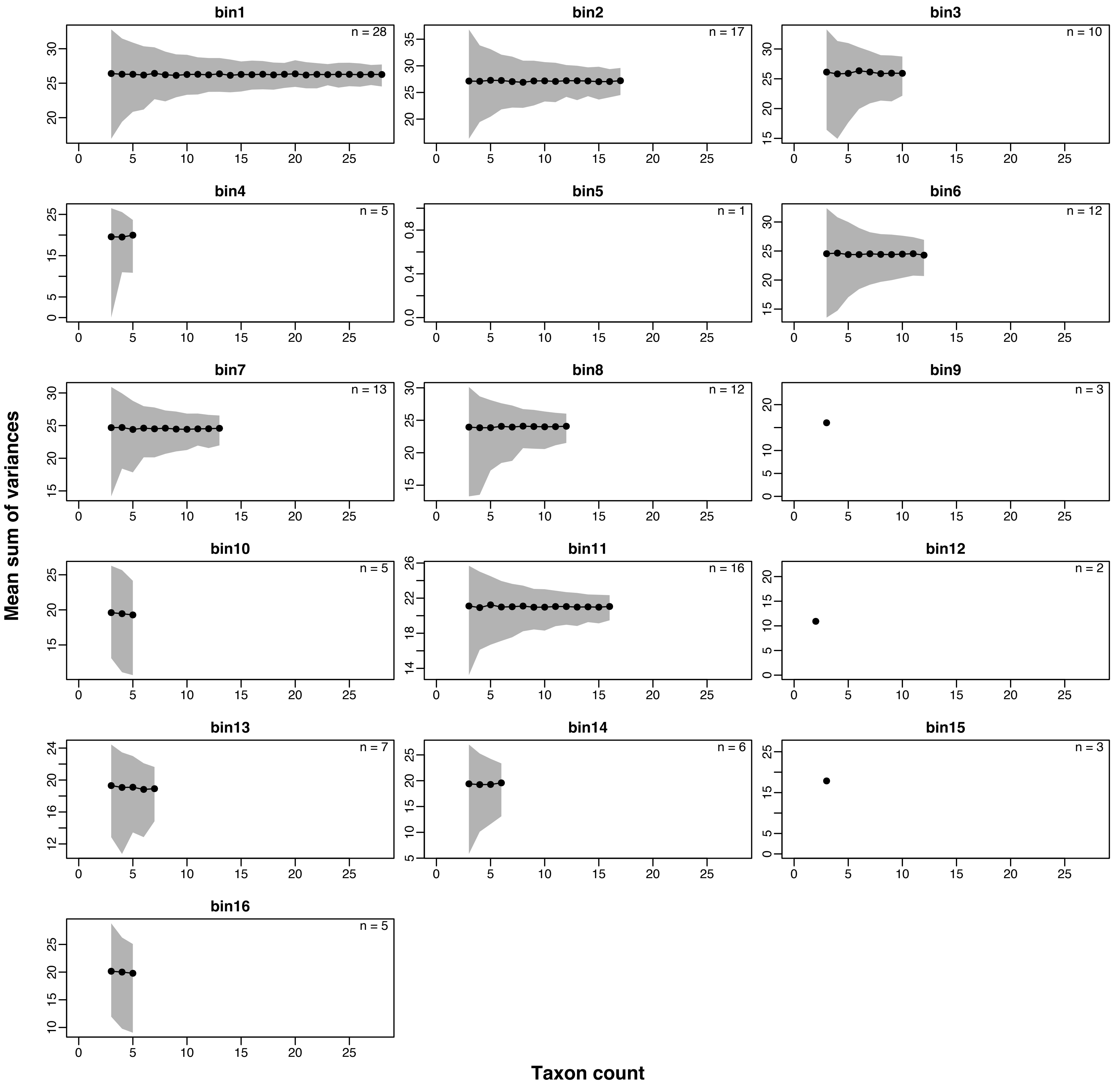

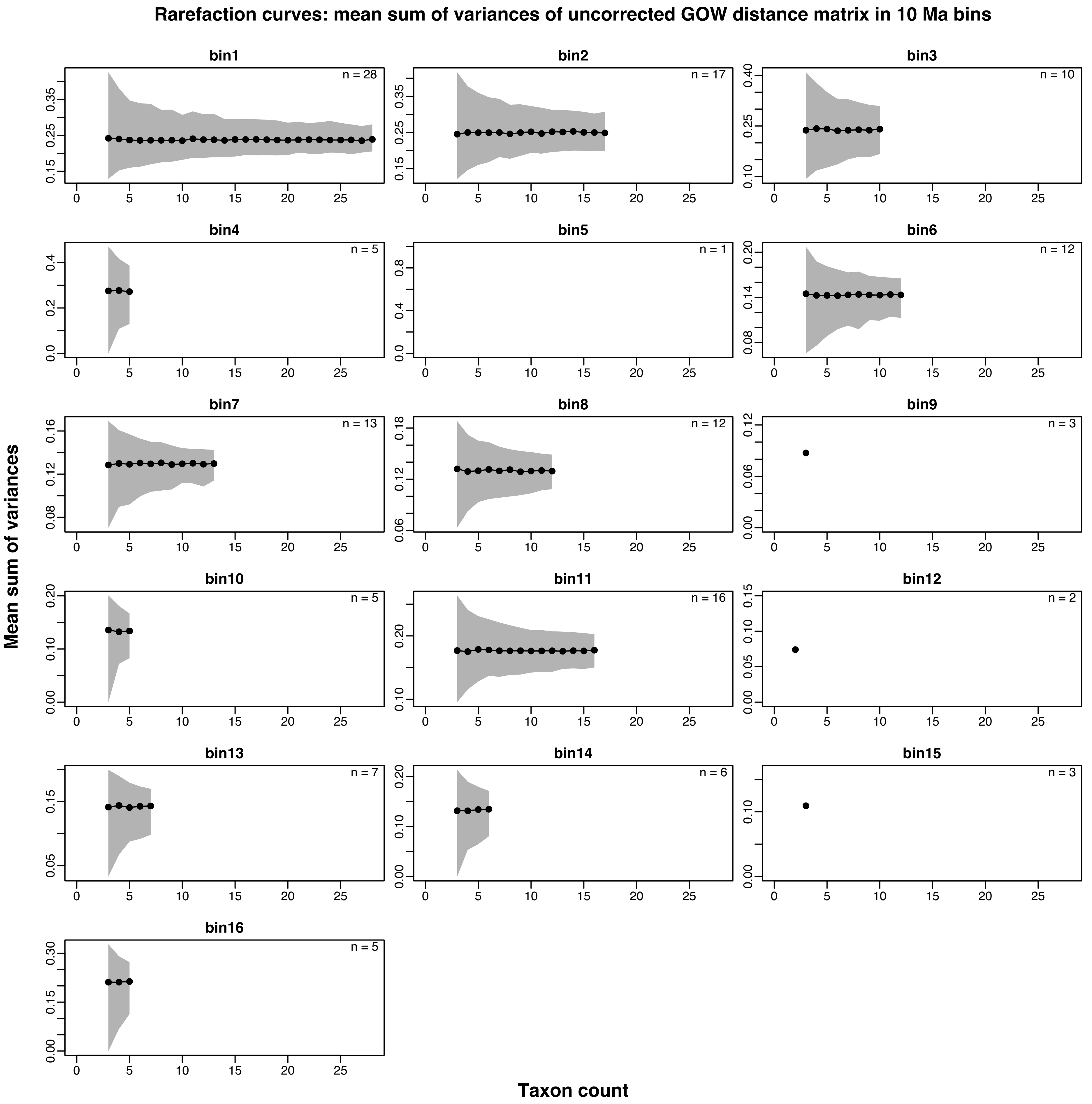

Rarefaction curves: mean sum of variances of uncorrected GOW distance matrix in 10 Ma bins
bin1
n = 28
bin2
n = 17
bin3
n = 10
bin4
n = 5
bin5
n = 1
bin6
n = 12
bin7
n = 13
bin8
n = 12
bin9
n = 3
bin10
n = 5
bin11
n = 16
bin12
n = 2
bin13
n = 7
bin14
n = 6
bin15
n = 3
bin16
n = 5
Mean sum of variances
Taxon count

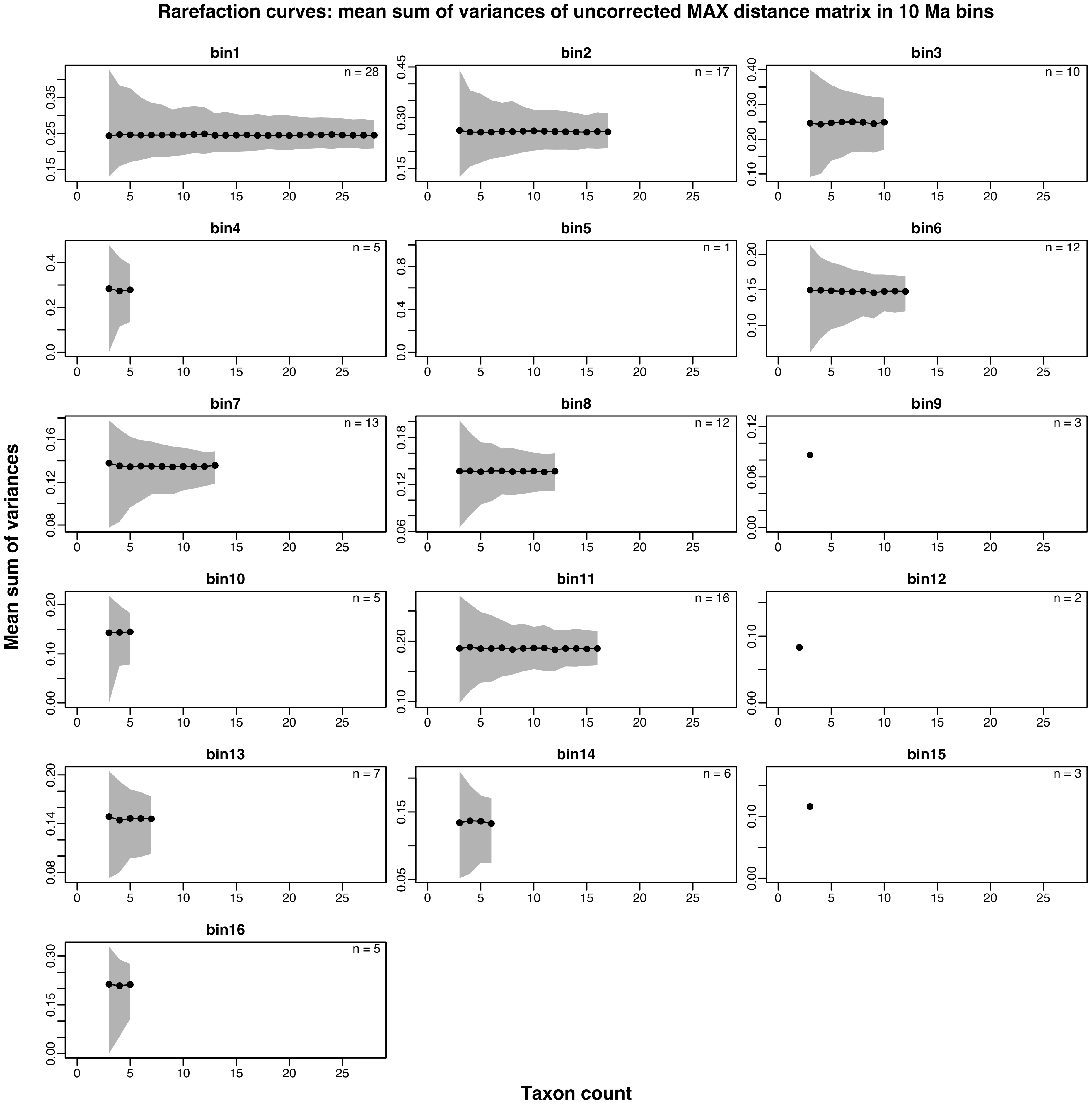
Rarefaction curves: mean sum of variances of uncorrected MAX distance matrix in 10 Ma bins
bin1
n = 28
bin2
n = 17
bin3
n = 10
bin4
n = 5
bin5
n = 1
bin6
n = 12
bin7
n = 13
bin8
n = 12
bin9
n = 3
bin10
n = 5
bin11
n = 16
bin12
n = 2
bin13
n = 7
bin14
n = 6
bin15
n = 3
bin16
n = 5
Mean sum of variances
Taxon count

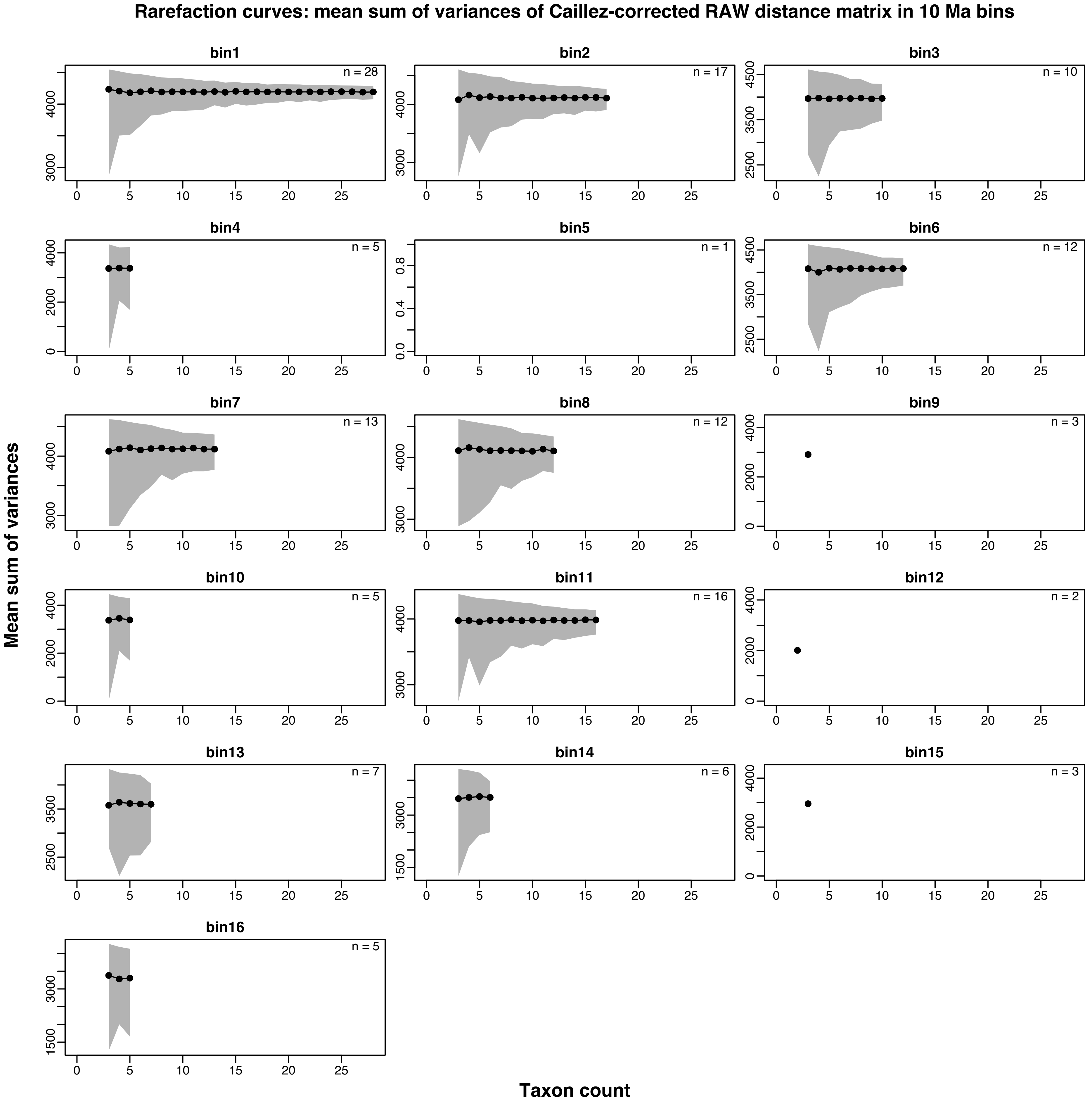

Rarefaction curves: mean sum of variances of Caillez-corrected RAW distance matrix in 10 Ma bins
bin1
n = 28
bin2
n = 17
bin3
n = 10
bin4
n = 5
bin5
n = 1
bin6
n = 12
bin7
n = 13
bin8
n = 12
bin9
n = 3
bin10
n = 5
bin11
n = 16
bin12
n = 2
bin13
n = 7
bin14
n = 6
bin15
n = 3
bin16
n = 5
Mean sum of variances
Taxon count

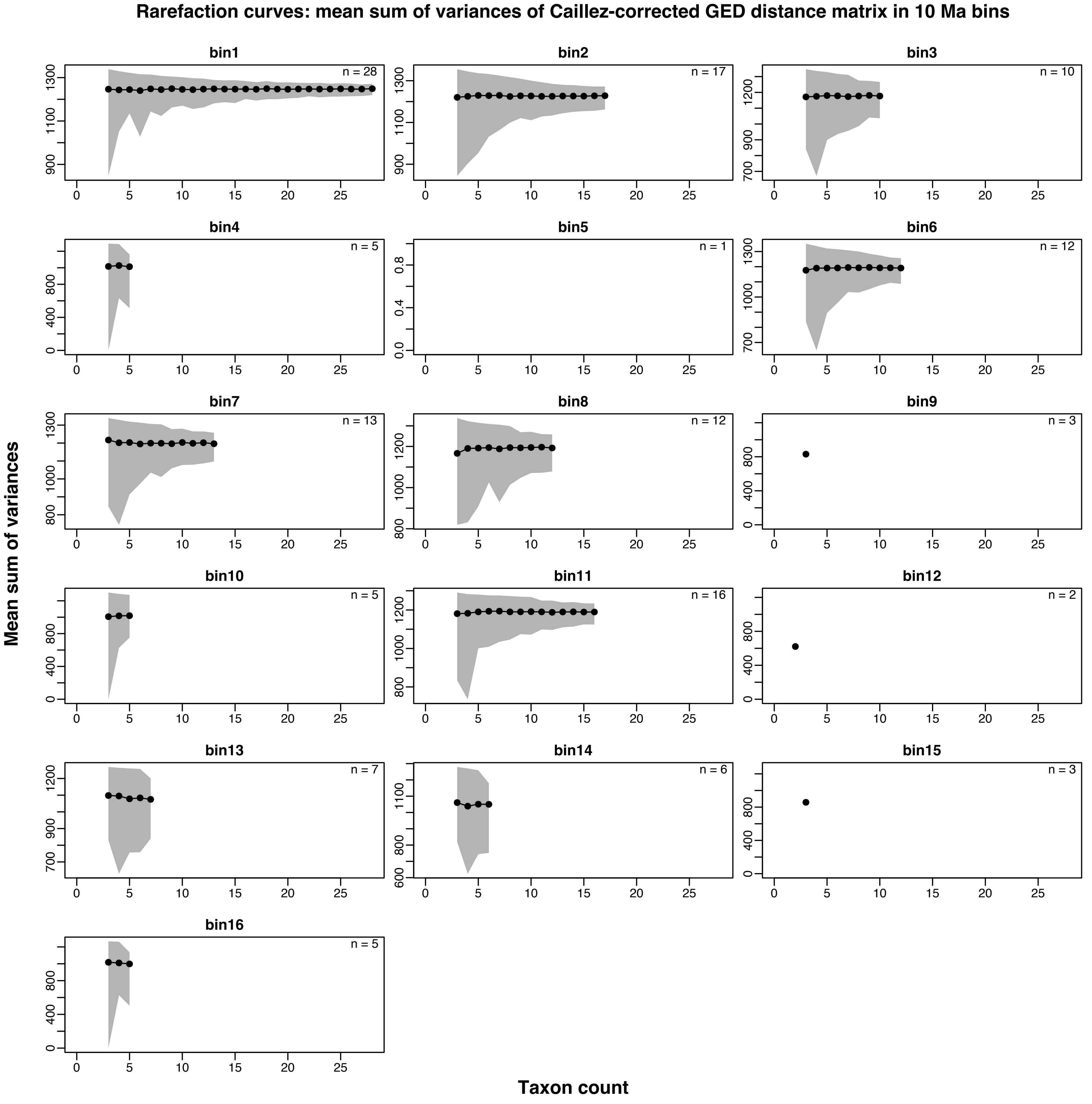

Rarefaction curves: mean sum of variances of Caillez-corrected GED distance matrix in 10 Ma bins
bin1
n = 28
bin2
n = 17
bin3
n = 10
bin4
n = 5
bin5
n = 1
bin6
n = 12
bin7
n = 13
bin8
n = 12
bin9
n = 3
bin10
n = 5
bin11
n = 16
bin12
n = 2
bin13
n = 7
bin14
n = 6
bin15
n = 3
bin16
n = 5
Mean sum of variances
Taxon count

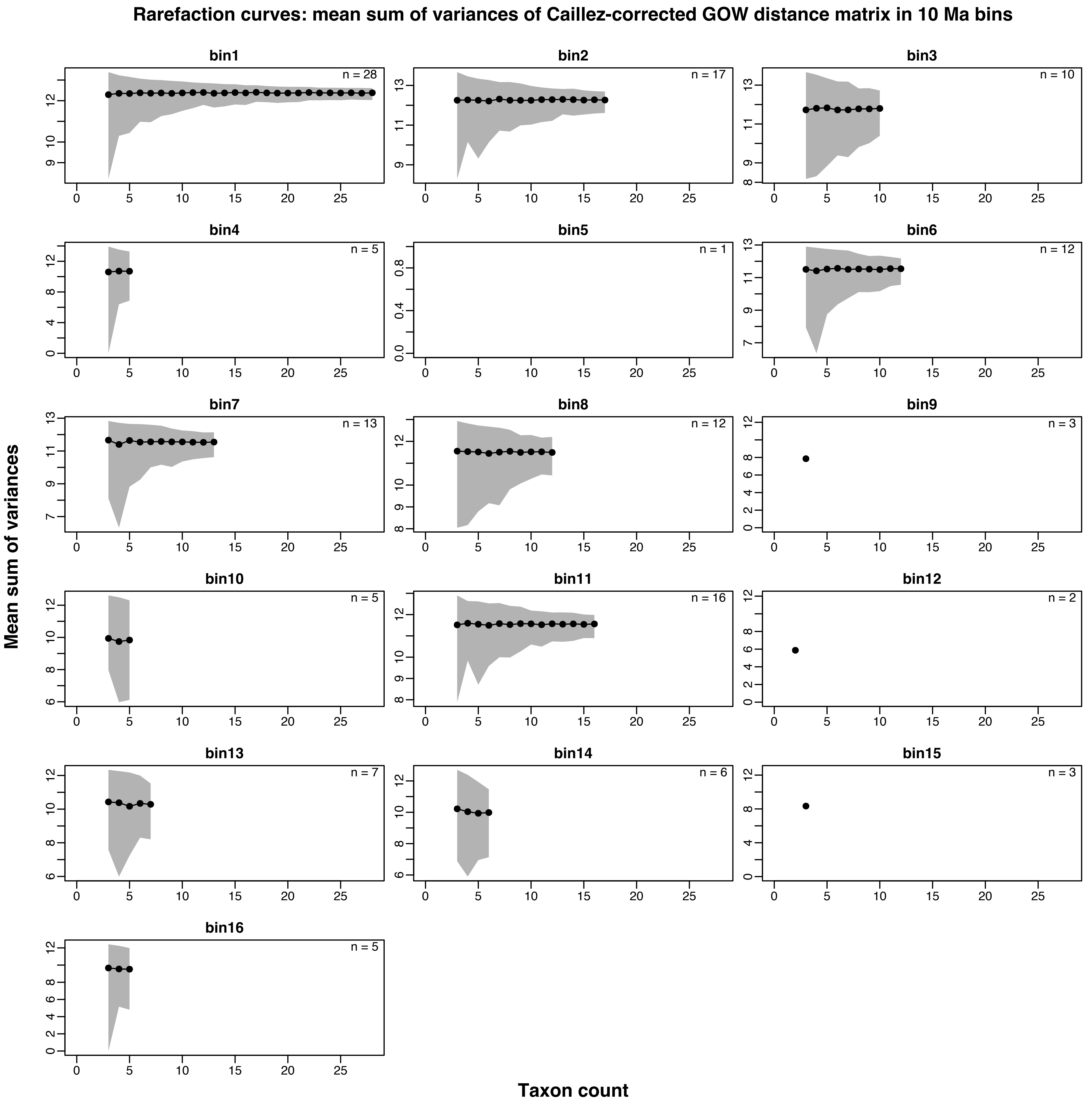
Rarefaction curves: mean sum of variances of Caillez-corrected GOW distance matrix in 10 Ma bins
bin1
n = 28
bin2
n = 17
bin3
n = 10
bin4
n = 5
bin5
n = 1
bin6
n = 12
bin7
n = 13
bin8
n = 12
bin9
n = 3
bin10
n = 5
bin11
n = 16
bin12
n = 2
bin13
n = 7
bin14
n = 6
bin15
n = 3
bin16
n = 5
Mean sum of variances
Taxon count

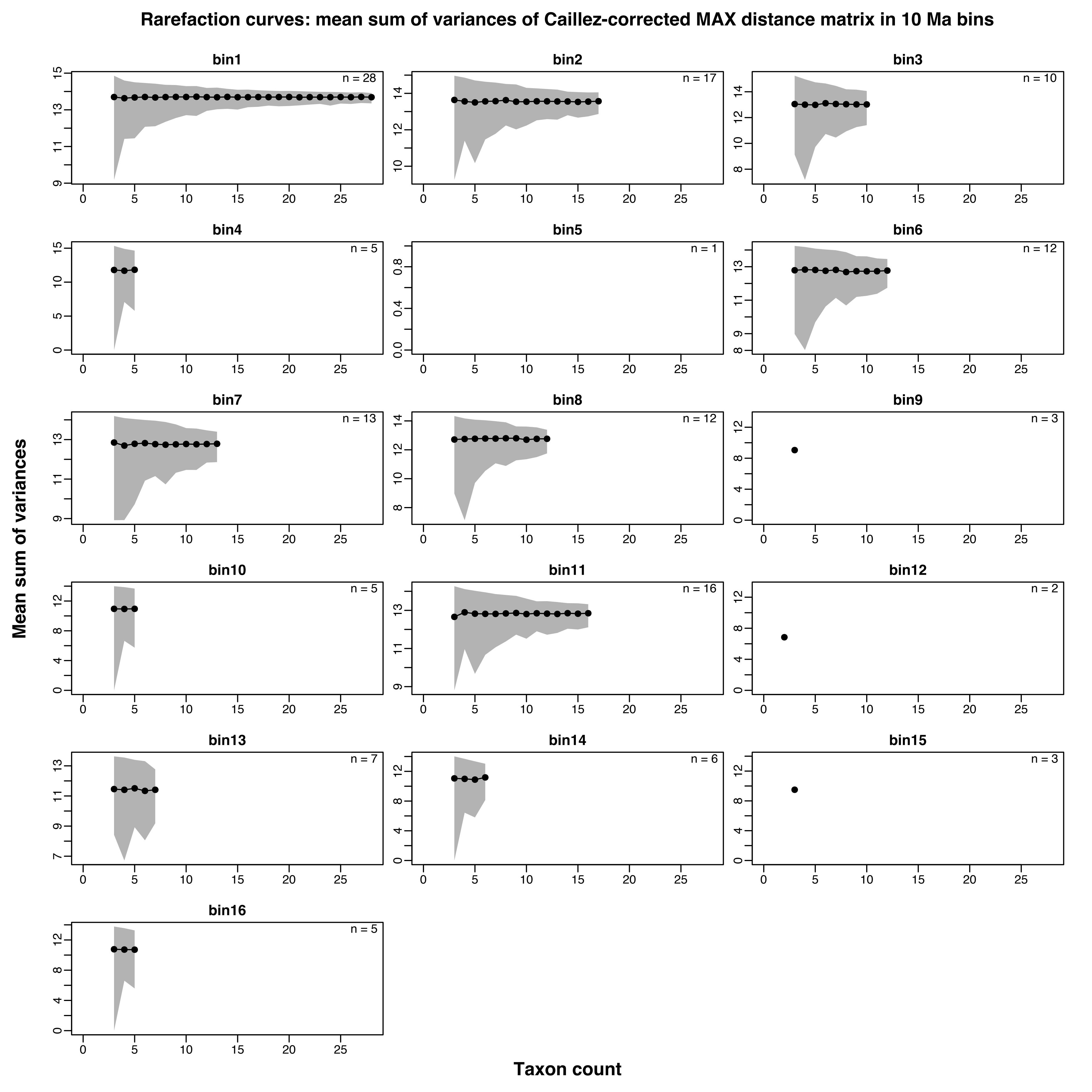

Rarefaction curves: mean sum of variances of Caillez-corrected MAX distance matrix in 10 Ma bins
bin1
n = 28
bin2
n = 17
bin3
n = 10
bin4
n = 5
bin5
n = 1
bin6
n = 12
bin7
n = 13
bin8
n = 12
bin9
n = 3
bin10
n = 5
bin11
n = 16
bin12
n = 2
bin13
n = 7
bin14
n = 6
bin15
n = 3
bin16
n = 5
Mean sum of variances
Taxon count

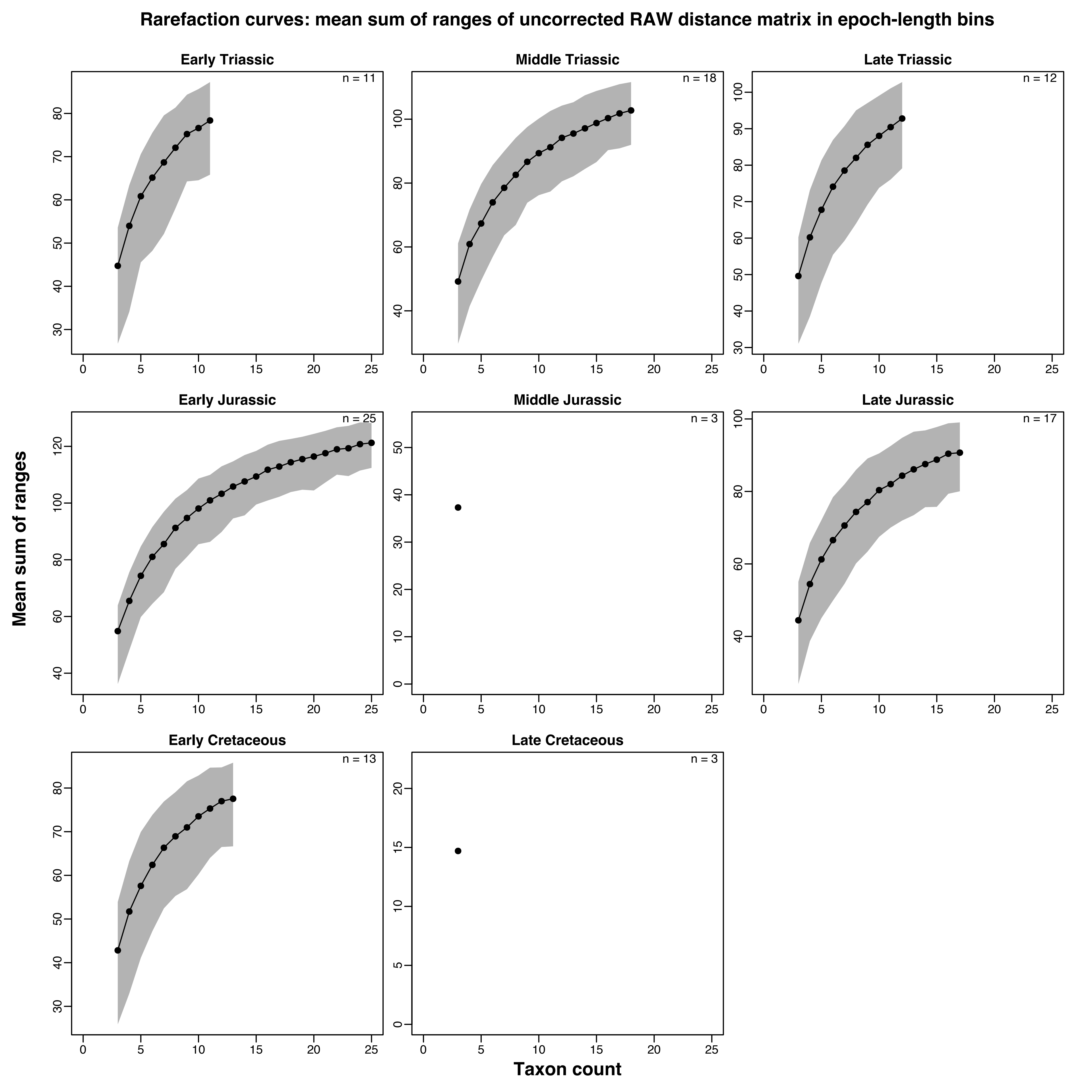

Rarefaction curves: mean sum of ranges of uncorrected RAW distance matrix in epoch-length bins
Early Triassic
n = 11
Middle Triassic
n = 18
Late Triassic
n = 12
Early Jurassic
n = 25
Middle Jurassic
n = 3
Late Jurassic
n = 17
Early Cretaceous
n = 13
Late Cretaceous
n = 3
Mean sum of ranges
Taxon count

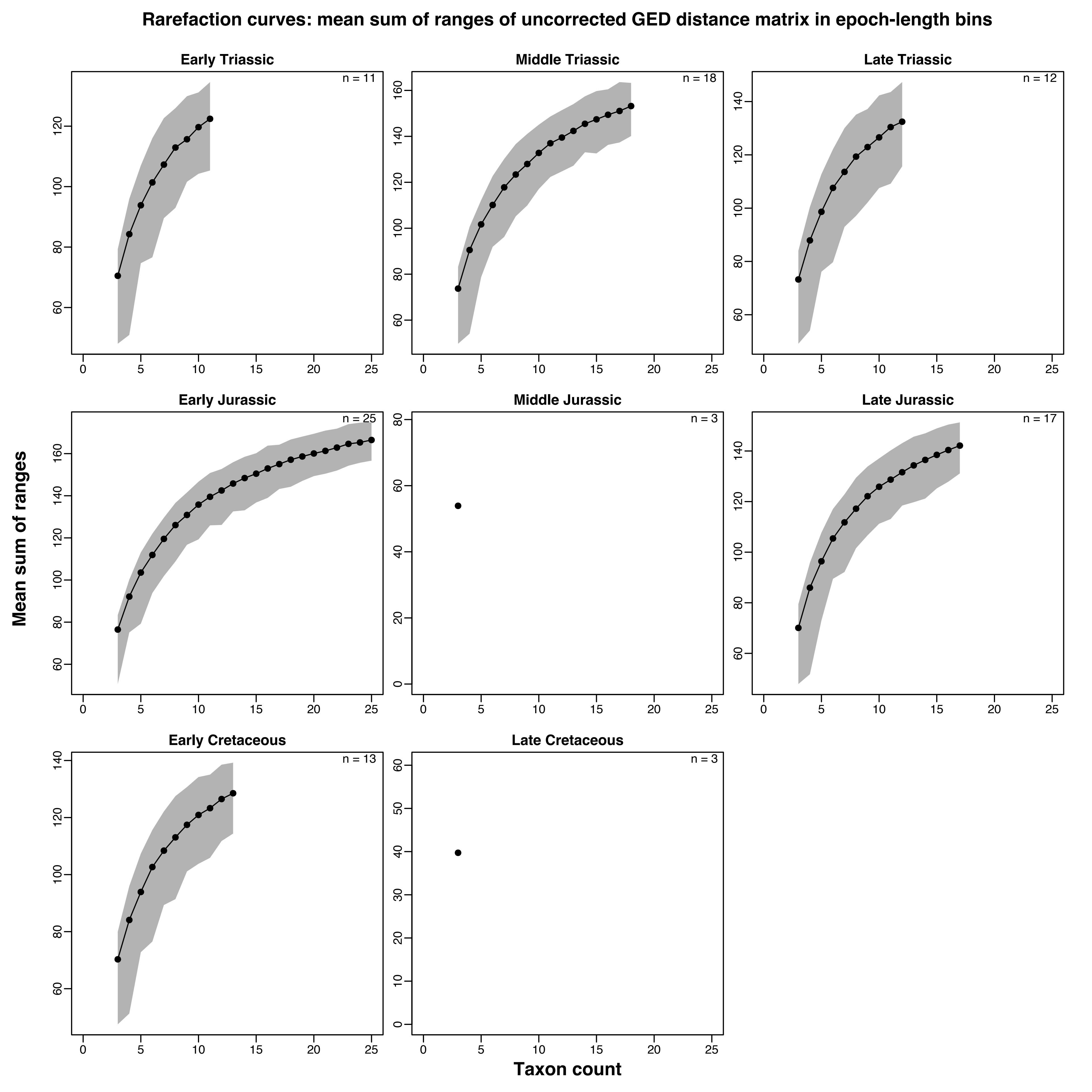

Rarefaction curves: mean sum of ranges of uncorrected GED distance matrix in epoch-length bins
Early Triassic
n = 11
Middle Triassic
n = 18
Late Triassic
n = 12
Early Jurassic
n = 25
Middle Jurassic
n = 3
Late Jurassic
n = 17
Early Cretaceous
n = 13
Late Cretaceous
n = 3
Mean sum of ranges
Taxon count

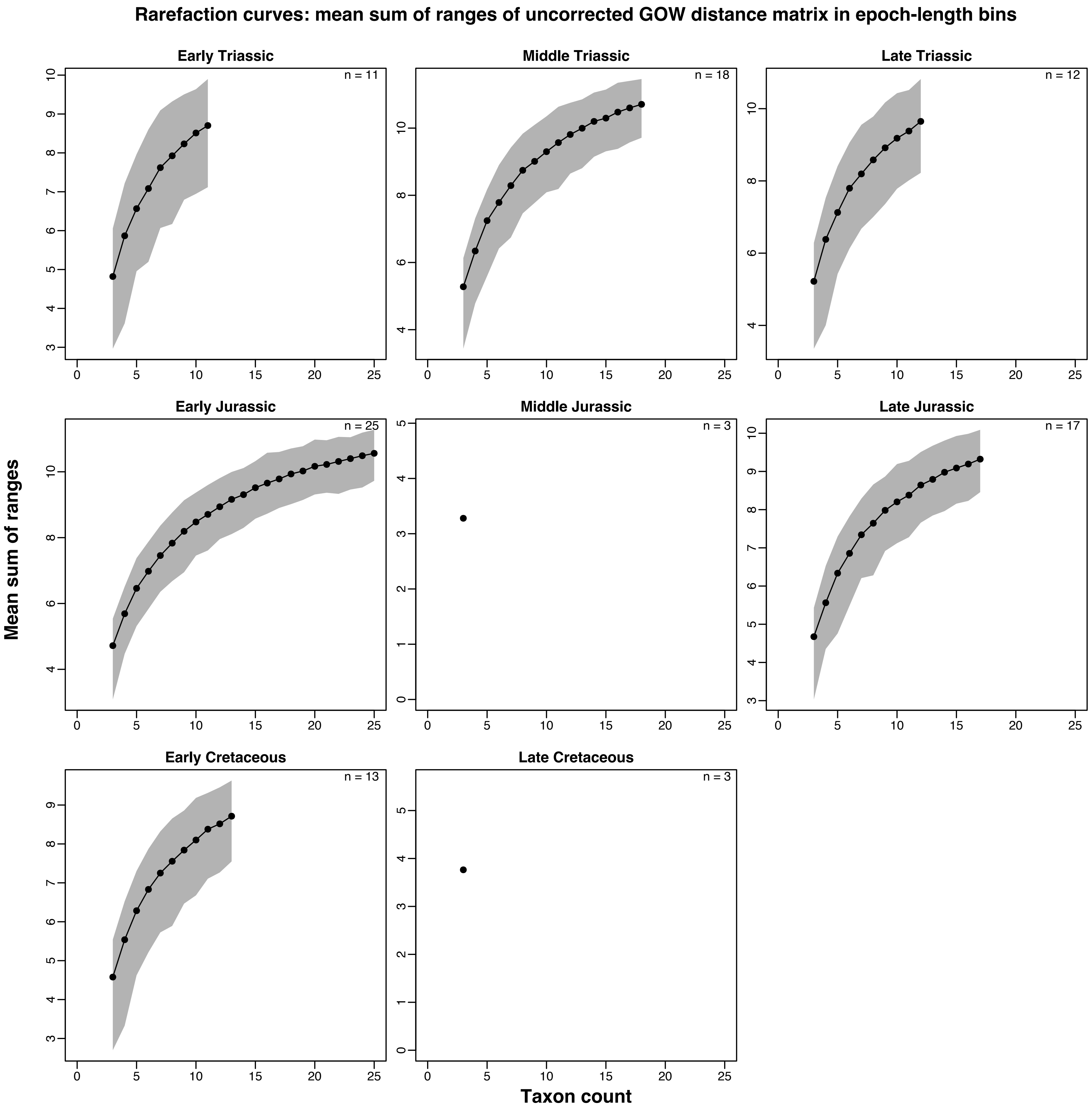
Rarefaction curves: mean sum of ranges of uncorrected GOW distance matrix in epoch-length bins
Early Triassic
n = 11
Middle Triassic
n = 18
Late Triassic
n = 12
Early Jurassic
n = 25
Middle Jurassic
n = 3
Late Jurassic
n = 17
Early Cretaceous
n = 13
Late Cretaceous
n = 3
Mean sum of ranges
Taxon count

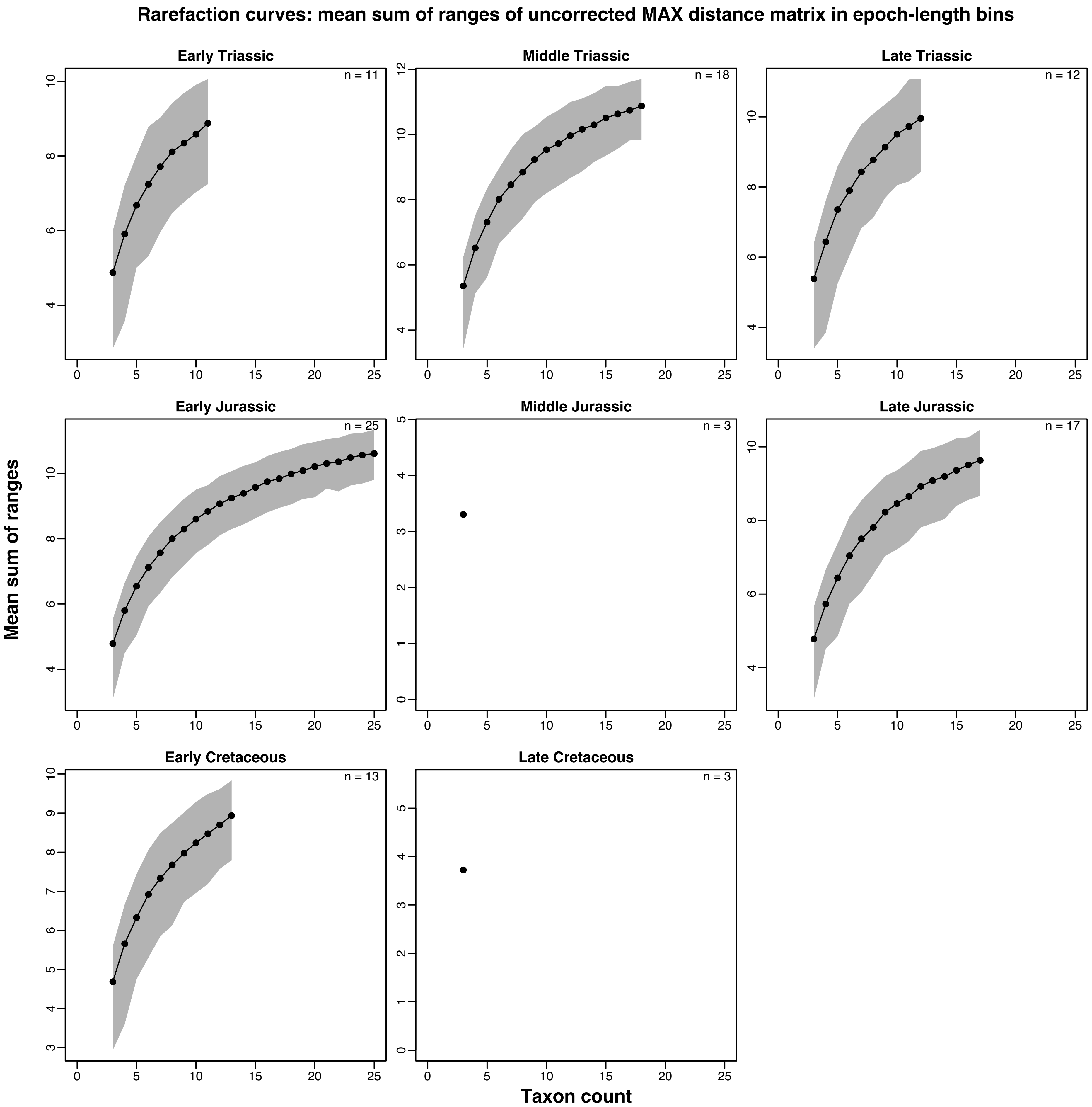

Rarefaction curves: mean sum of ranges of uncorrected MAX distance matrix in epoch-length bins
Early Triassic
n = 11
Middle Triassic
n = 18
Late Triassic
n = 12
Early Jurassic
n = 25
Middle Jurassic
n = 3
Late Jurassic
n = 17
Early Cretaceous
n = 13
Late Cretaceous
n = 3
Mean sum of ranges
Taxon count

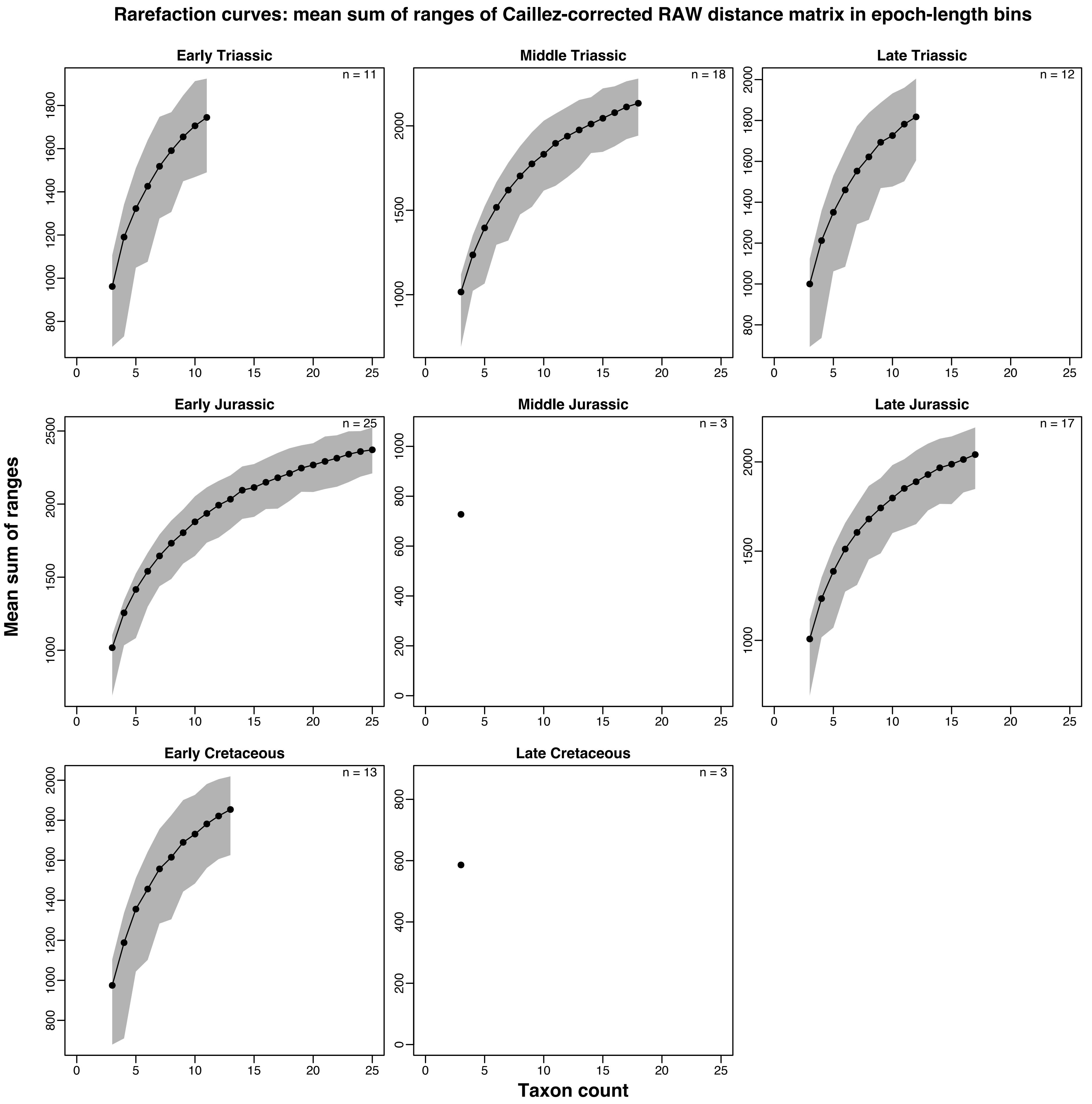
Rarefaction curves: mean sum of ranges of Caillez-corrected RAW distance matrix in epoch-length bins
Early Triassic
n = 11
Middle Triassic
n = 18
Late Triassic
n = 12
Early Jurassic
n = 25
Middle Jurassic
n = 3
Late Jurassic
n = 17
Early Cretaceous
n = 13
Late Cretaceous
n = 3
Mean sum of ranges
Taxon count

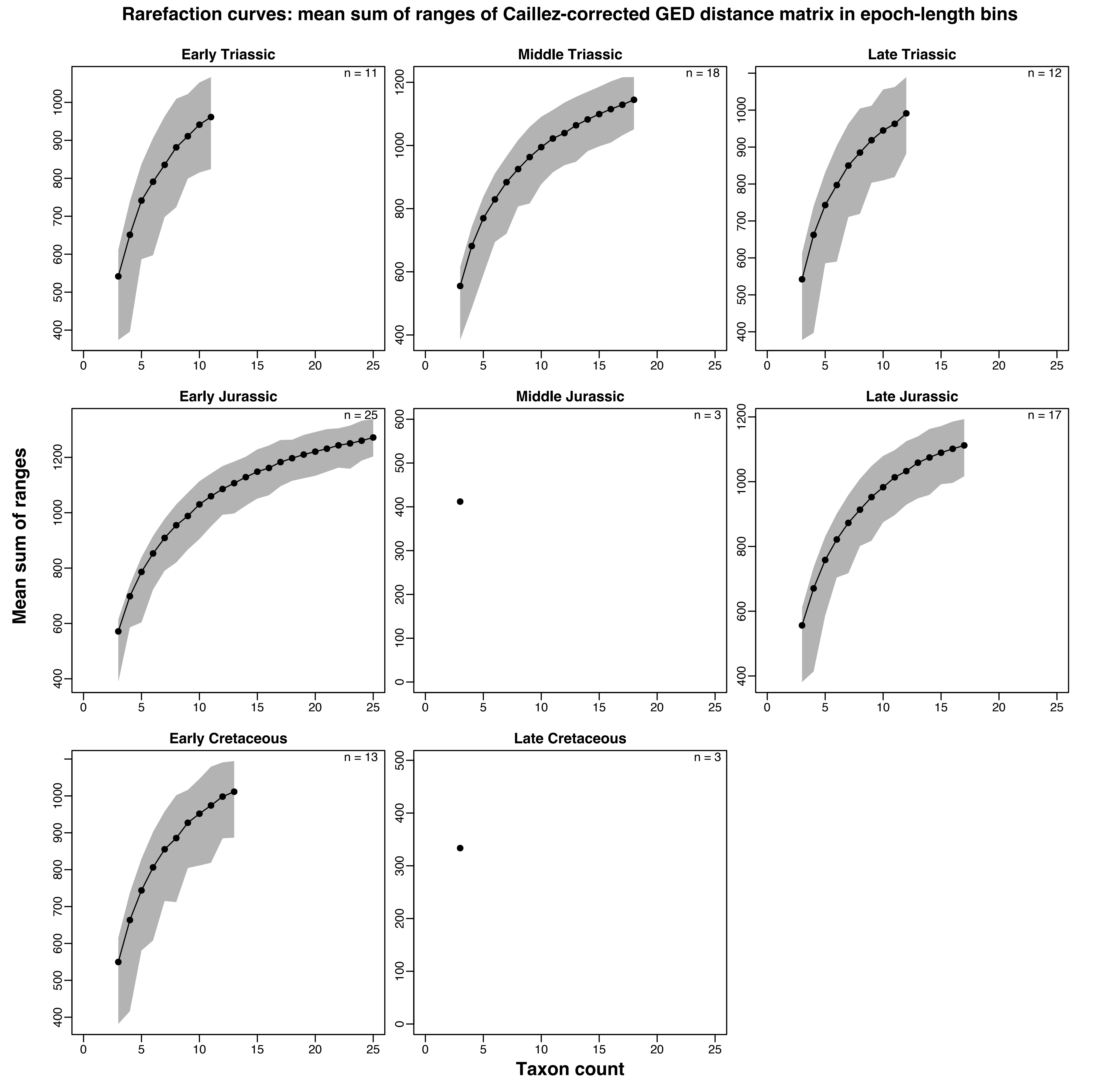
Rarefaction curves: mean sum of ranges of Caillez-corrected GED distance matrix in epoch-length bins
Early Triassic
n = 11
Middle Triassic
n = 18
Late Triassic
n = 12
Early Jurassic
n = 25
Middle Jurassic
n = 3
Late Jurassic
n = 17
Early Cretaceous
n = 13
Late Cretaceous
n = 3
Mean sum of ranges
Taxon count

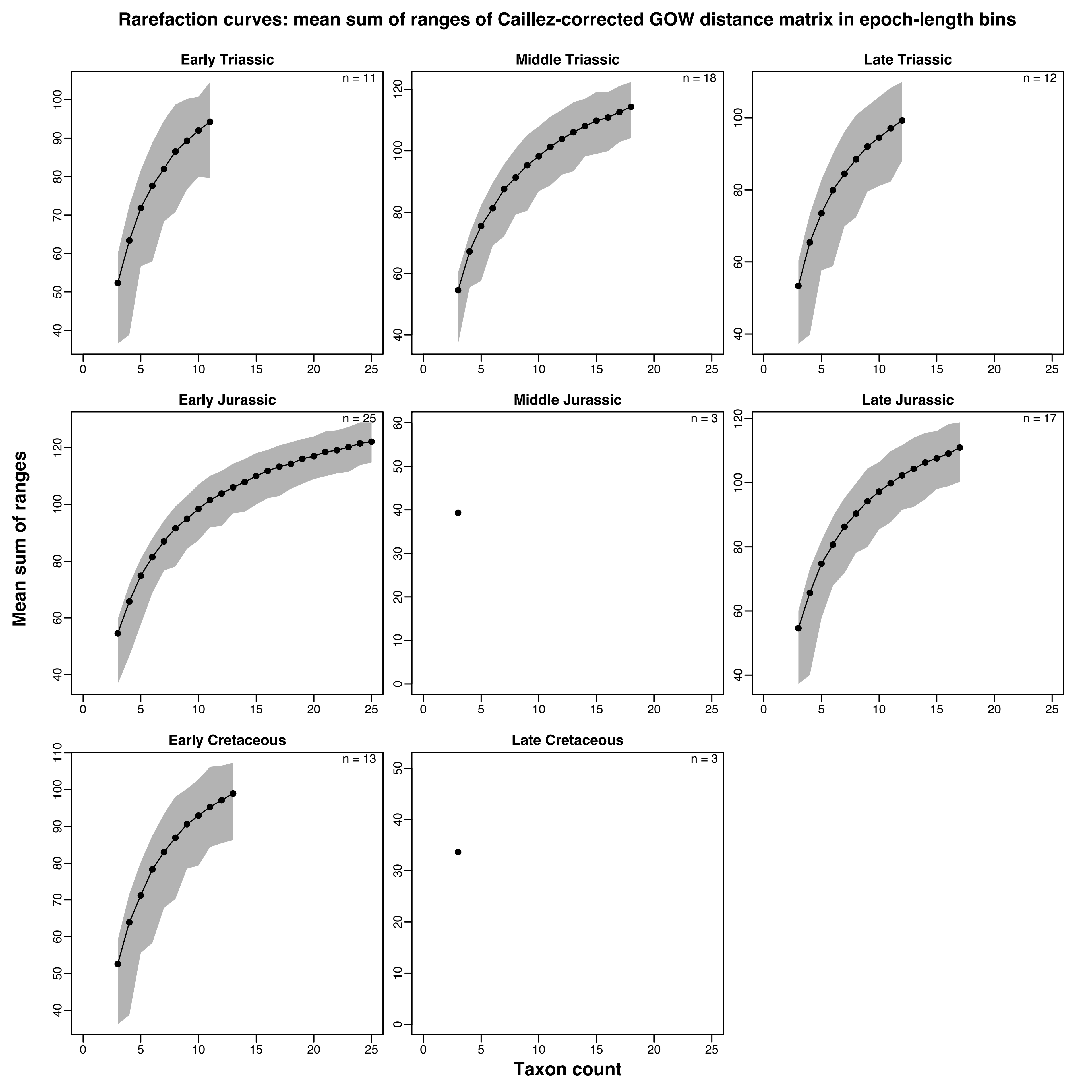

Rarefaction curves: mean sum of ranges of Caillez-corrected GOW distance matrix in epoch-length bins
Early Triassic
n = 11
Middle Triassic
n = 18
Late Triassic
n = 12
Early Jurassic
n = 25
Middle Jurassic
n = 3
Late Jurassic
n = 17
Early Cretaceous
n = 13
Late Cretaceous
n = 3
Mean sum of ranges
Taxon count

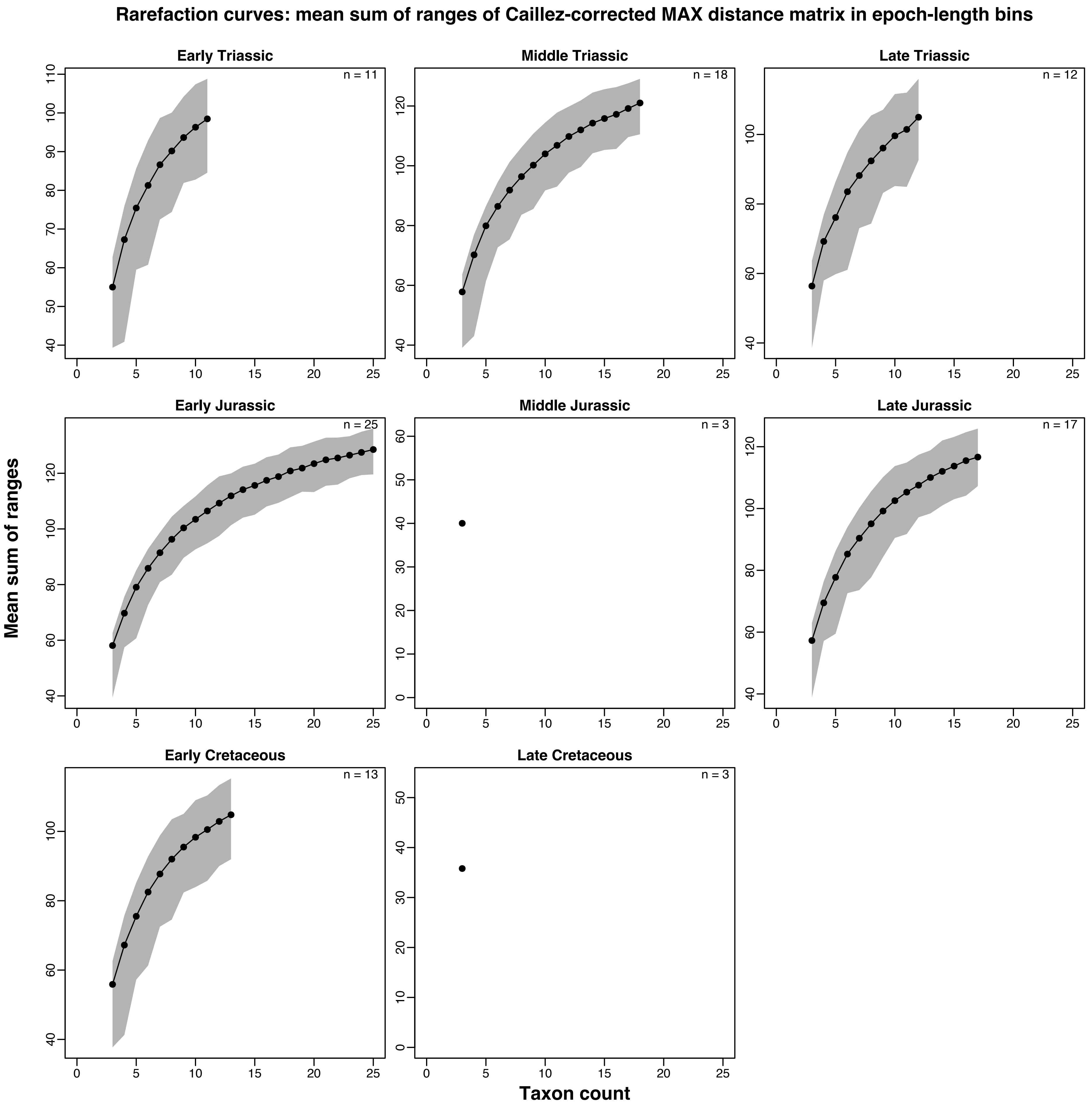
Rarefaction curves: mean sum of ranges of Caillez-corrected MAX distance matrix in epoch-length bins
Early Triassic
n = 11
Middle Triassic
n = 18
Late Triassic
n = 12
Early Jurassic
n = 25
Middle Jurassic
n = 3
Late Jurassic
n = 17
Early Cretaceous
n = 13
Late Cretaceous
n = 3
Mean sum of ranges
Taxon count

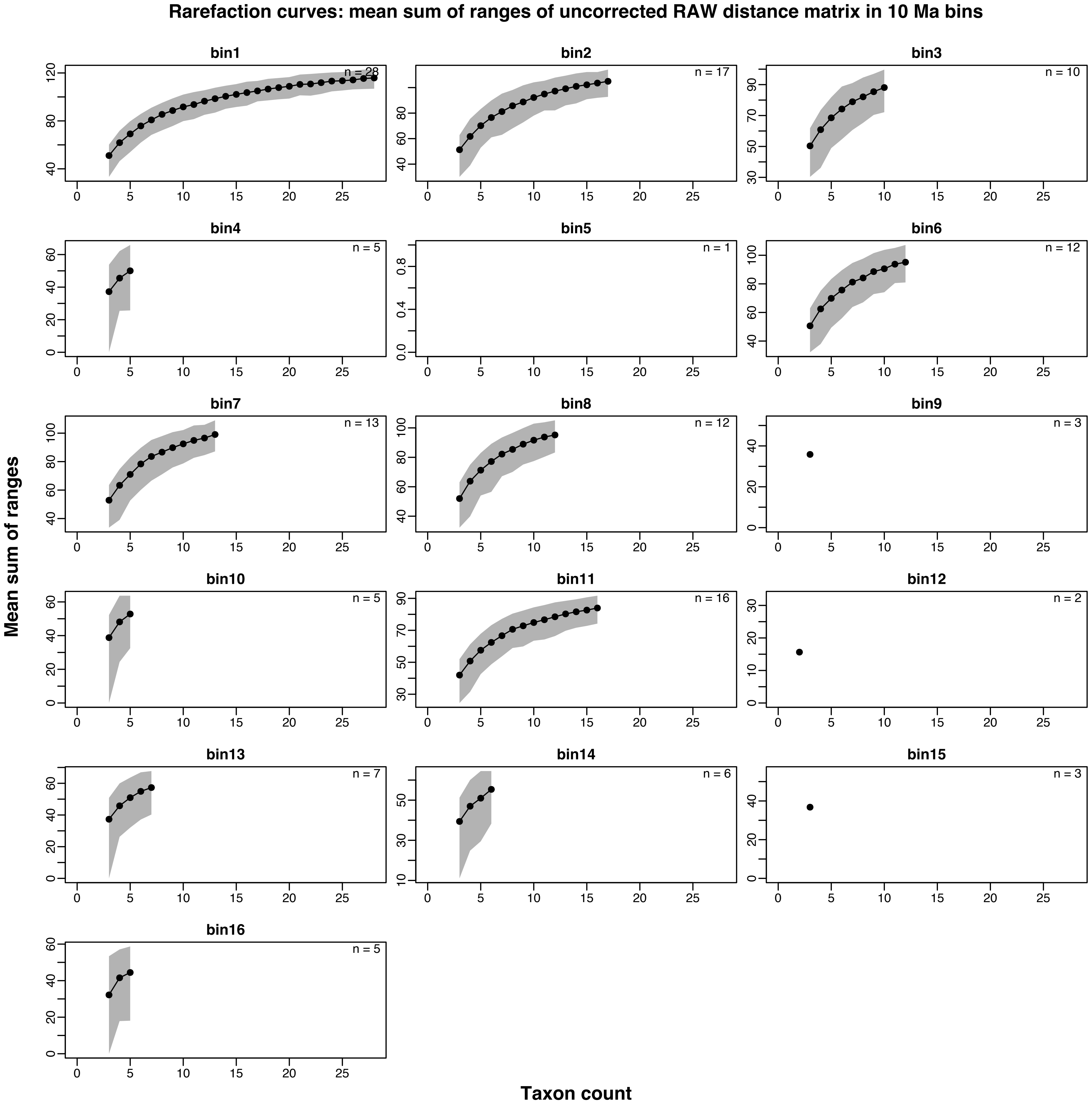
Rarefaction curves: mean sum of ranges of uncorrected RAW distance matrix in 10 Ma bins
bin1
n = 28
bin2
n = 17
bin3
n = 10
bin4
n = 5
bin5
n = 1
bin6
n = 12
bin7
n = 13
bin8
n = 12
bin9
n = 3
bin10
n = 5
bin11
n = 16
bin12
n = 2
bin13
n = 7
bin14
n = 6
bin15
n = 3
bin16
n = 5
Mean sum of ranges
Taxon count

Rarefaction curves: mean sum of ranges of uncorrected GED distance matrix in 10 Ma bins

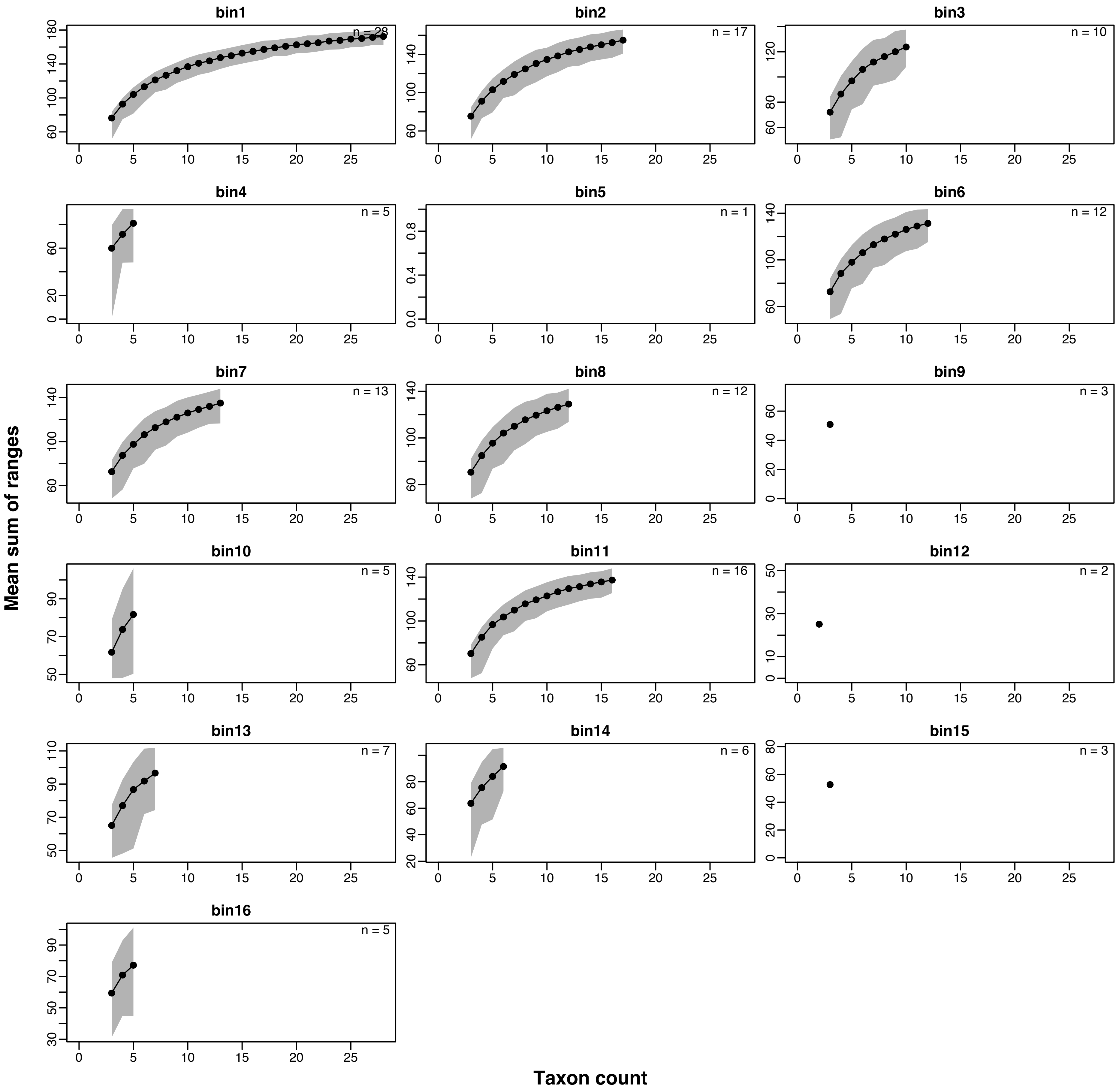

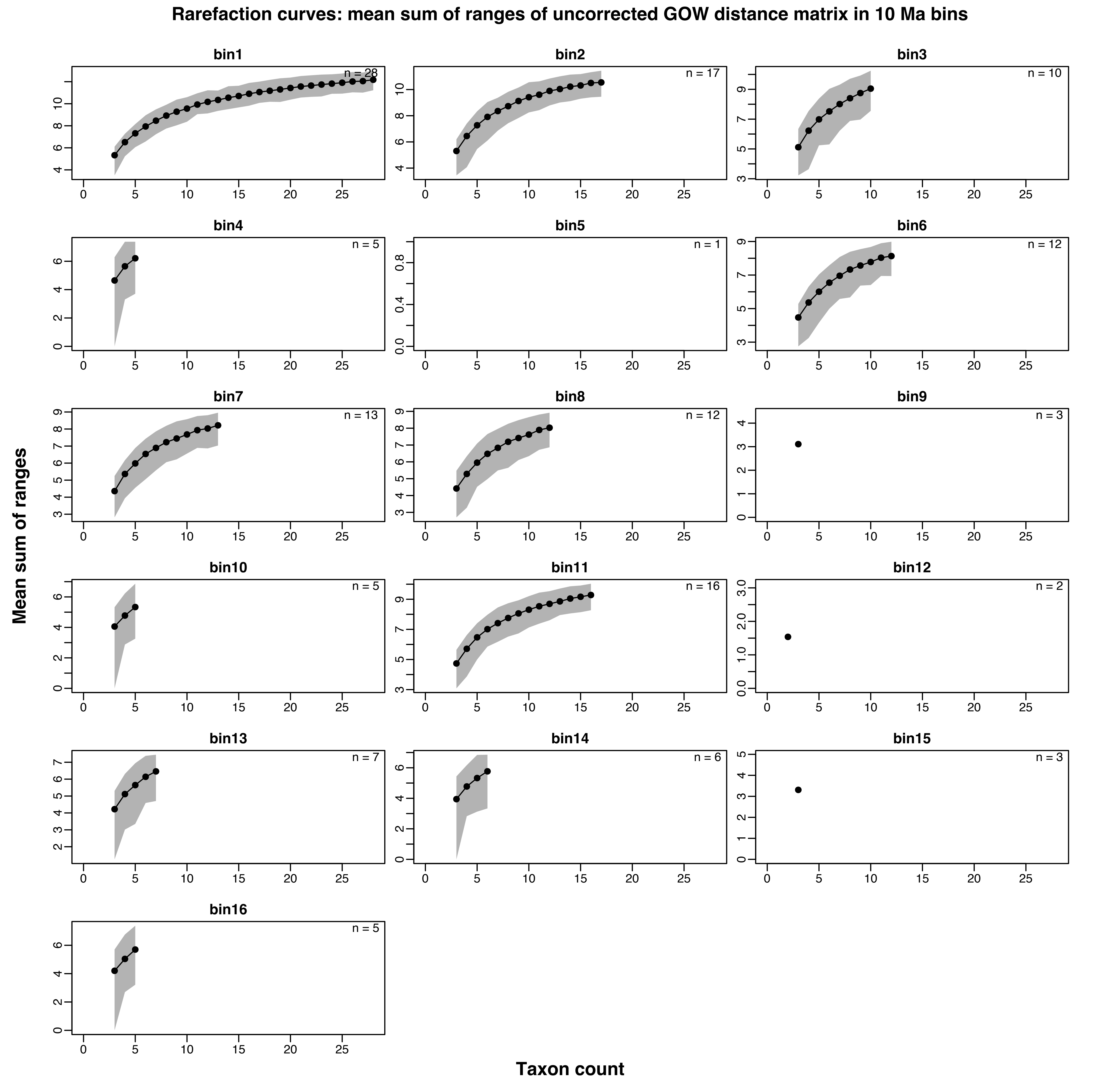
Rarefaction curves: mean sum of ranges of uncorrected GOW distance matrix in 10 Ma bins
bin1
n = 28
bin2
n = 17
bin3
n = 10
bin4
n = 5
bin5
n = 1
bin6
n = 12
bin7
n = 13
bin8
n = 12
bin9
n = 3
bin10
n = 5
bin11
n = 16
bin12
n = 2
bin13
n = 7
bin14
n = 6
bin15
n = 3
bin16
n = 5
Mean sum of ranges
Taxon count

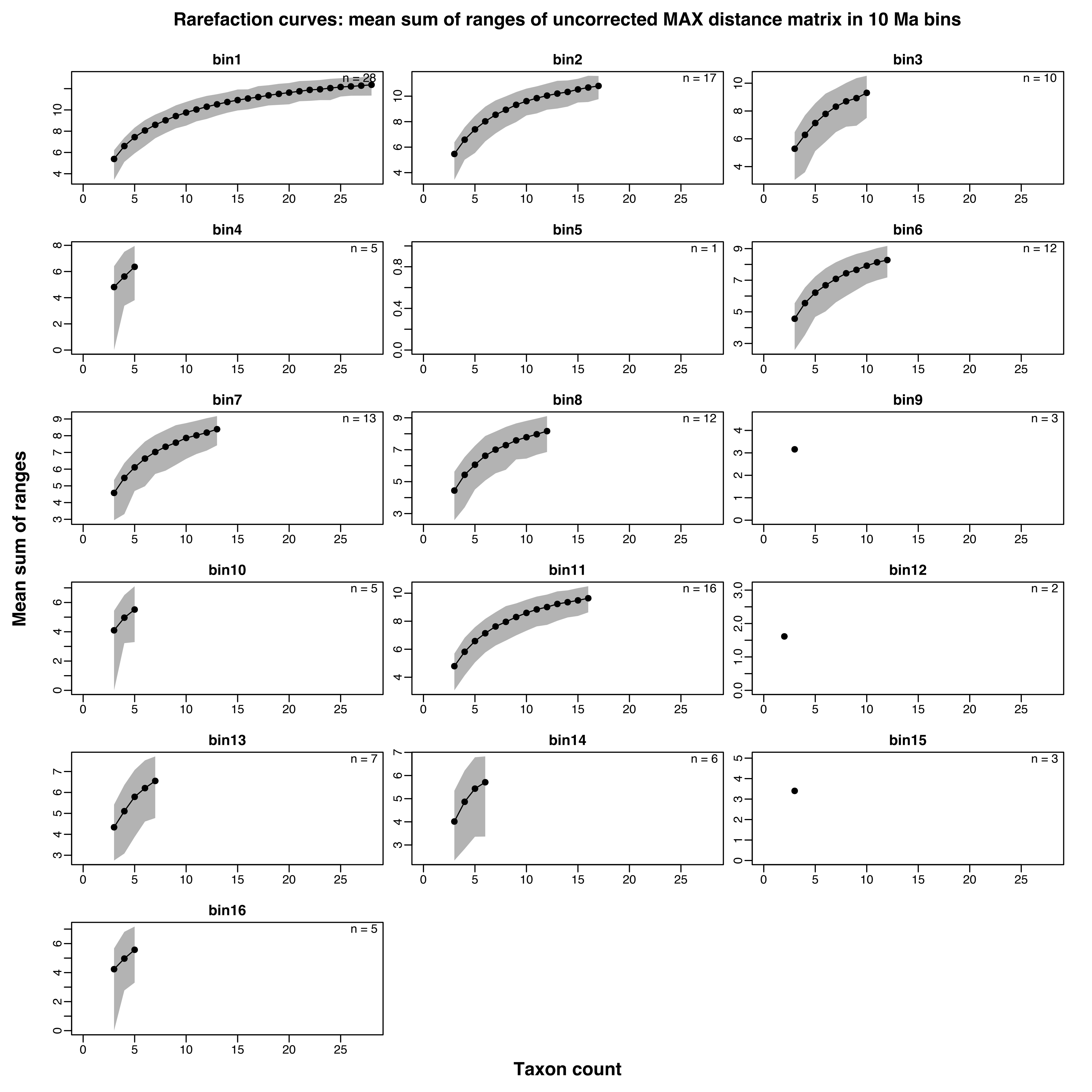
Rarefaction curves: mean sum of ranges of uncorrected MAX distance matrix in 10 Ma bins
bin1
n = 28
bin2
n = 17
bin3
n = 10
bin4
n = 5
bin5
n = 1
bin6
n = 12
bin7
n = 13
bin8
n = 12
bin9
n = 3
bin10
n = 5
bin11
n = 16
bin12
n = 2
bin13
n = 7
bin14
n = 6
bin15
n = 3
bin16
n = 5
Mean sum of ranges
Taxon count

Rarefaction curves: mean sum of ranges of Caillez-corrected RAW distance matrix in 10 Ma bins

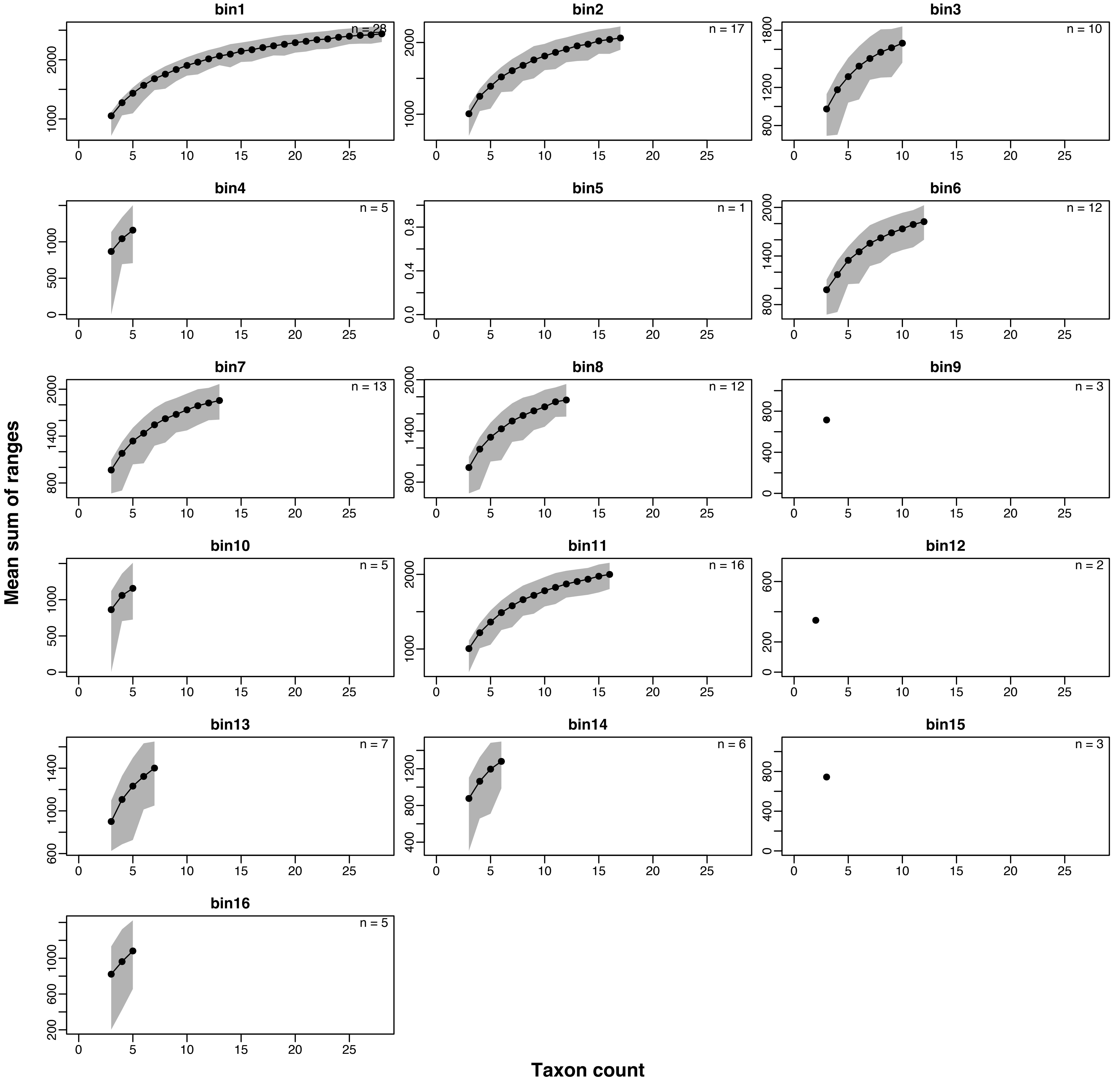

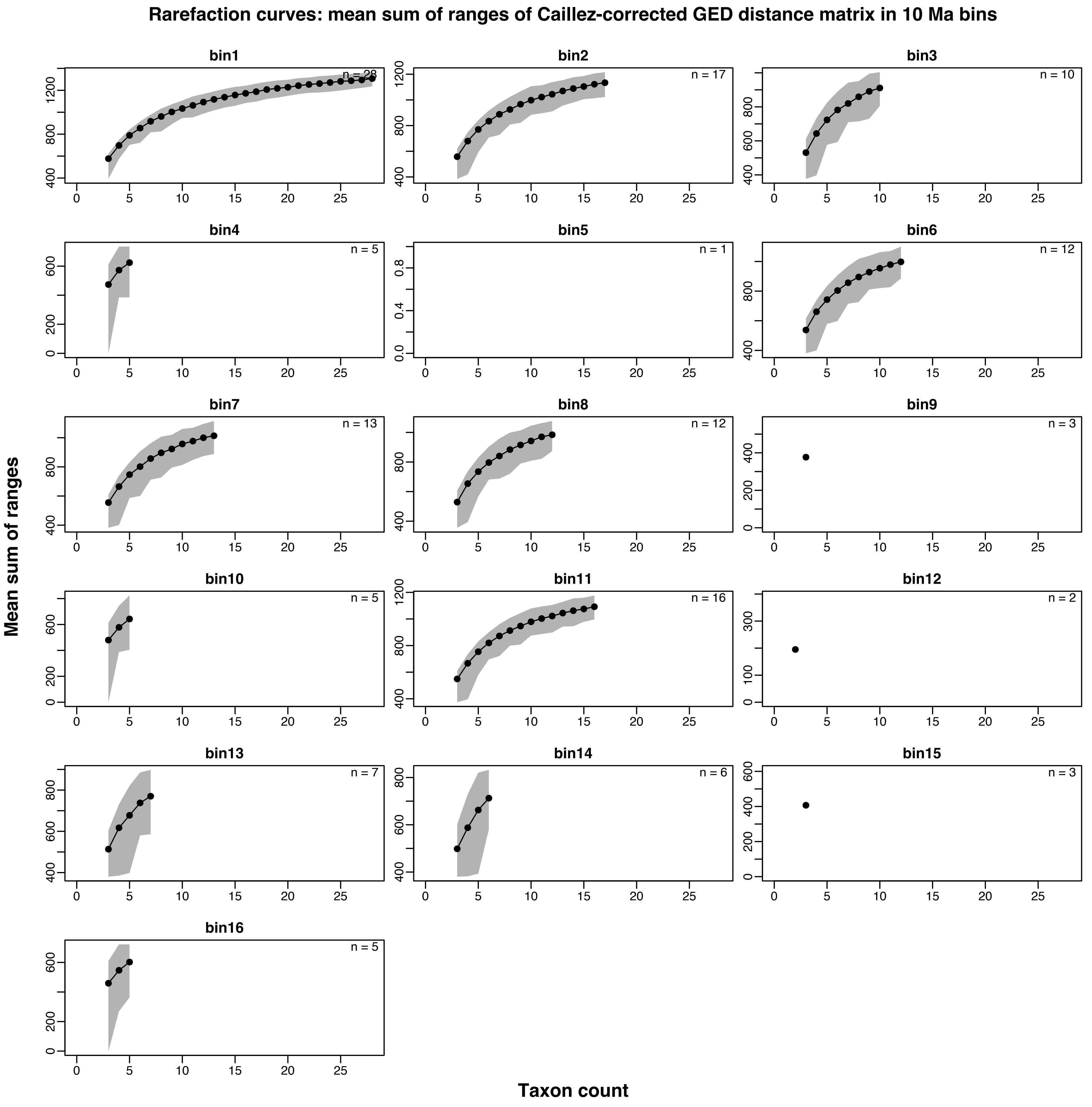
Rarefaction curves: mean sum of ranges of Caillez-corrected GED distance matrix in 10 Ma bins
bin1
n = 28
bin2
n = 17
bin3
n = 10
bin4
n = 5
bin5
n = 1
bin6
n = 12
bin7
n = 13
bin8
n = 12
bin9
n = 3
bin10
n = 5
bin11
n = 16
bin12
n = 2
bin13
n = 7
bin14
n = 6
bin15
n = 3
bin16
n = 5
Mean sum of ranges
Taxon count

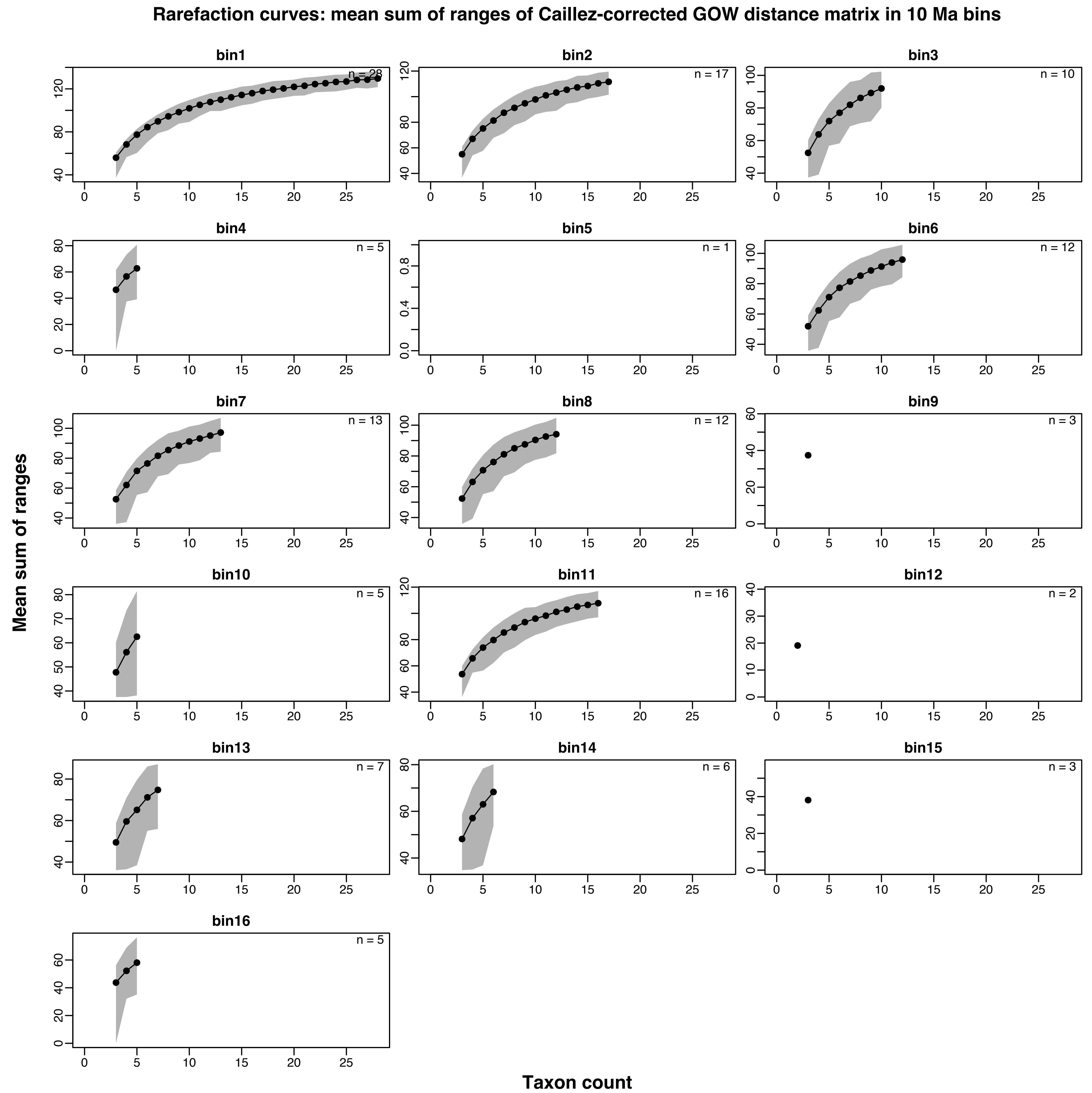

Rarefaction curves: mean sum of ranges of Caillez-corrected GOW distance matrix in 10 Ma bins
bin1
n = 28
bin2
n = 17
bin3
n = 10
bin4
n = 5
bin5
n = 1
bin6
n = 12
bin7
n = 13
bin8
n = 12
bin9
n = 3
bin10
n = 5
bin11
n = 16
bin12
n = 2
bin13
n = 7
bin14
n = 6
bin15
n = 3
bin16
n = 5
Mean sum of ranges
Taxon count

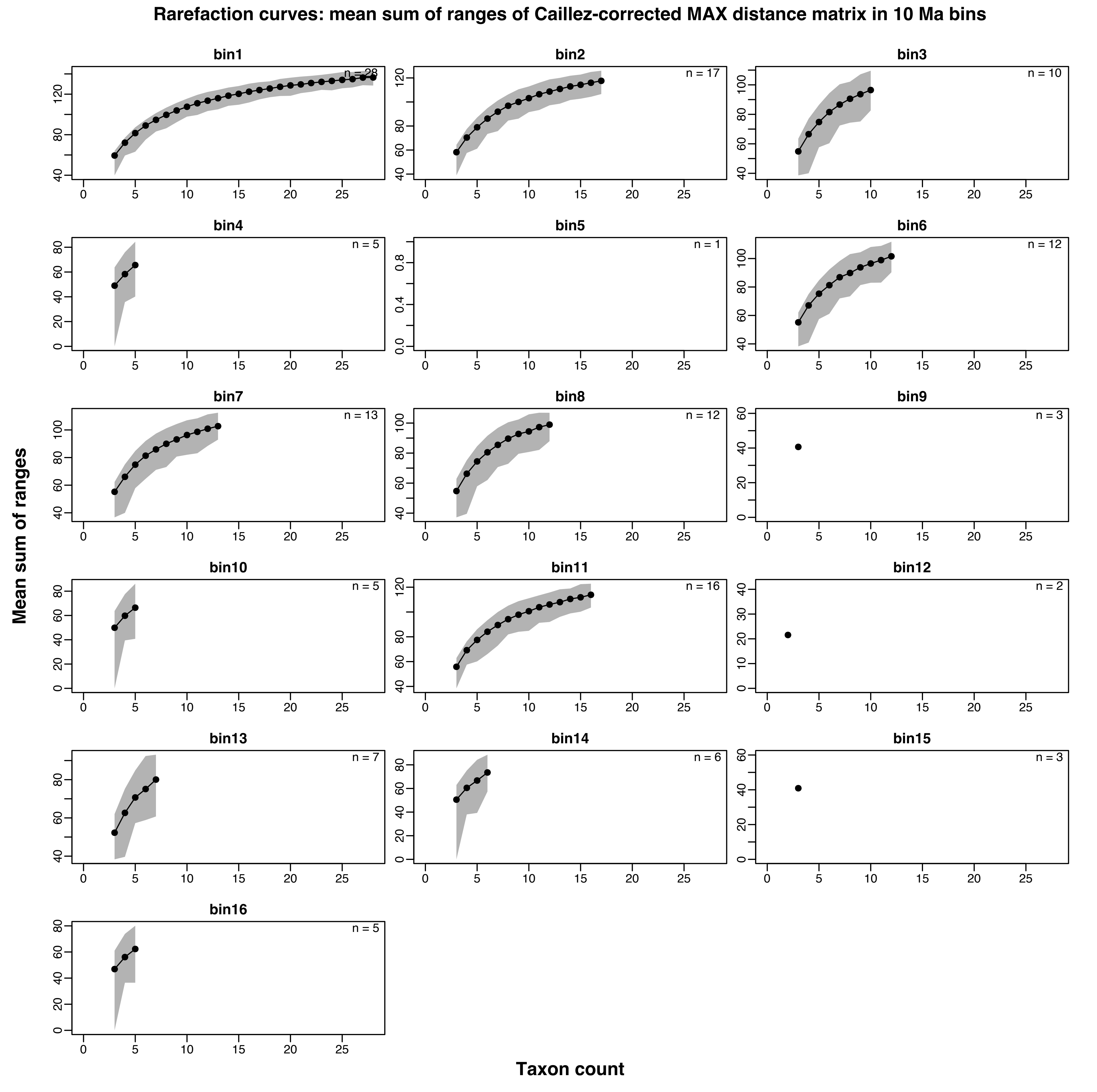
Rarefaction curves: mean sum of ranges of Caillez-corrected MAX distance matrix in 10 Ma bins
bin1
n = 28
bin2
n = 17
bin3
n = 10
bin4
n = 5
bin5
n = 1
bin6
n = 12
bin7
n = 13
bin8
n = 12
bin9
n = 3
bin10
n = 5
bin11
n = 16
bin12
n = 2
bin13
n = 7
bin14
n = 6
bin15
n = 3
bin16
n = 5
Mean sum of ranges
Taxon count

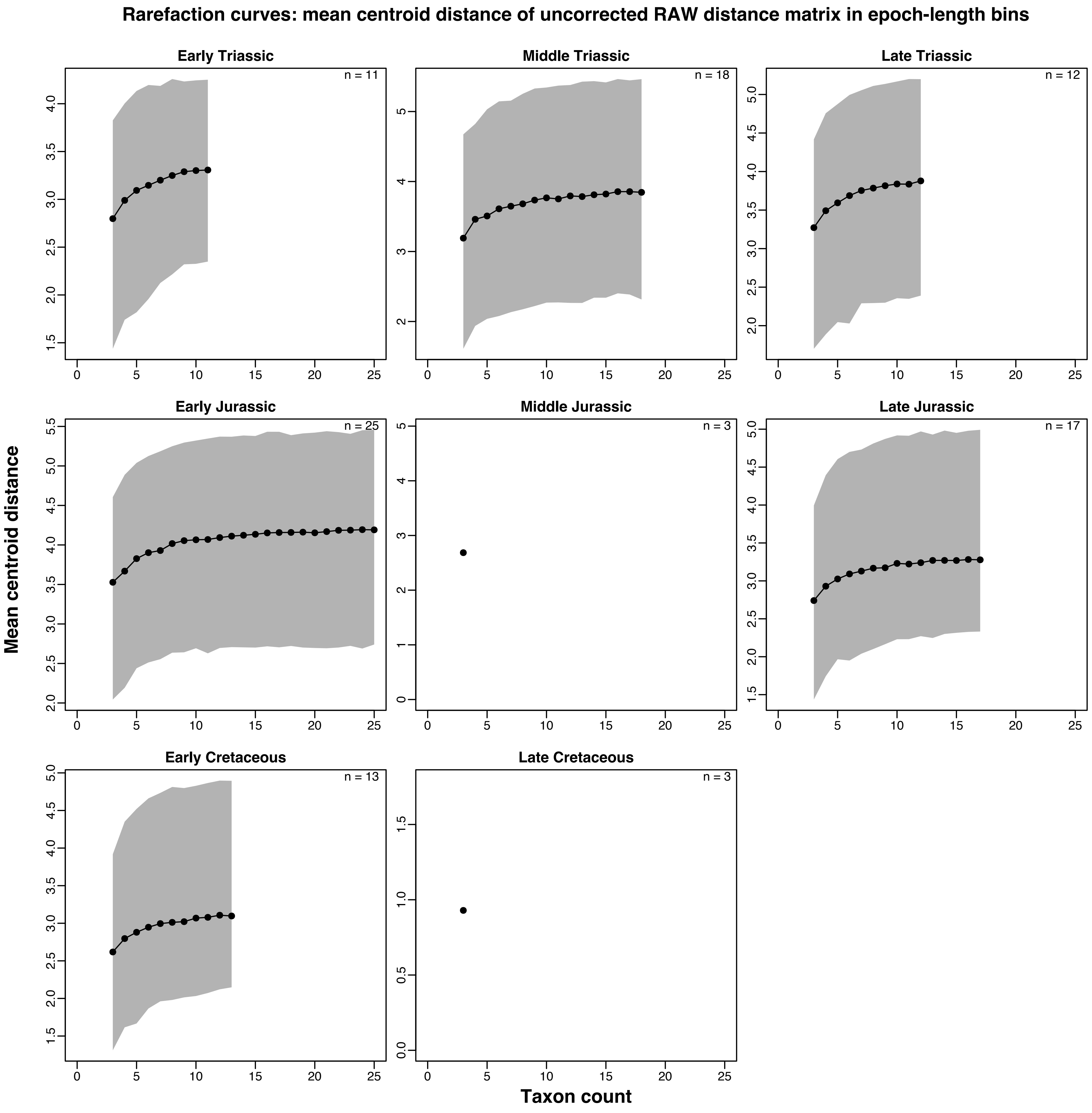

Rarefaction curves: mean centroid distance of uncorrected RAW distance matrix in epoch-length bins
Early Triassic
n = 11
Middle Triassic
n = 18
Late Triassic
n = 12
Early Jurassic
n = 25
Middle Jurassic
n = 3
Late Jurassic
n = 17
Early Cretaceous
n = 13
Late Cretaceous
n = 3
Mean centroid distance
Taxon count

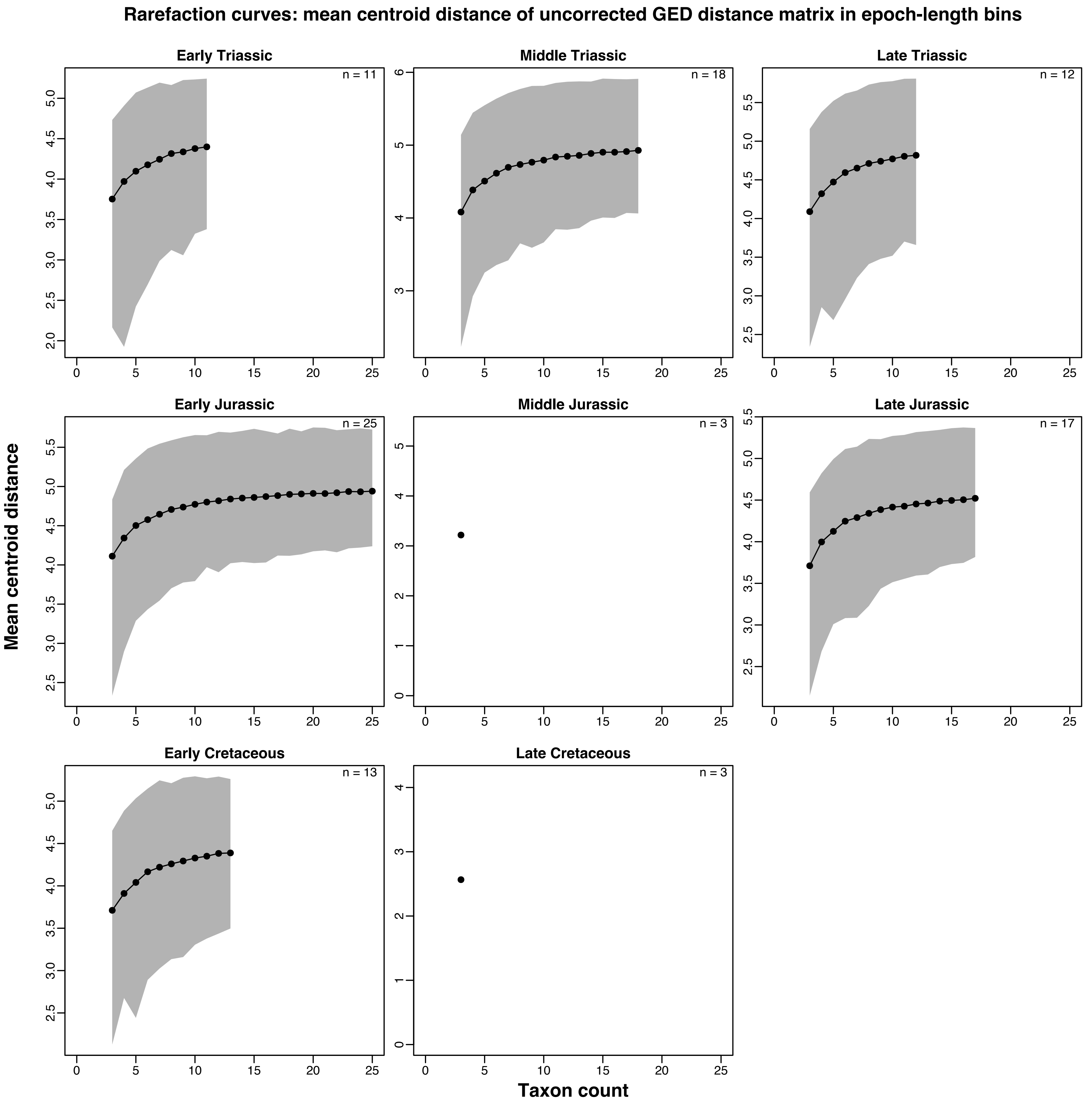

Rarefaction curves: mean centroid distance of uncorrected GED distance matrix in epoch-length bins
Early Triassic
n = 11
Middle Triassic
n = 18
Late Triassic
n = 12
Early Jurassic
n = 25
Middle Jurassic
n = 3
Late Jurassic
n = 17
Early Cretaceous
n = 13
Late Cretaceous
n = 3
Mean centroid distance
Taxon count

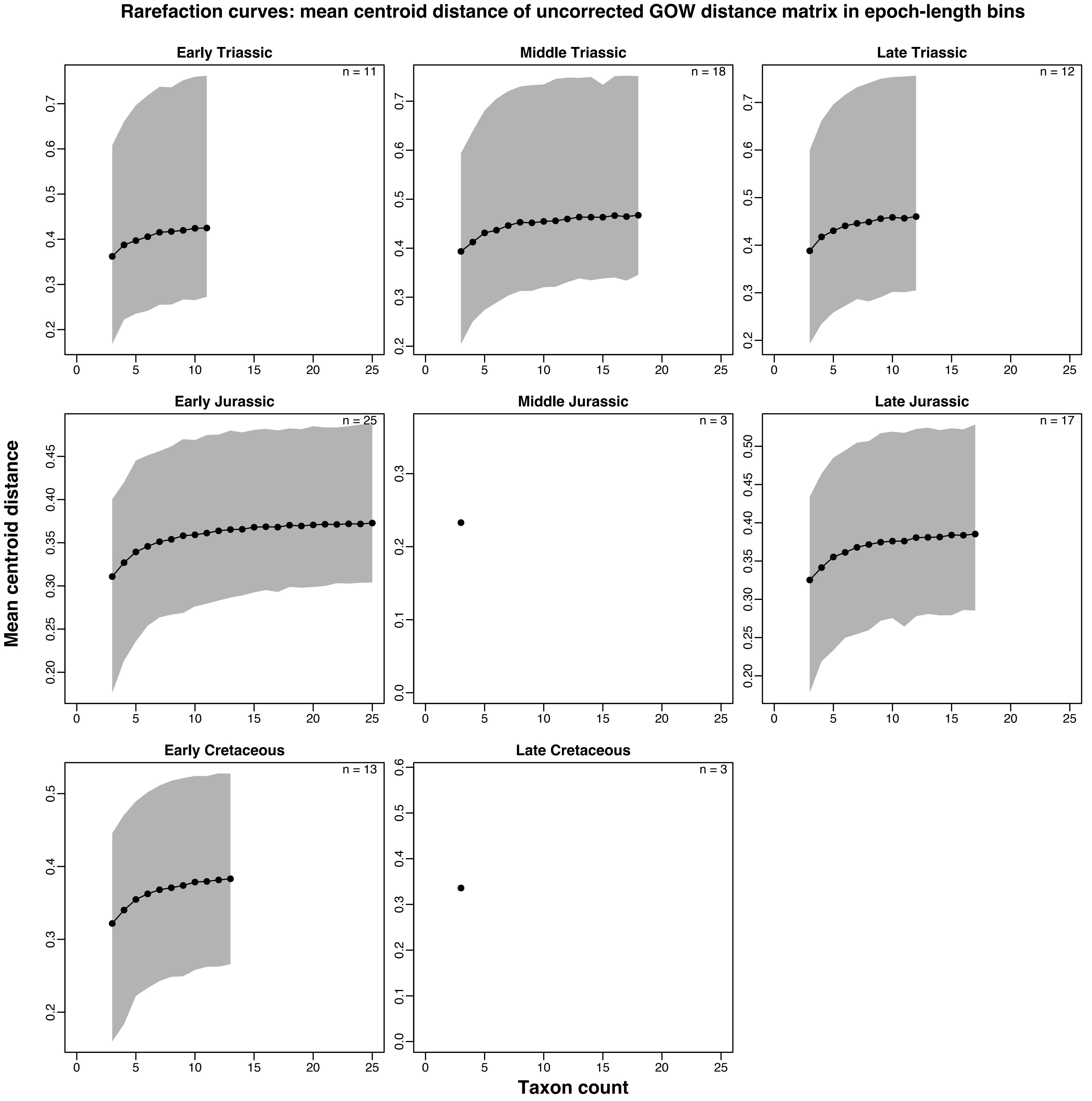
Rarefaction curves: mean centroid distance of uncorrected GOW distance matrix in epoch-length bins
Early Triassic
n = 11
Middle Triassic
n = 18
Late Triassic
n = 12
Early Jurassic
n = 25
Middle Jurassic
n = 3
Late Jurassic
n = 17
Early Cretaceous
n = 13
Late Cretaceous
n = 3
Mean centroid distance
Taxon count

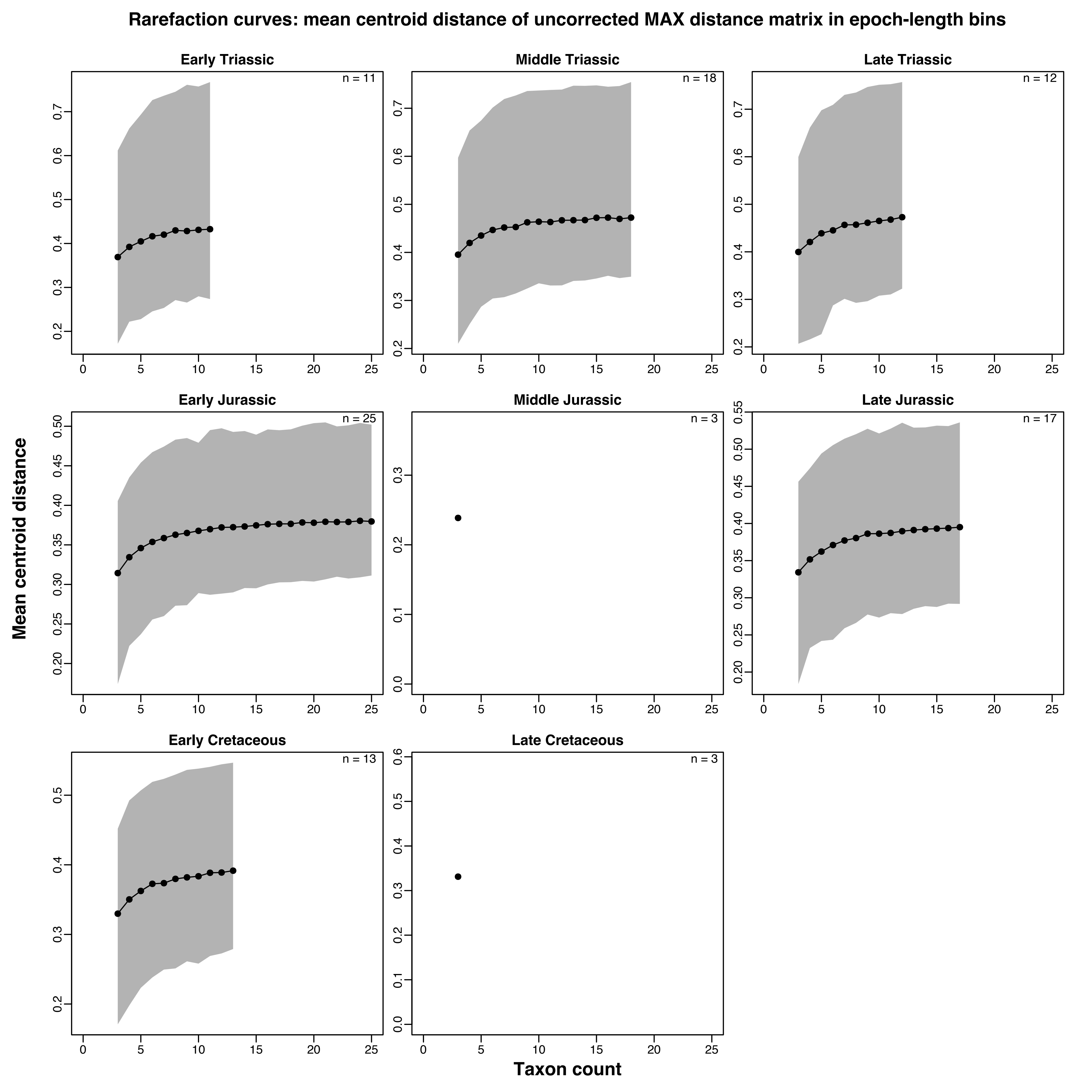

Rarefaction curves: mean centroid distance of uncorrected MAX distance matrix in epoch-length bins
Early Triassic
n = 11
Middle Triassic
n = 18
Late Triassic
n = 12
Early Jurassic
n = 25
Middle Jurassic
n = 3
Late Jurassic
n = 17
Early Cretaceous
n = 13
Late Cretaceous
n = 3
Mean centroid distance
Taxon count

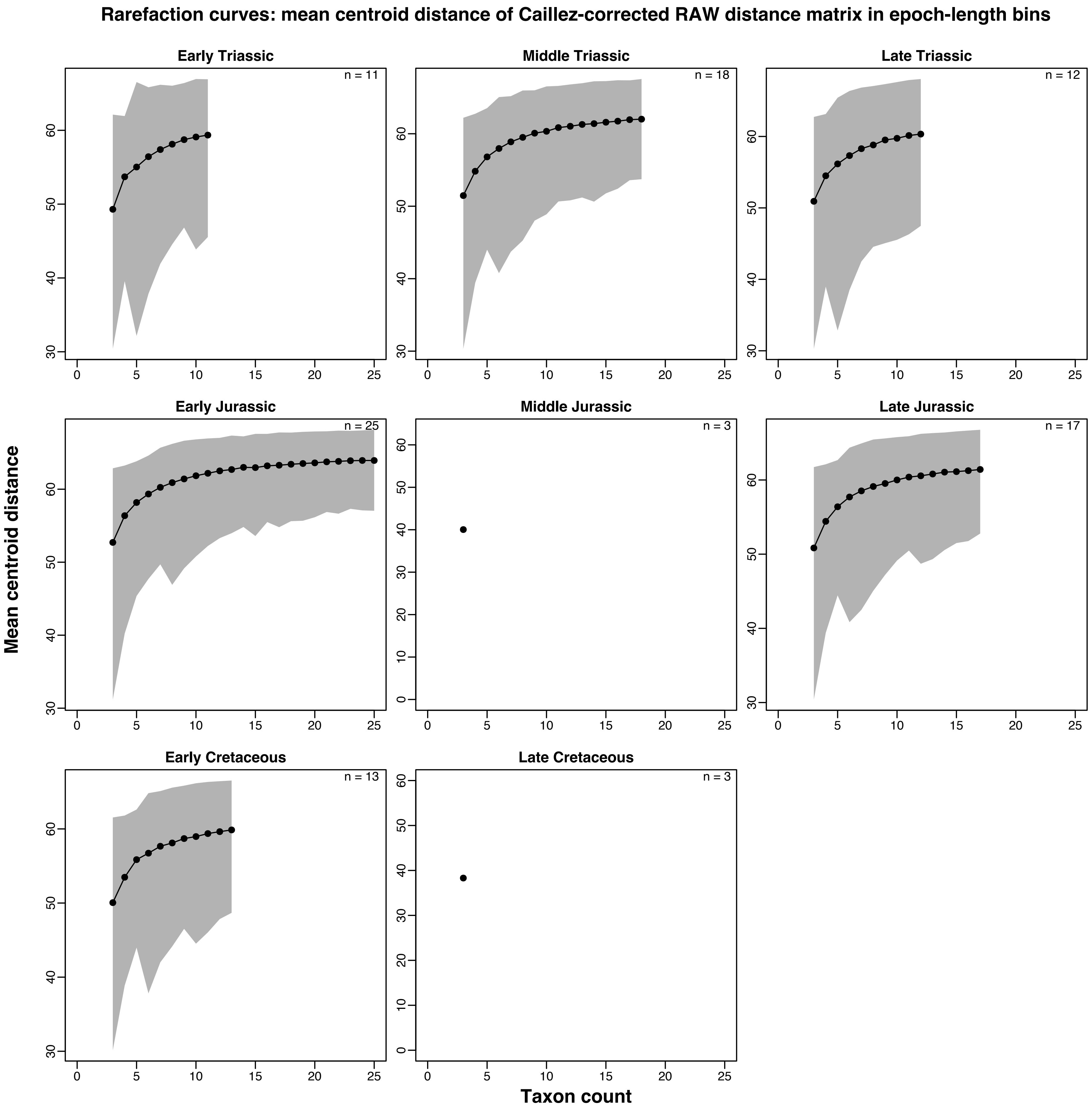
Rarefaction curves: mean centroid distance of Caillez-corrected RAW distance matrix in epoch-length bins
Early Triassic
n = 11
Middle Triassic
n = 18
Late Triassic
n = 12
Early Jurassic
n = 25
Middle Jurassic
n = 3
Late Jurassic
n = 17
Early Cretaceous
n = 13
Late Cretaceous
n = 3
Mean centroid distance
Taxon count

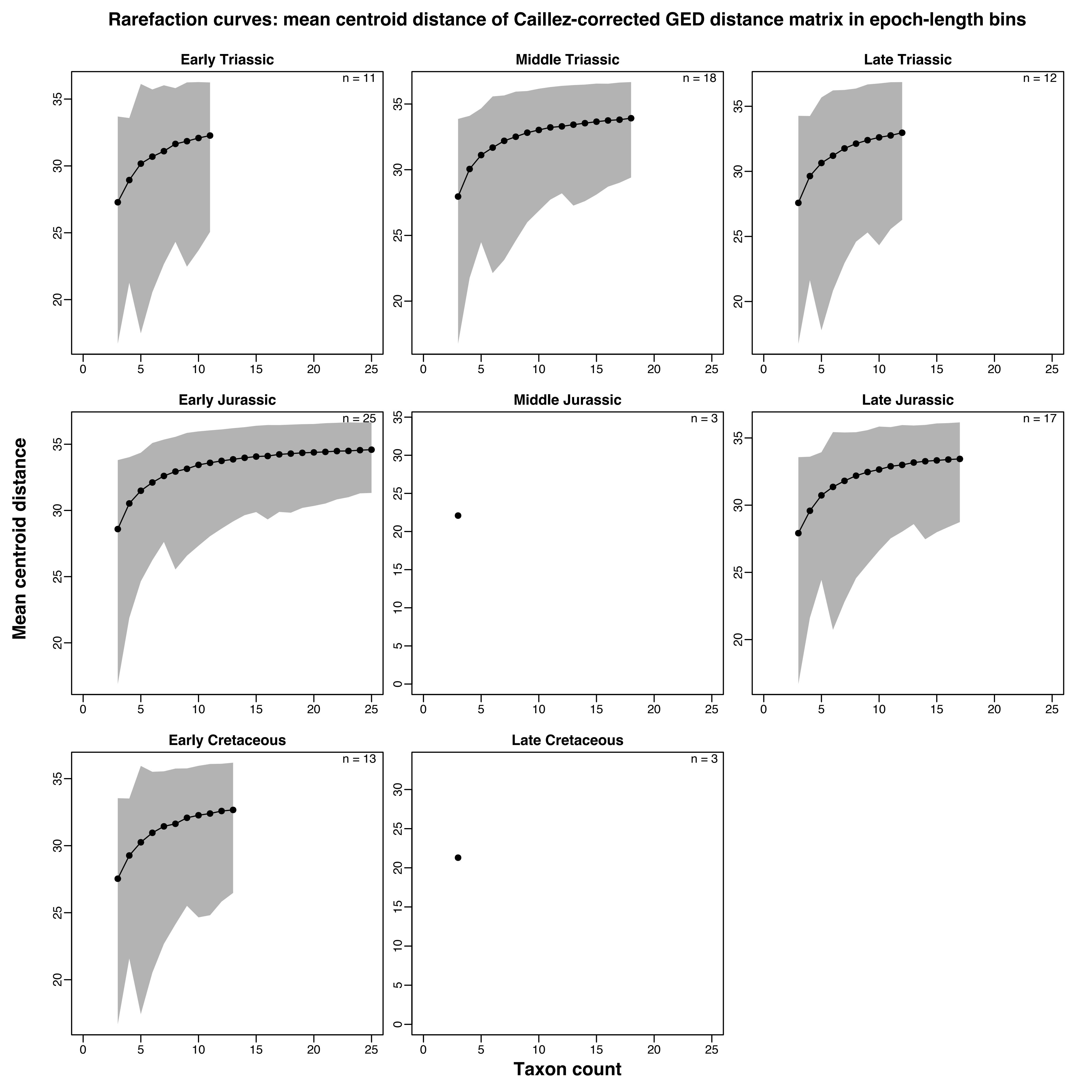

Rarefaction curves: mean centroid distance of Caillez-corrected GED distance matrix in epoch-length bins
Early Triassic
n = 11
Middle Triassic
n = 18
Late Triassic
n = 12
Early Jurassic
n = 25
Middle Jurassic
n = 3
Late Jurassic
n = 17
Early Cretaceous
n = 13
Late Cretaceous
n = 3
Mean centroid distance
Taxon count

Rarefaction curves: mean centroid distance of Caillez-corrected GOW distance matrix in epoch-length bins

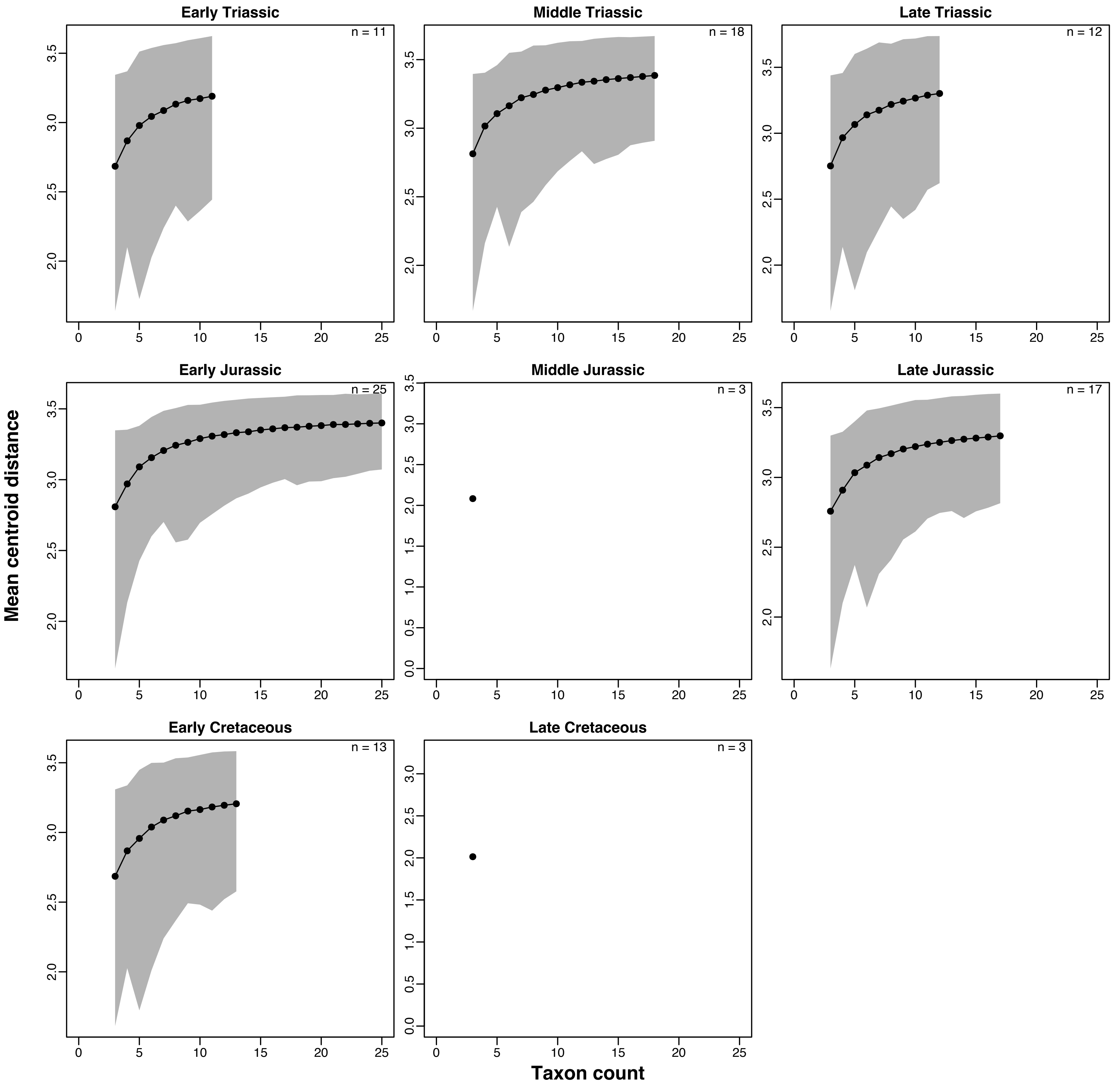

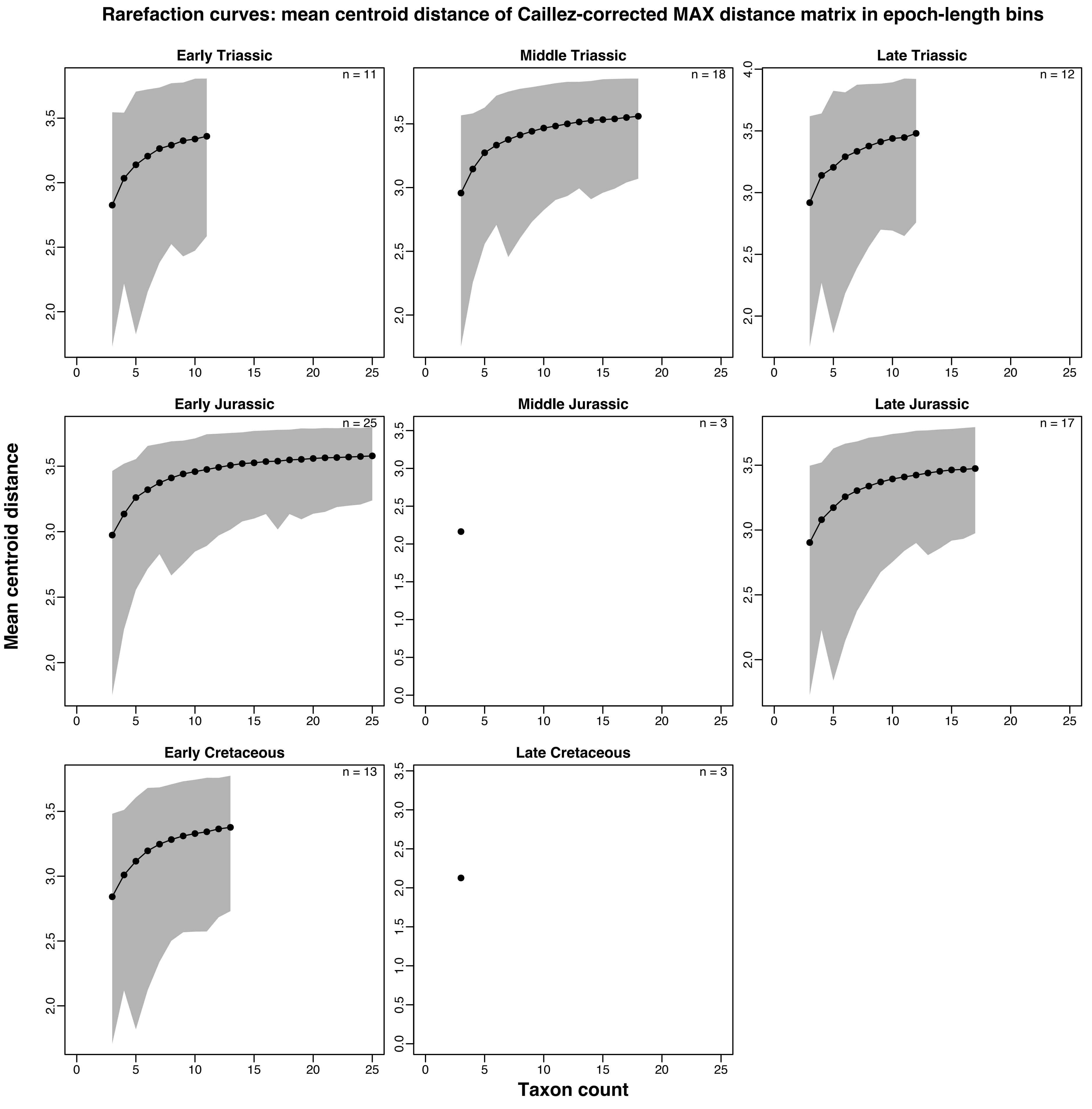
Rarefaction curves: mean centroid distance of Caillez-corrected MAX distance matrix in epoch-length bins
Early Triassic
n = 11
Middle Triassic
n = 18
Late Triassic
n = 12
Early Jurassic
n = 25
Middle Jurassic
n = 3
Late Jurassic
n = 17
Early Cretaceous
n = 13
Late Cretaceous
n = 3
Mean centroid distance
Taxon count

Rarefaction curves: mean centroid distance of uncorrected RAW distance matrix in 10 Ma bins

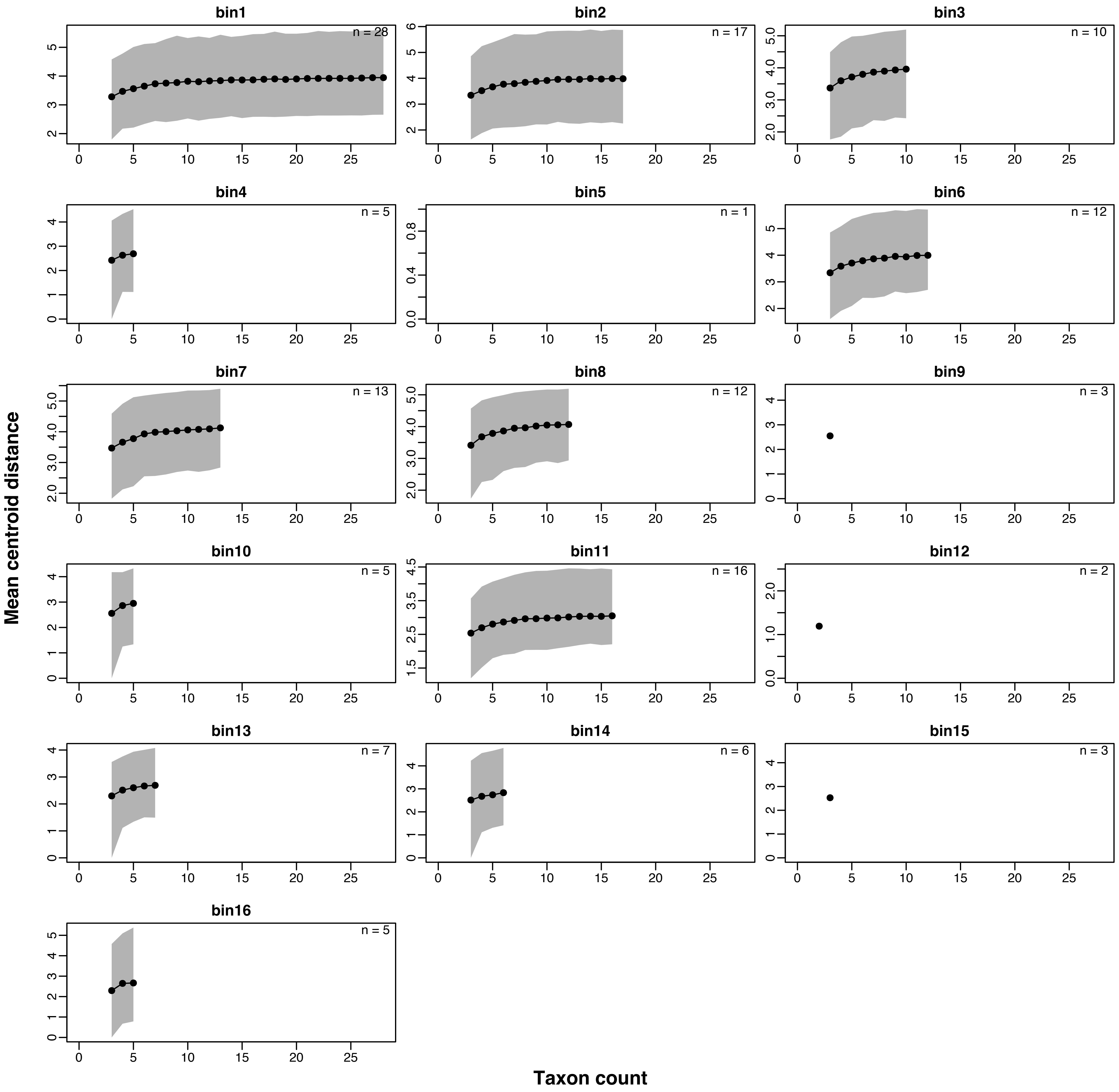

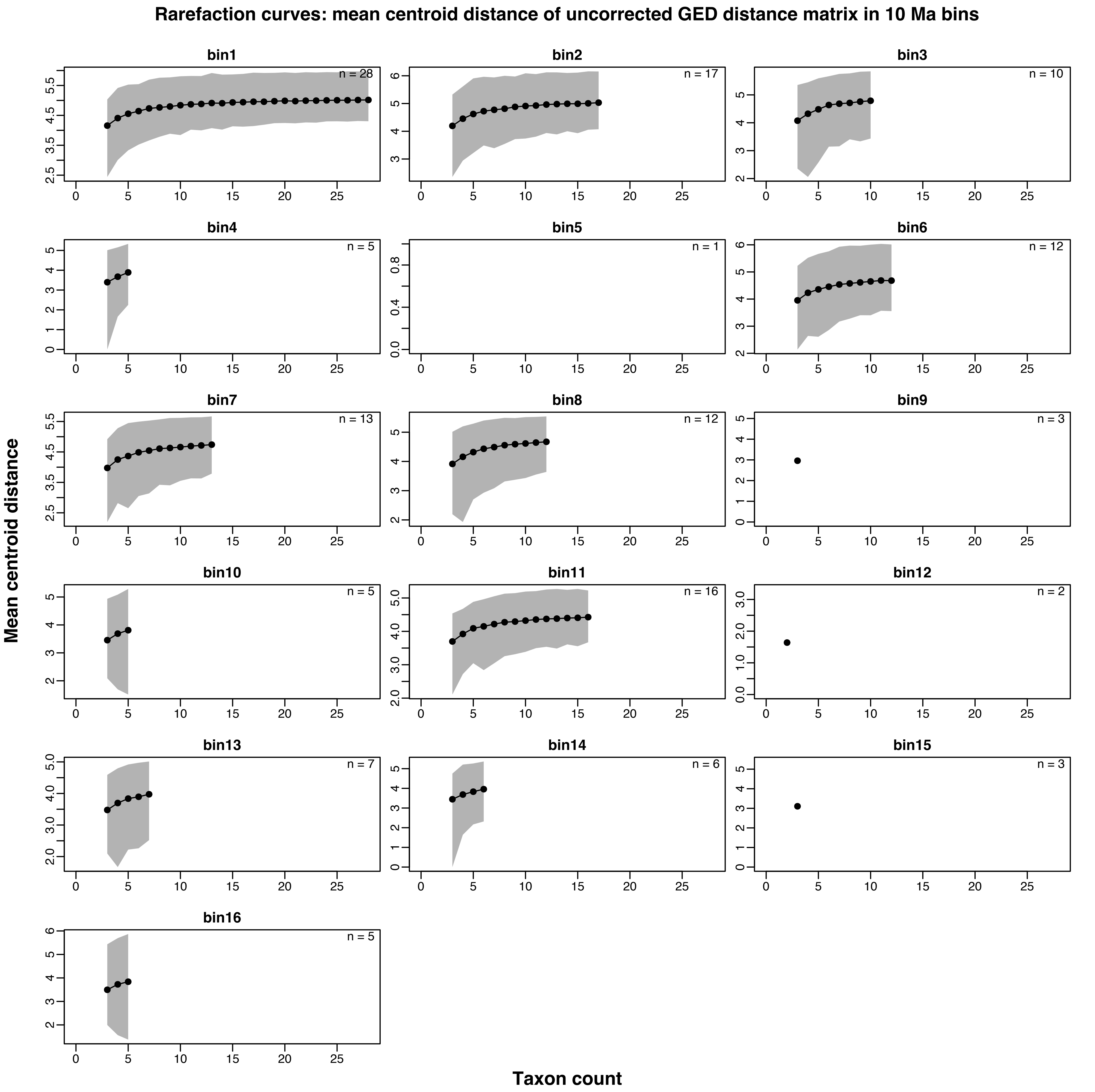

Rarefaction curves: mean centroid distance of uncorrected GED distance matrix in 10 Ma bins
bin1
n = 28
bin2
n = 17
bin3
n = 10
bin4
n = 5
bin5
n = 1
bin6
n = 12
bin7
n = 13
bin8
n = 12
bin9
n = 3
bin10
n = 5
bin11
n = 16
bin12
n = 2
bin13
n = 7
bin14
n = 6
bin15
n = 3
bin16
n = 5
Mean centroid distance
Taxon count

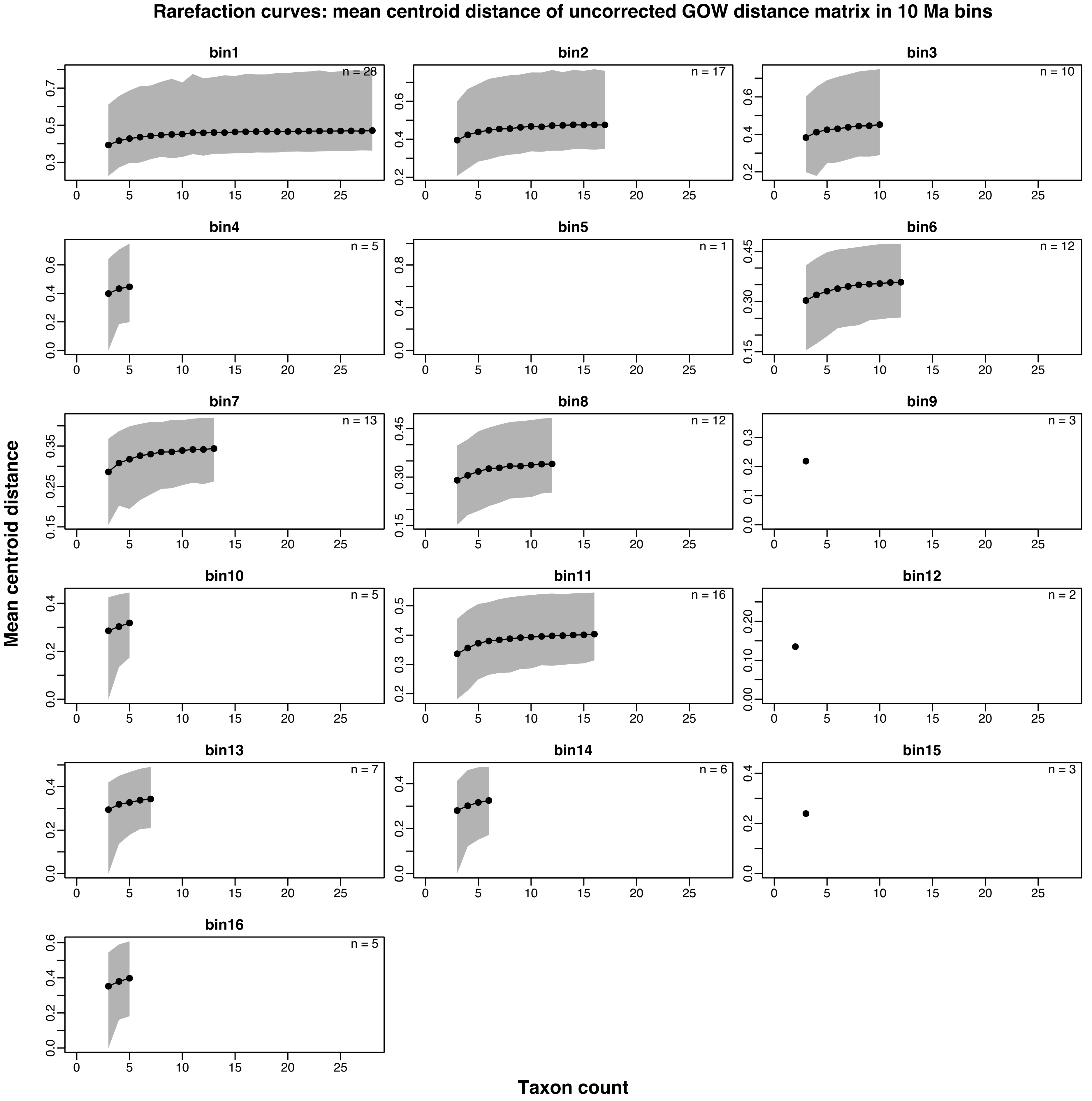
Rarefaction curves: mean centroid distance of uncorrected GOW distance matrix in 10 Ma bins
bin1
n = 28
bin2
n = 17
bin3
n = 10
bin4
n = 5
bin5
n = 1
bin6
n = 12
bin7
n = 13
bin8
n = 12
bin9
n = 3
bin10
n = 5
bin11
n = 16
bin12
n = 2
bin13
n = 7
bin14
n = 6
bin15
n = 3
bin16
n = 5
Mean centroid distance
Taxon count

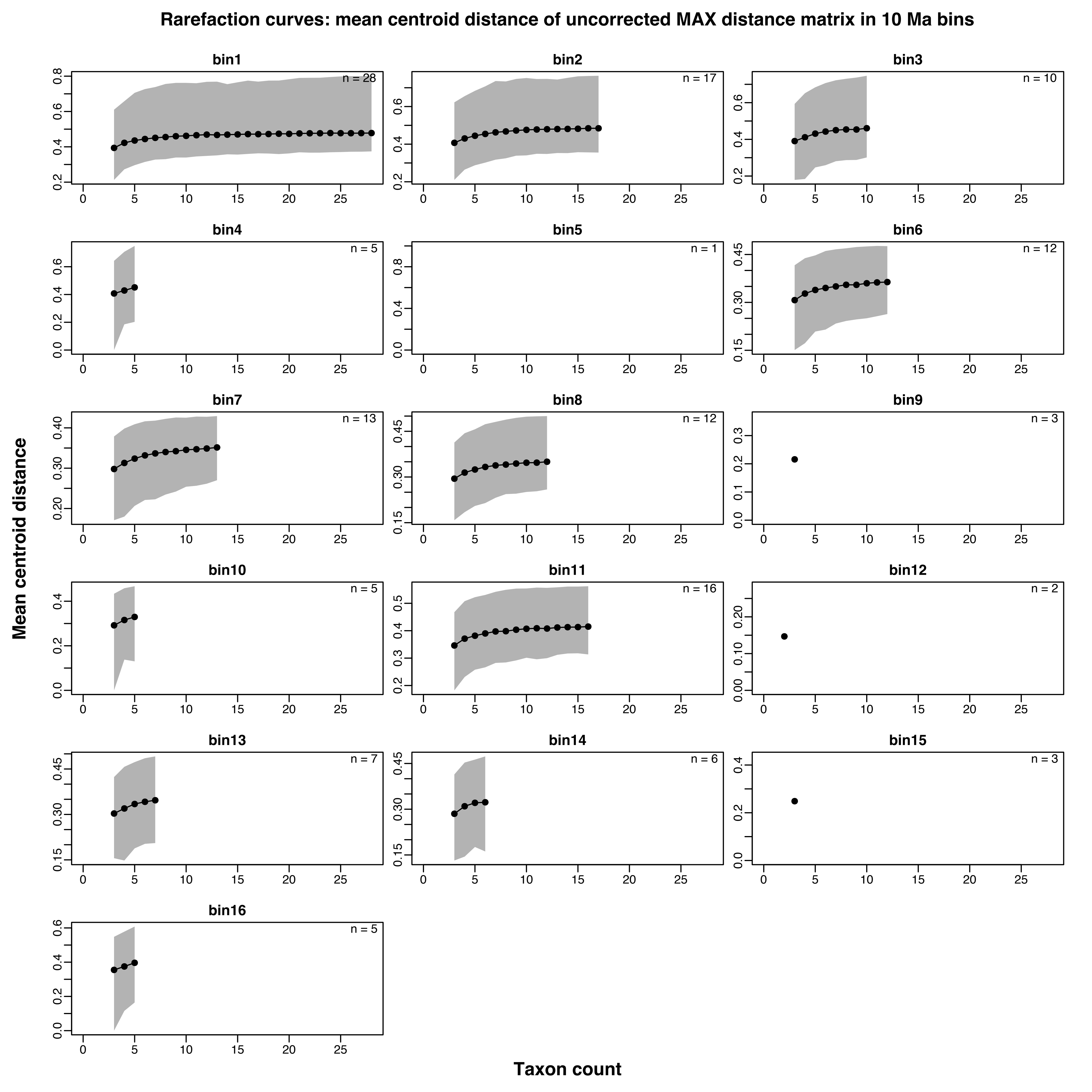
Rarefaction curves: mean centroid distance of uncorrected MAX distance matrix in 10 Ma bins
bin1
n = 28
bin2
n = 17
bin3
n = 10
bin4
n = 5
bin5
n = 1
bin6
n = 12
bin7
n = 13
bin8
n = 12
bin9
n = 3
bin10
n = 5
bin11
n = 16
bin12
n = 2
bin13
n = 7
bin14
n = 6
bin15
n = 3
bin16
n = 5
Mean centroid distance
Taxon count

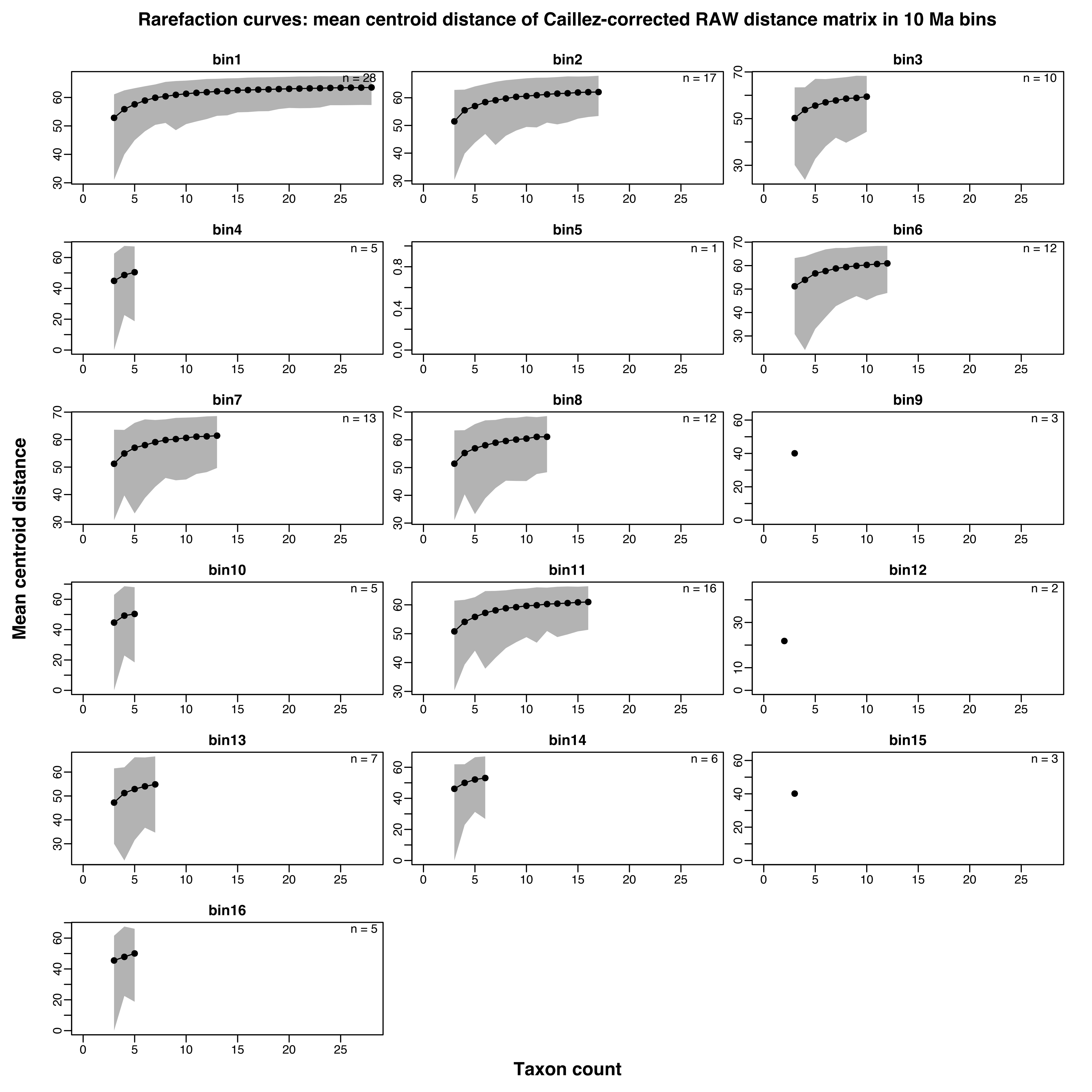
Rarefaction curves: mean centroid distance of Caillez-corrected RAW distance matrix in 10 Ma bins
bin1
n = 28
bin2
n = 17
bin3
n = 10
bin4
n = 5
bin5
n = 1
bin6
n = 12
bin7
n = 13
bin8
n = 12
bin9
n = 3
bin10
n = 5
bin11
n = 16
bin12
n = 2
bin13
n = 7
bin14
n = 6
bin15
n = 3
bin16
n = 5
Mean centroid distance
Taxon count

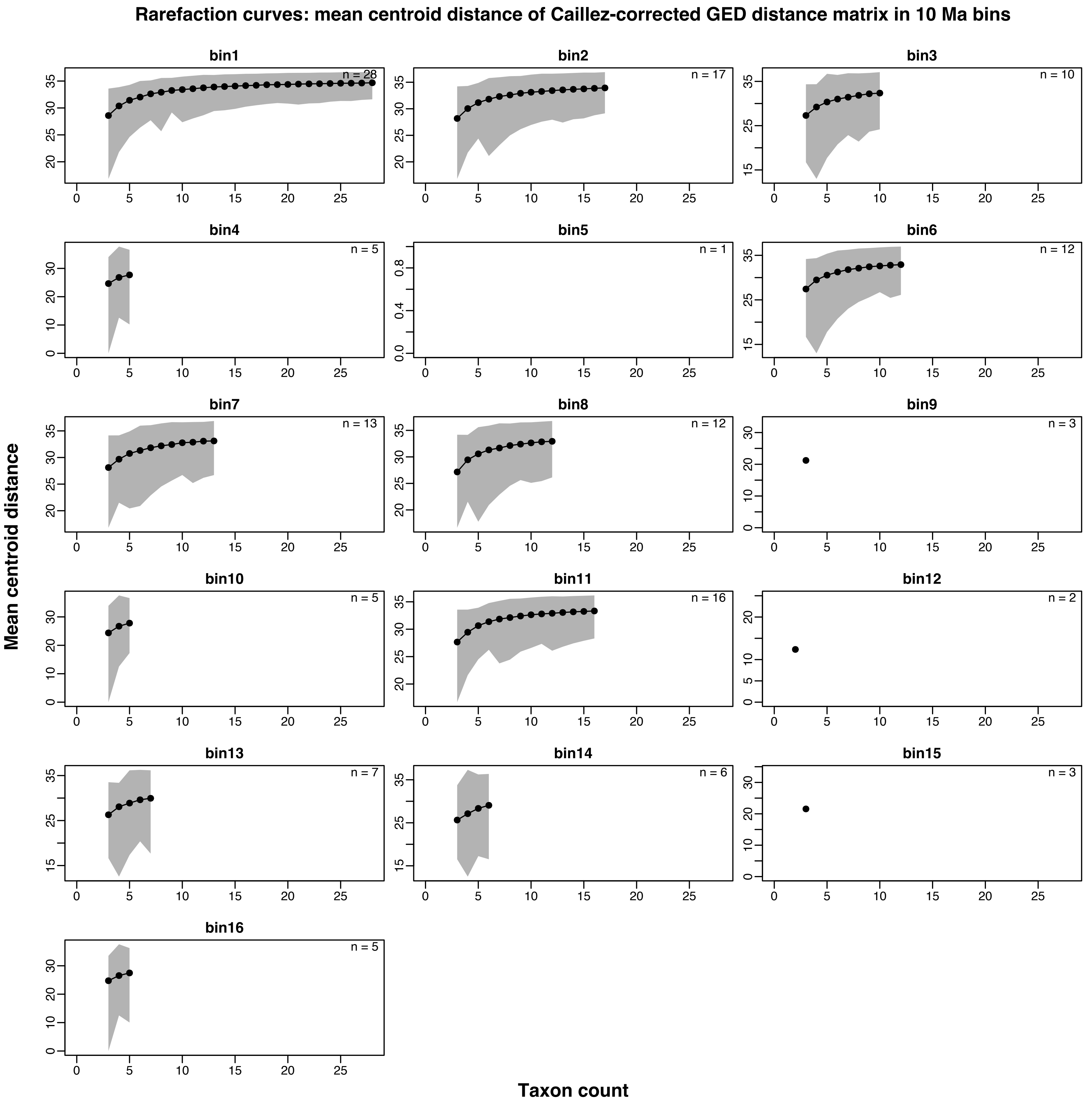

Rarefaction curves: mean centroid distance of Caillez-corrected GED distance matrix in 10 Ma bins
bin1
n = 28
bin2
n = 17
bin3
n = 10
bin4
n = 5
bin5
n = 1
bin6
n = 12
bin7
n = 13
bin8
n = 12
bin9
n = 3
bin10
n = 5
bin11
n = 16
bin12
n = 2
bin13
n = 7
bin14
n = 6
bin15
n = 3
bin16
n = 5
Mean centroid distance
Taxon count

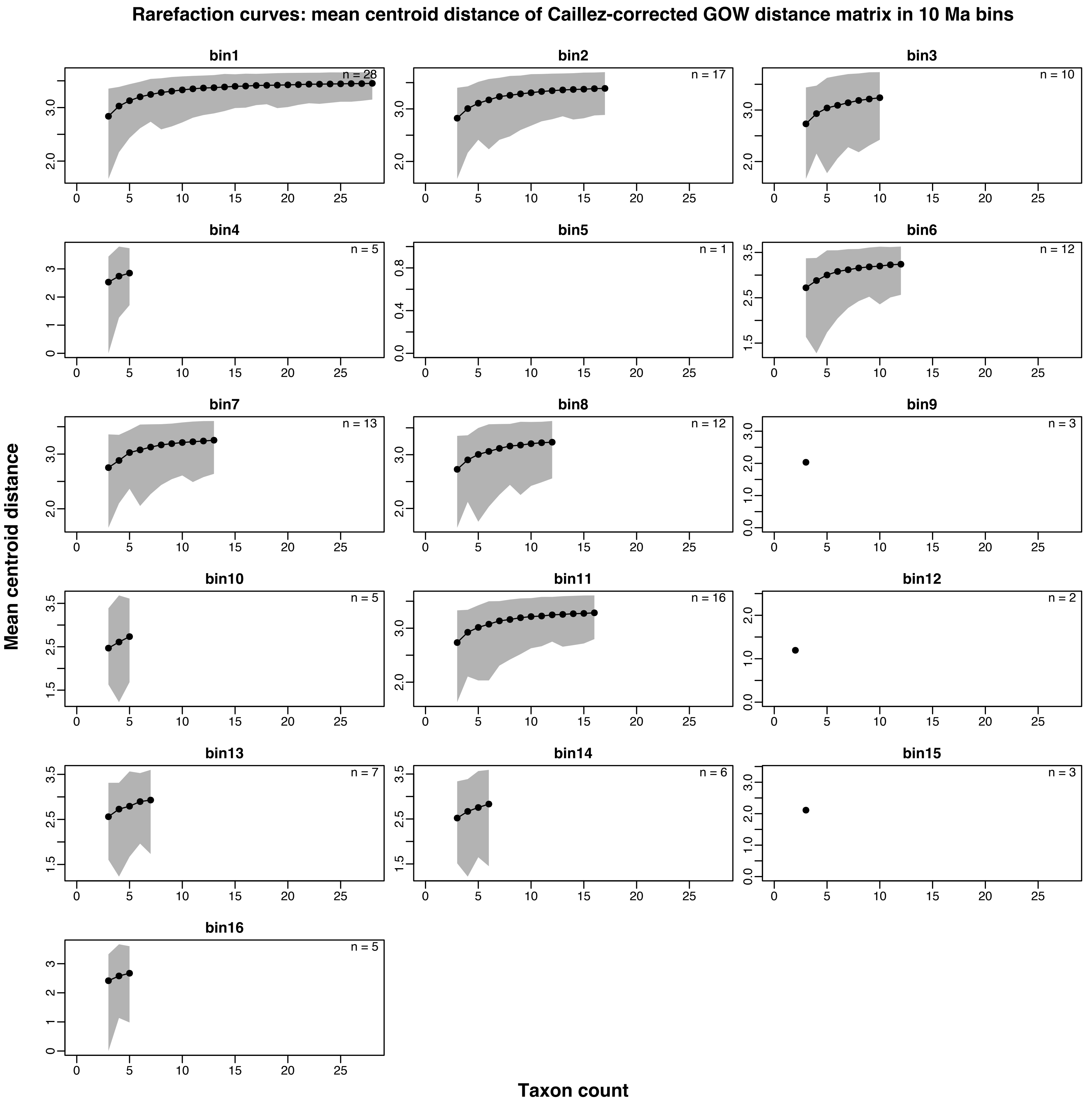

Rarefaction curves: mean centroid distance of Caillez-corrected GOW distance matrix in 10 Ma bins
bin1
n = 28
bin2
n = 17
bin3
n = 10
bin4
n = 5
bin5
n = 1
bin6
n = 12
bin7
n = 13
bin8
n = 12
bin9
n = 3
bin10
n = 5
bin11
n = 16
bin12
n = 2
bin13
n = 7
bin14
n = 6
bin15
n = 3
bin16
n = 5
Mean centroid distance
Taxon count

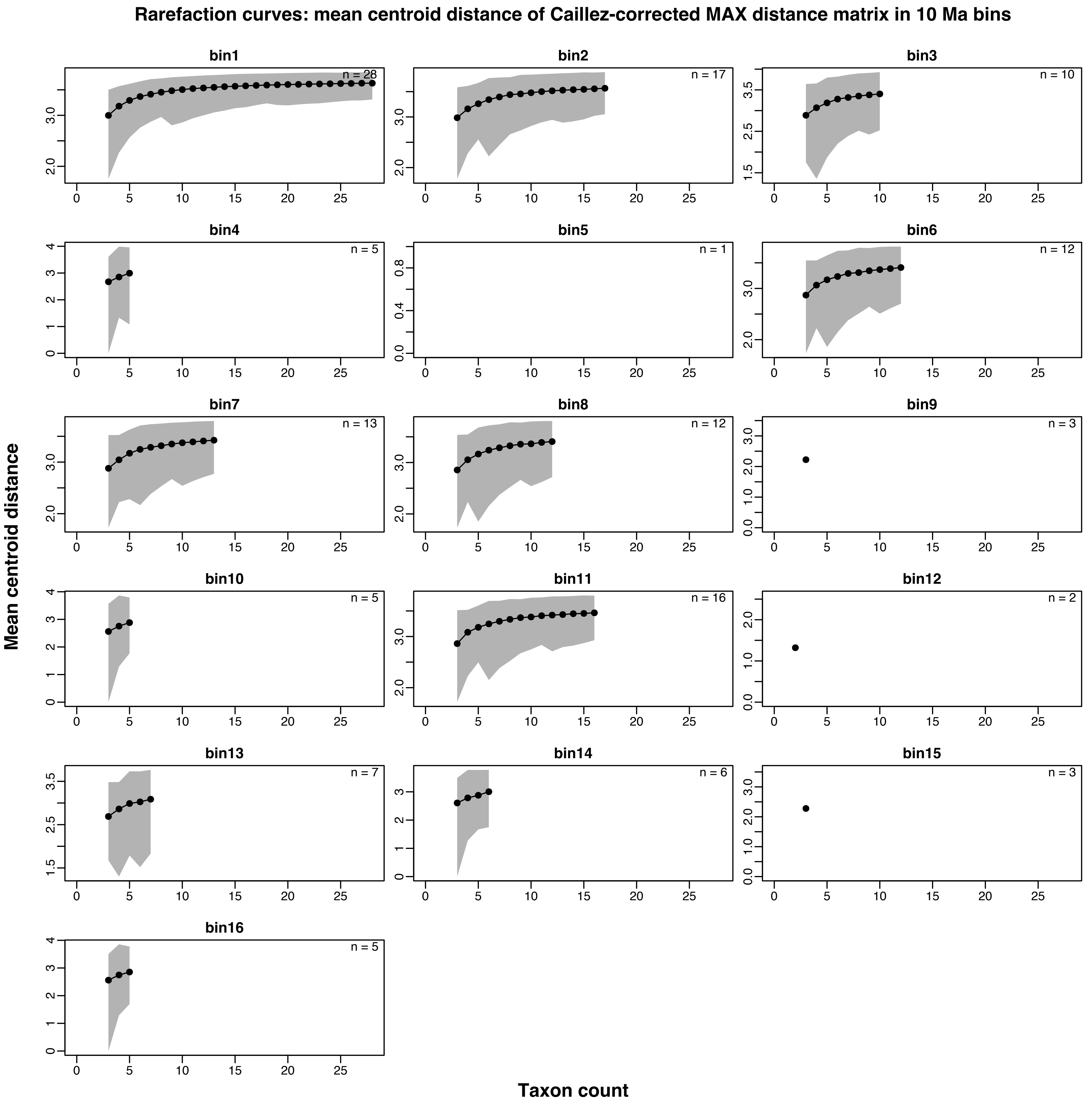

Rarefaction curves: mean centroid distance of Caillez-corrected MAX distance matrix in 10 Ma bins
bin1
n = 28
bin2
n = 17
bin3
n = 10
bin4
n = 5
bin5
n = 1
bin6
n = 12
bin7
n = 13
bin8
n = 12
bin9
n = 3
bin10
n = 5
bin11
n = 16
bin12
n = 2
bin13
n = 7
bin14
n = 6
bin15
n = 3
bin16
n = 5
Mean centroid distance
Taxon count

Supplementary figure 6. (following pages) **Morphospace occupation of Ichthyosauriformes through the Mesozoic.** Principal coordinate axis 1 against axes 2 (top row) and 3 (bottom row) from each of eight PCA (four distance matrices: RAW, GED, GOW, MAX; with and without negative eigenvalue correction) on the cladistic matrix of Moon [1] binned into epochs.

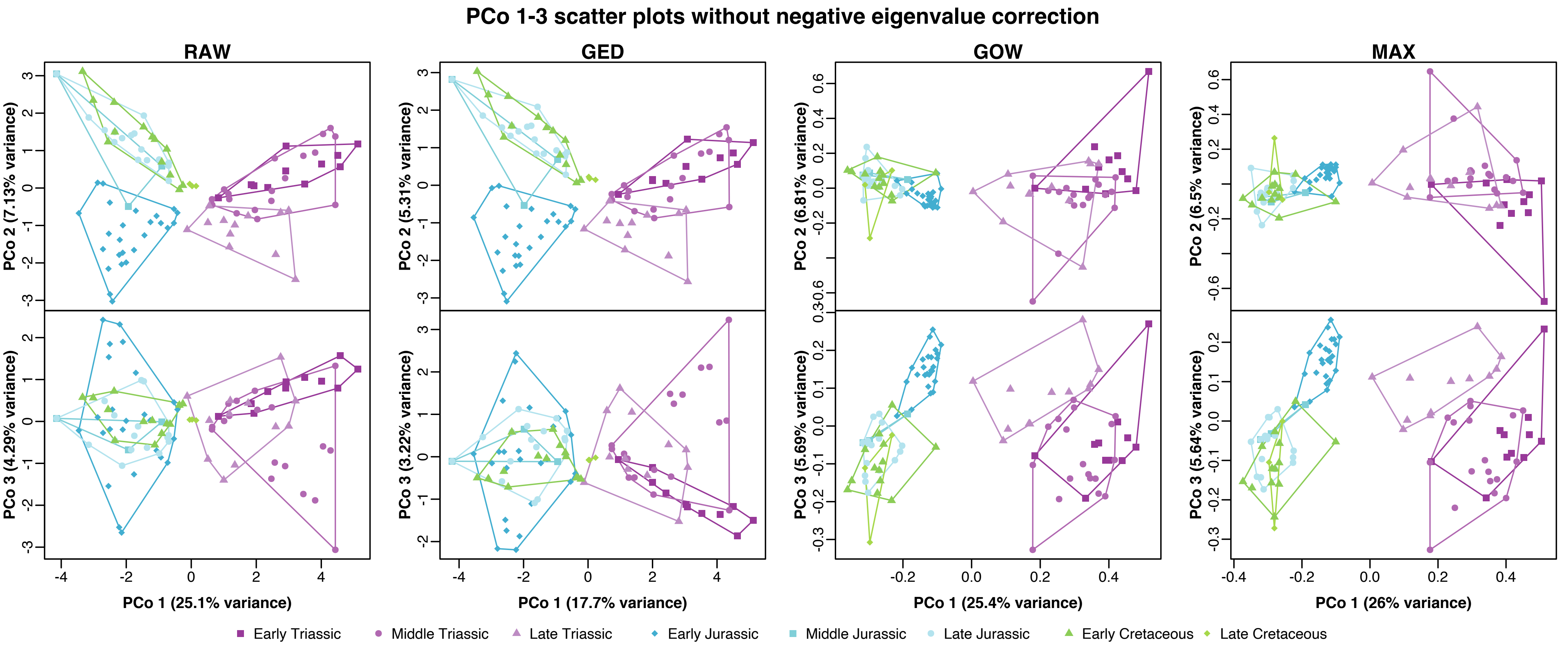

PCo 1-3 scatter plots without negative eigenvalue correction
RAW
GED
GOW
MAX
PCo 2 (7.13% variance)
PCo 3 (4.29% variance)
PCo 1 (25.1% variance)
PCo 2 (5.31% variance)
PCo 3 (3.22% variance)
PCo 1 (17.7% variance)
PCo 2 (6.81% variance)
PCo 3 (5.69% variance)
PCo 1 (25.4% variance)
PCo 2 (6.5% variance)
PCo 3 (5.64% variance)
PCo 1 (26% variance)
Early Triassic
Middle Triassic
Late Triassic
Early Jurassic
Middle Jurassic
Late Jurassic
Early Cretaceous
Late Cretaceous

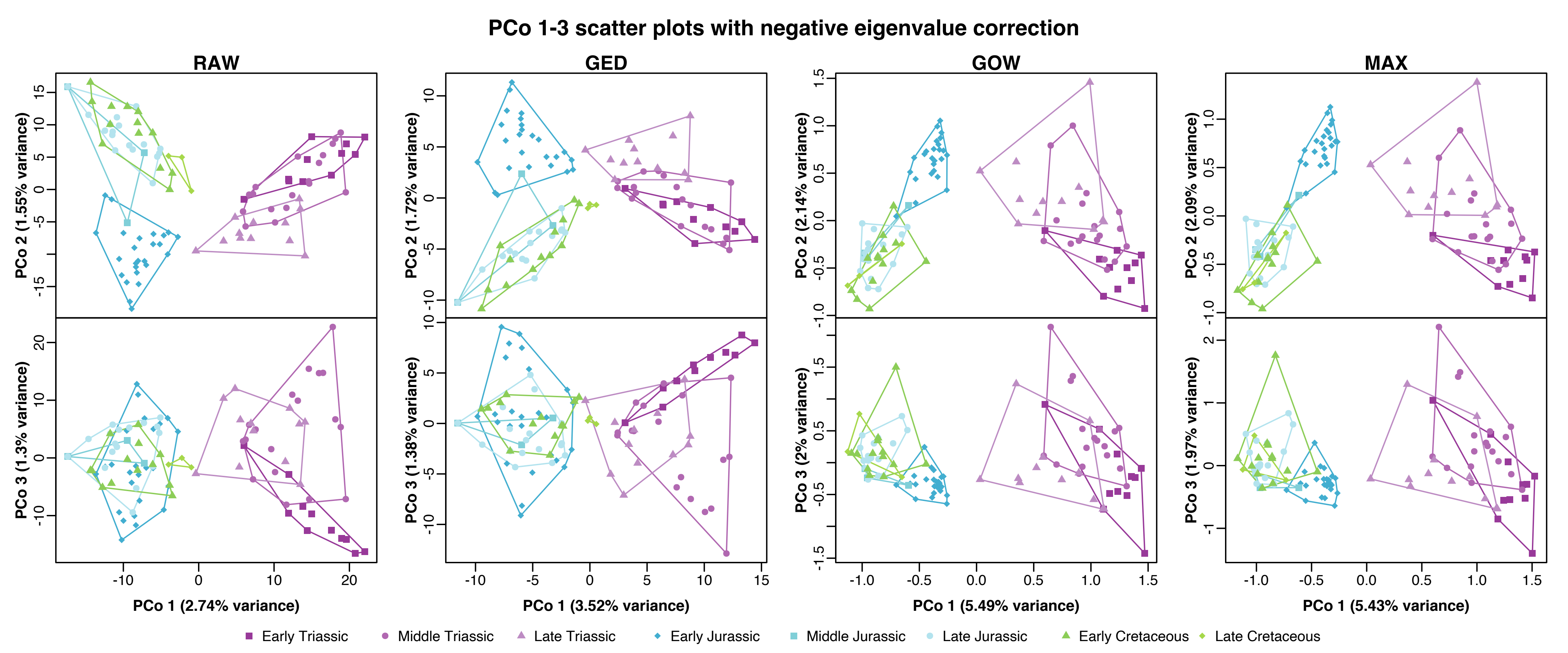
PCo 1-3 scatter plots with negative eigenvalue correction
RAW
GED
GOW
MAX
PCo 2 (1.55% variance)
PCo 3 (1.3% variance)
PCo 1 (2.74% variance)
PCo 2 (1.72% variance)
PCo 3 (1.38% variance)
PCo 1 (3.52% variance)
PCo 2 (2.14% variance)
PCo 3 (2% variance)
PCo 1 (5.49% variance)
PCo 2 (2.09% variance)
PCo 3 (1.97% variance)
PCo 1 (5.43% variance)
Early Triassic
Middle Triassic
Late Triassic
Early Jurassic
Middle Jurassic
Late Jurassic
Early Cretaceous
Late Cretaceous

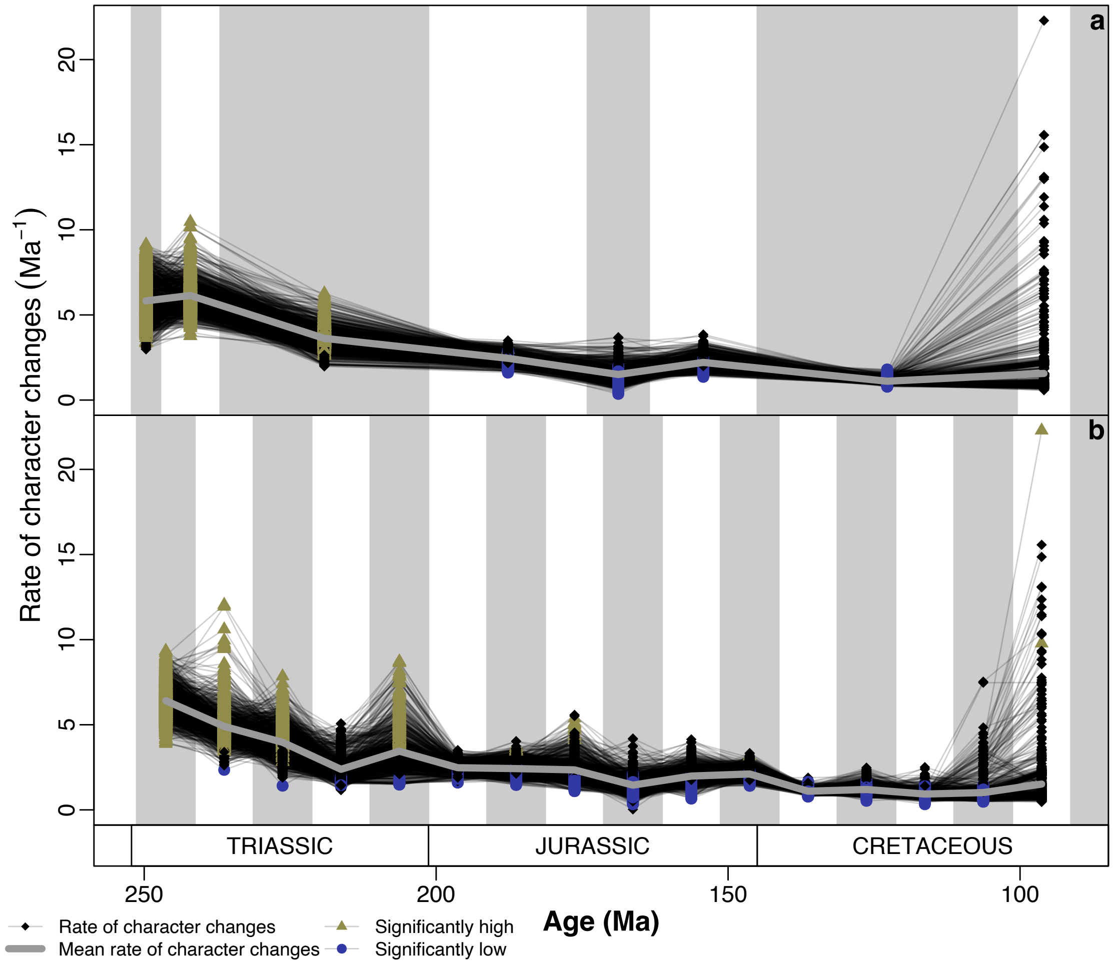

Supplementary figure 7. **Rates of discrete skeletal character evolution in Ichthyosauriformes.** Calculated from the matrix of Moon [1] using 1000 time-scaled trees from the minimum branch length method. Rates of evolution are plotted in **a**, epoch-bins and **b**, equal-length 10-million-year bins.

Supplementary figure 8. (following page) **Rates of skull size evolution in Ichthyosauriformes.** Evolutionary rate results from 100 Hedman-dated phylogenies. Branches are scaled and branches and taxon names coloured to the rate of skull size change on that branch.

ichthyosaur BayesTraits trees

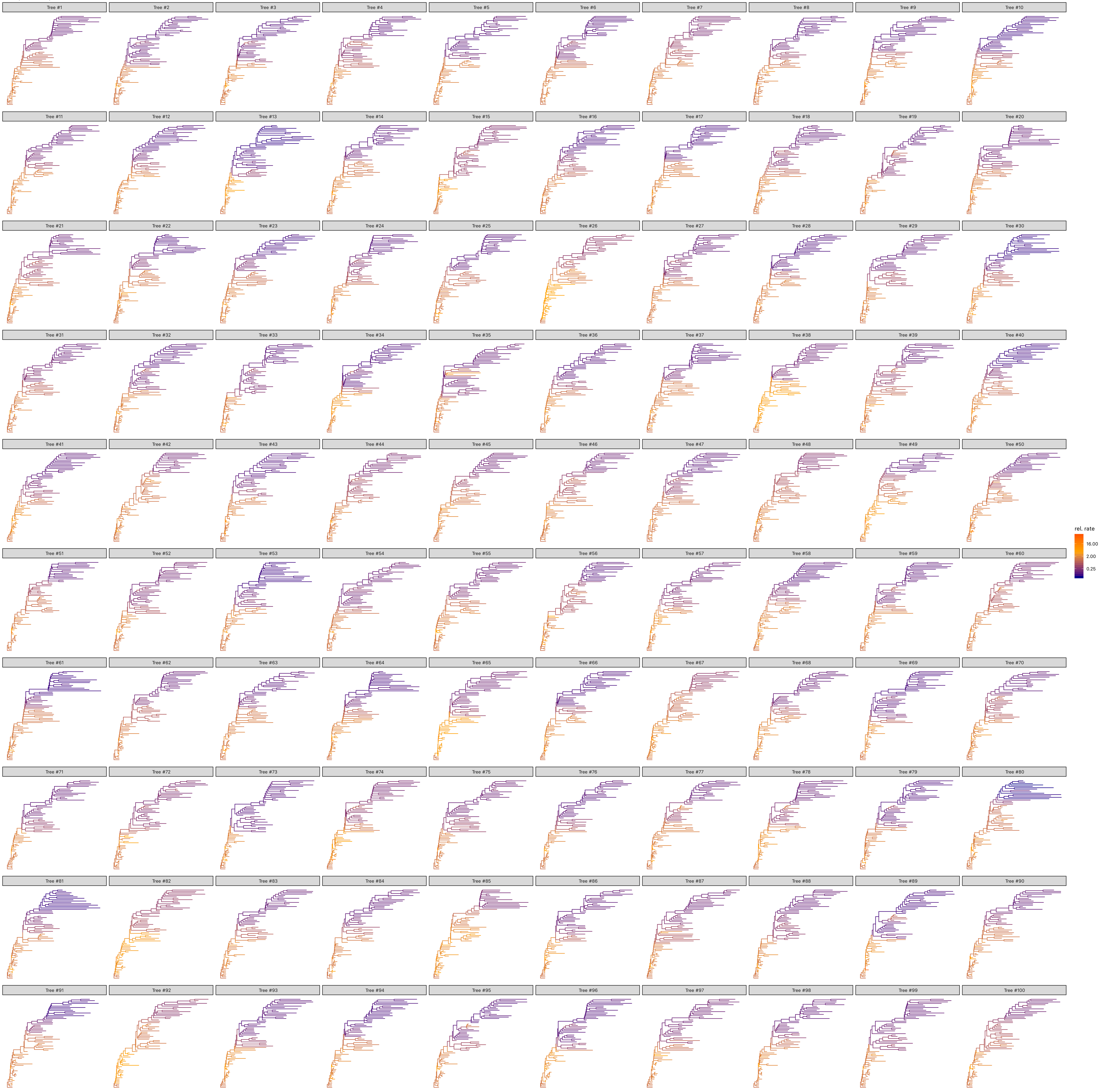

Supplementary tables

1 Bin boundaries of 10 Ma bins used in this study 78
2 Occurrence dates of outgroup taxa used to date the tree of Ichthyosauriformes . 79
3 Occurrence stratigraphy and dates of Ichthyosauriformes included in the analyses 80
4 Skull lengths of Ichthyosauriformes included in the analyses 85

Supplementary table 1. **Bin boundaries of 10 Ma bins used in this study.** Approximate age ranges are given as indicators.

Bin	Start (Ma)	End (Ma)	Approximate age range
1	251.3	241.3	Olenekian–Ladinian
2	241.3	231.3	Ladinian–Carnian
3	231.3	221.3	Carnian–Norian
4	221.3	211.3	Norian
5	211.3	201.3	Norian–Rhaetian
6	201.3	191.3	Hettangian–Sinemurian
7	191.3	181.3	Sinemurian–Toarcian
8	181.3	171.3	Toarcian–Aalenian
9	171.3	161.3	Aalenian–Oxfordian
10	161.3	151.3	Oxfordian–Tithonian
11	151.3	141.3	Tithonian–Berriasian
12	141.3	131.3	Berriasian–Hauterivian
13	131.3	121.3	Hauterivian–Aptian
14	121.3	111.3	Aptian–Albian
15	111.3	101.3	Albian
16	101.3	91.3	Albian–Turonian

Supplementary table 2. **Occurrence dates of outgroup taxa used to date the tree of Ichthyosauriformes.** Stratigraphic occurrence intervals are taken from the given references. Occurrences are converted to absolute ages using Gradstein *et al.* [2]. FAD, first appearance date; FAS, first appearance stratigraphy; LAD, last appearance date; LAS, last appearance stratigraphy.

Taxon	FAD (Ma)	LAD (Ma)	FAS	LAS	Reference
Petrolacosaurus	307.0	298.9	Upper Pennsylvanian	Upper Pennsylvanian	[3, 4]
Hovasaurus	259.8	251.2	Upper Permian	Induan	[5]
Claudiosaurus	253.2	252.17	Late Changsinghian	Late Changinghian	[6]
Thadeosaurus	254.14	252.17	Changsinghian	Changsinghian	[6]
Milleretta	253.2	252.5	Changsinghian	Changsinghian	[7]
Broomia	265.1	260.5	Capitanian	Capitanian	[7]
Mesosaurus	290.1	286	Early Artinskian	Early Artinskian	[7]
Captorhinus	280	270.6	Leonardian	Leonardian	[8]

Supplementary table 3. **Occurrence stratigraphy and dates of Ichthyosauriformes included in the analyses.** Stratographical occurrences are given to the nearest ammonite or conodont biozone horizon where possible. Occurrences are converted to absolute ages using Gradstein *et al.* [2]. FAD, first appearance date; LAD, last appearance date.

Taxon	FAD stratigraphy	LAD stratigraphy	FAD (Ma)	LAD (Ma)	References
Acamptonectes densus	D2D horizon, Speeton Clay Formation, basal Hauterivian	*Simbiskites concinnus/staffi* Biozone, upper Hauterivian, Lower Cretaceous	132.9	129.4	[9]
Aegirosaurus leptospondylus	Malm Zeta2b, early lower Tithonian, Upper Jurassic	Malm Zeta2b, early lower Tithonian, Upper Jurassic	153.96	149.87	[10]
Arthropterygius chrisorum	Oxfordian, Upper Jurassic	Kimmeridgian, Upper Jurassic	163.47	152.06	[11]
Athabascasaurus bitumineus	Lower Albian, Lower Cretaceous	Lower Albian, Lower Cretaceous	113	111.5	[12]
Barracudasauroides panxianensis	*Nicoraella germanicus* Conodont Biozone, Anisian, Middle Triassic	*Nicoraella germanicus* Conodont Biozone, Anisian, Middle Triassic	244.94	243.99	[13]
Besanosaurus leptorhynchus	*Nevadites* Conodont Biozone, uppermost Anisian, Middle Triassic	*Nevadites* Conodont Biozone, uppermost Anisian, Middle Triassic	242.1	241.5	[14]
Brachypterygius extremus	*Pectinatites wheatleyensis* Ammonite Biozone, Tithonian, Upper Jurassic	*Pectinatites hudlestoni* Ammonite Biozone, Tithonian, Upper Jurassic	151	150	[15]
Brachypterygius pseudoscythica	*Ilowaisya pseudoscythica* Ammonite Biozone, Tithonian, Upper Jurassic	*Ilowaisya pseudoscythica* Ammonite Biozone, Tithonian, Upper Jurassic	150.1	149.5	[16]
Californosaurus perrini	*Trachyceras* Beds, Hosselkus Limestone, Carnian	*Trachyceras* Beds, Hosselkus Limestone, Carnian	233.5	228.35	[17, 18]
Callawayia neoscapularis	*Epigondolella triangularis* Conodont Biozone, early Norian, Upper Triassic	*Epigondolella quadrata* Conodont Biozone, early Norian, Upper Triassic	221.5	217.5	[19]
Cartorhynchus lenticarpus	*Subcolumbites* Ammonite Biozone, Olenekian, Lower Triassic	*Subcolumbites* Ammonite Biozone, Olenekian, Lower Triassic	247.7	247.2	[20]
Caypullisaurus bonapartei	*Virgatosphinactes mendozanus* Ammonite Biozone, early Tithonian, Upper Jurassic	Berriasian	152.1	139.4	[21]
Chacaicosaurus cayi	*Emileia giebeli* Ammonite Biozone, early Bajocian, Middle Jurassic	*Emileia giebeli* Ammonite Biozone, early Bajocian, Middle Jurassic	170.3	169.45	[22]
Chaohusaurus chaoxianensis	*Procolumbites* Ammonite Biozone, Olenekian, Lower Triassic	*Subcolumbites* Ammonite Biozone, Olenekian, Lower Triassic	247.9	247.2	[23, 24]
Chaohusaurus geishanensis	*Procolumbites* Ammonite Biozone, Olenekian, Lower Triassic	*Subcolumbites* Ammonite Biozone, Olenekian, Lower Triassic	247.9	247.2	[23, 24]
Chaohusaurus zhangjiawanensis	*Neospathodus homeri* Conodont Biozone, Spathian, Lower Triassic	*Neospathodus triangulus* Conodont Biozone, Spathian, Lower Triassic	247.9	247.2	[25]
Contectopalatus atavus	Pelsonian, Anisian, Middle Triassic	Illyrian, Anisian, Middle Triassic	244.94	241.5	[26]
Cryopterygius kielanae	Middle Volgian, Lower Tithonian, Upper Jurassic	Middle Volgian, Lower Tithonian, Upper Jurassic	149	147	[27]
Cryopterygius kristiansenae	*Dorsoplanites maximus* Ammonite biozone, Tithonian, Upper Jurassic	*Dorsoplanites ilovaiskyi* Ammonite biozone, Tithonian, Upper Jurassic	148.3	147.4	[28]
Cymbospondylus buchseri	Upper Anisian, Middle Triassic	lower Ladinian, Middle Triassic	242.57	240.3	[29]
Cymbospondylus nichollsi	*Kellnerites felsoeoersensis* Ammonite Biozone, Anisian, Middle Triassic	*Kellnerites felsoeoersensis* Ammonite Biozone, Anisian, Middle Triassic	243.99	243.05	[30]

Supplementary table 3 continued

Taxon	FAD stratigraphy	LAD stratigraphy	FAD (Ma)	LAD (Ma)	References
Cymbospondylus petrinus	*Paragondolella* ex gr. *excelsa* Conodont Biozone, Anisian, Middle Triassic	*Paragondolella* ex gr. *excelsa* Conodont Biozone, Anisian, Middle Triassic	243.99	241.5	[18]
Cymbospondylus piscosus	Anisian, Middle Triassic	Anisian, Middle Triassic	247.2	241.5	[18]
Dearcmhara schawcrossi	*Pleydellia aalensis* Ammonite Biozone, Toarcian, Lower Jurassic	*Stephanoceras humphriesianum* Ammonite Biozone, Bajocian, Middle Jurassic	174.43	169.45	[31]
Eurhinosaurus longirostris	*Dactylioceras tenuicostatum* Ammonite Biozone, Toarcian	*Harpoceras falciferum* Ammonite Biozone, Toarcian	182.7	180.36	[32, 33]
Excalibosaurus costini	*Arietites bucklandi* Ammonite Biozone, lower Sinemurian, Lower Jurassic	*Arietites bucklandi* Ammonite Biozone, lower Sinemurian, Lower Jurassic	199.3	197.8	[33, 34]
Gengasaurus nicosiai	Calcari ad aptici e Saccocoma Formation, Late Kimmeridgian, Upper Jurassic	earliest Tithonian, Upper Jurassic	155	150	[35]
Grendelius alekseevi	*Dorsoplanites panderi* Ammonite Biozone, Tithonian, Upper Jurassic	*Dorsoplanites panderi* Ammonite Biozone, Tithonian, Upper Jurassic	149.6	147.9	[36]
Grendelius zhuravlevi	Middle Volgian, Lower Tithonian, Upper Jurassic	Middle Volgian, Lower Tithonian, Upper Jurassic	149.6	147.9	[37]
Grippia longirostris	*Subcolumbites* Ammonite Biozone, Olenekian, Lower Triassic	*Subcolumbites* Ammonite Biozone, Olenekian, Lower Triassic	247.7	247.2	[38]
Guizhouichthyosaurus tangae	Carnian, Upper Triassic	Carnian, Upper Triassic	233.5	228.35	[39]
Guizhouichthyosaurus wolonggangense	Carnian, Upper Triassic	Carnian, Upper Triassic	233.5	228.35	[40]
Gulosaurus helmi	*Subcolumbites* Ammonite Biozone, Olenekian, Lower Triassic	*Subcolumbites* Ammonite Biozone, Olenekian, Lower Triassic	247.7	247.2	[41, 42]
Hauffiopteryx typicus	*Dactylioceras tenuicostatum*, Ammonite Biozone, Toarcian	*Harpoceras falciferum* Ammonite Biozone, Toarcian	182.7	181.25	[43–45]
Himalayasaurus tibetensis	Norian, Upper Triassic	Norian, Upper Triassic	228.4	209.5	[46]
Hudsonelpidia brevirostris	*Epigondolella quadrata* Conodont Biozone, Norian, Upper Triassic	*Epigondolella quadrata* Conodont Biozone, Norian, Upper Triassic	226.5	221.25	[47]
Hupehsuchus nanchangensis	*Subcolumbites* Ammonite Biozone, Olenekian, Lower Triassic	*Subcolumbites* Ammonite Biozone, Olenekian, Lower Triassic	247.9	247.2	[48]
Ichthyosaurus acutirostris	*Hildoceras bifrons* Ammonite Biozone, Toarcian, Lower Jurassic	*Dactylioceras commune* Ammonite Biozone, Toarcian, Lower Jurassic	180.36	175.6	[49]
Ichthyosaurus anningae	Asteroceras obtusum*?* Ammonite Biozeon, Sinemurian, Lower Jurassic	*Uptonia jamesoni* Ammonite Biozone, Pliensbachian, Lower Jurassic	193.81	189.35	[50]
Ichthyosaurus breviceps	*Schlotheimia angulata* Ammonite Biozone, Sinemurian, Lower Jurassic	*Arnioceras semicostatum* Ammonite Biozone, Sinemurian, Lower Jurassic	200.1	196.31	[51]
Ichthyosaurus communis	Upper Rhaetian, Upper Triassic	*Arnioceras semicostatum* Ammonite Biozone, Lower Jurassic	201.3	196.31	[51]
Ichthyosaurus conybeari	*Schlotheimia angulata* Ammonite Biozone, Hettangian	*Arnioceras semicostatum* Ammonite Biozone, Sinemurian	200.1	196.31	[51]
Ichthyosaurus larkini	Pre-*Planorbis* beds, Hettangian, Lower Jurassic	Pre-*Planorbis* beds, Hettangian, Lower Jurassic	201.3	200.85	[52]
Ichthyosaurus somersetensis	Pre-*Planorbis* beds, Hettangian, Lower Jurassic	Pre-*Planorbis* beds, Hettangian, Lower Jurassic	201.3	200.85	[52]

Supplementary table 3 continued

Taxon	FAD stratigraphy	LAD stratigraphy	FAD (Ma)	LAD (Ma)	References
Isfjordosaurus minor	*Subcolumbites* Ammonite Biozone, Olenekian, Lower Triassic	*Subcolumbites* Ammonite Biozone, Olenekian, Lower Triassic	247.7	247.2	[53]
Janusaurus lundi	*Dorsoplanites maximus* Ammonite-Biozone, Tithonian, Upper Jurassic	*Dorsoplanites ilovaiskyi* Ammonite-Biozone, Tithonian, Upper Jurassic	148.3	147.4	[54]
Keilhauia nui	Slottsmøya Member, Agardfjellet Formation, Berriasian, Lower Cretaceous	Slottsmøya Member, Agardfjellet Formation, Berriasian, Lower Cretaceous	145	143	[55]
Leninia stellans	*Deshayesites volgensis* Ammonite Biozone, Lower Aptian, Lower Cretaceous	*Deshayesites volgensis* Ammonite Biozone, Lower Aptian, Lower Cretaceous	126.3	123	[56]
Leptonectes moorei	Lower Pliensbachian, Lower Jurassic	Lower Pliensbachian, Lower Jurassic	190.82	187.56	[57]
Leptonectes solei	*Arnioceras semicostatum* Ammonite Biozone, Sinemurian, Lower Jurassic	*Asteroceras obtusum* Ammonite Biozone, Sinemurian, Lower Jurassic	195.31	193.81	[58]
Leptonectes tenuirostris	Pre-*Planorbis* beds, Hettangian, Lower Jurassic	*Amaltheus margaritatus* Ammonite Biozone, Pliensbachian, Lower Jurassic	201.3	190.8	[49, 59]
Macgowania janiceps	*Epigondolella matthewi* Conodont Biozone, middle Norian, Upper Triassic	*Epigondolella multidentata* Conodont Biozone, middle Norian, Upper Triassic	220	216.9	[60]
Maiaspondylus lindoei	Middle Albian, Lower Cretaceous	Middle Albian, Lower Cretaceous	111.27	110.22	[61]
Malawania anachronus	Late Hauterivian, Early Cretaceous	Barremian, Early Cretaceous	131	125	[62]
Mikadocephalus gracilirostris	Upper Illyrian, Anisian, Middle Triassic	lower Fassinian, Ladinian, Middle Triassic	242.57	240.3	[63]
Mixosaurus cornalianus	Upper Illyrian, Anisian, Middle Triassic	lower Fassinian, Ladinian, Middle Triassic	242.57	240.3	[49, 64]
Mixosaurus kuhnschneyderi	Upper Illyrian, Anisian, Middle Triassic	lower Fassinian, Ladinian, Middle Triassic	242.57	240.3	[65]
Mixosaurus xindianensis	*Nicoraella kockeli* Conodont Biozone, Pelsonian, Anisian, Middle Triassic	*Nicoraella kockeli* Conodont Biozone, Pelsonian, Anisian, Middle Triassic	244.94	241.5	[66]
Mollesaurus periallus	*Emileia giebeli* Ammonite Biozone, early Bajocian, Middle Jurassic	*Emileia giebeli* Ammonite Biozone, early Bajocian, Middle Jurassic	170.3	169.45	[67, 68]
Muiscasaurus catheti	Barremian , Lower Cretaceous	Aptian, Lower Cretaceous	130.77	115.64	[69]
Nannopterygius enthekiodon	*Aulacostephanus* sp. Ammonite Biozone, Kimmeridgian, Upper Jurassic	*Aulacostephanus* sp. Ammonite Biozone, Tithonian, Upper Jurassic	154.6	149.87	[15]
Ophthalmosaurus icenicus	*Kosmoceras jasoni* Ammonite Biozone, Callovian, Upper Jurassic	*Quenstedtoceras mariae* Ammonite Biozone, Oxfordian, Upper Jurassic	165.59	161.39	[70]
Ophthalmosaurus natans	Oxfordian, Late Jurassic	Oxfordian, Late Jurassic	163.5	157.3	[71]
Ophthalmosaurus yasykovi	*Epivirgatites nikitini* Ammonite Biozone, Tithonian, Upper Jurassic	*Craspedites subdites* Ammonite Biozone, Tithonian, Upper Jurassic	147.5	146.4	[72]
Palvennia hoybergeti	*Dorsoplanites maximus* Ammonite Biozone, Tithonian, Upper Jurassic	*Dorsoplanites ilovaiskyi* Ammonite Biozone, Tithonian, Upper Jurassic	148.3	147.4	[28]
Paraophthalmosaurus kabanovi	*Epivirgatites nikitini* Ammonite Biozone, Tithonian, Upper Jurassic	*Epivirgatites nikitini* Ammonite Biozone, Tithonian, Upper Jurassic	147.5	146.9	[72]
Paraophthalmosaurus saveljeviensis	*Dorsoplanites panderi* Ammonite Biozone, Tithonian, Upper Jurassic	*Epivirgatites nikitini* Ammonite Biozone, Tithonian, Upper Jurassic	149.6	146.9	[73]

Supplementary table 3 continued

Taxon	FAD stratigraphy	LAD stratigraphy	FAD (Ma)	LAD (Ma)	References
Parvinatator wapitiensis	*Subcolumbites* Ammonite Biozone, Olenekian, Lower Triassic	*Subcolumbites* Ammonite Biozone, Olenekian, Lower Triassic	247.7	247.2	[74]
Pervushovisaurus bannovkensis	Middle Cenomanion, Upper Cretaceous	Middle Cenomanion, Upper Cretaceous	96.24	95.47	[75]
Pervushovisaurus campylodon	Early Cenomanian, Upper Cretaceous	Early Cenomanian, Upper Cretaceous	100.45	96.5	[76]
Pessopteryx nisseri	*Subcolumbites* Ammonite Biozone, Olenekian, Lower Triassic	*Subcolumbites* Ammonite Biozone, Olenekian, Lower Triassic	247.7	247.2	[53]
Phalarodon callawayi	Upper Anisian, Middle Triassic	Upper Anisian, Middle Triassic	243.99	239.1	[77]
Phalarodon fraasi	Upper Anisian, Middle Triassic	lower Ladinian, Middle Triassic	244.94	237	[78]
Phalarodon major	Upper Anisian , Middle Triassic	Ladinian, Middle Triassic	244.94	237	[79]
Phantomosaurus neubigi	*pulcher/robustus* Conodont Biozone, Upper Anisian, Middle Triassic	*pulcher/robustus* Conodont Biozone, Upper Anisian, Middle Triassic	244.94	241.5	[80]
Platypterygius americanus	Upper Albian, Lower Cretaceous	Upper Albian, Lower Cretaceous	107.59	100.5	[81]
Platypterygius australis	Albian, Lower Cretaceous	Albian, Lower Cretaceous	113	100.5	[82, 83]
Platypterygius hauthali	*Hatchiceras* Ammonite Biozone, Lower Barremian, Lower Cretaceous	*Hatchiceras* Ammonite Biozone, Lower Barremian, Lower Cretaceous	130.77	129.41	[84]
Platypterygius hercynicus	Aptian, Lower Cretaceous	Aptian, Lower Cretaceous	125	113	[85]
Platypterygius platydactylus	*Hoplites deshayesi* Ammonite Biozone, Aptian, Lower Cretaceous	*Hoplites deshayesi* Ammonite Biozone, Aptian, Lower Cretaceous	125	113	[86]
Platypterygius sachicarum	Barremian, Lower Cretaceous	Aptian, Lower Cretaceous	130.77	113	[87]
Qianichthyosaurus xingyiensis	Ladinian, Middle Triassic	Ladinian, Middle Triassic	241.5	237	[88]
Qianichthyosaurus zhoui	Carnian, Upper Triassic	Carnian, Upper Triassic	237	228.35	[89]
Quasianosteosaurus vikinghoegdai	*Subcolumbites* Ammonite Biozone, Olenekian, Lower Triassic	*Subcolumbites* Ammonite Biozone, Olenekian, Lower Triassic	247.7	247.2	[90]
Sclerocormus parviceps	*Subcolumbites* Ammonite Biozone, Olenekian, Lower Triassic	*Subcolumbites* Ammonite Biozone, Olenekian, Lower Triassic	247.9	247.7	[91, 92]
Shastasaurus liangae	Upper Carnian, Late Triassic	Upper Carnian, Late Triassic	233.5	228.35	[93]
Shastasaurus pacificus	Upper Carnian, Late Triassic	Upper Carnian, Late Triassic	233.5	228.35	[18]
Shastasaurus sikkaniensis	*Epigondolella postera* conodont Biozone, *Mesohemavatities columbianus* ammonite Biozone, middle Norian, Upper Triassic	*Epigondolella postera* conodont Biozone, *Mesohemavatities columbianus* ammonite Biozone, middle Norian, Upper Triassic	216.9	214.7	[94]
Shonisaurus popularis	Upper Carnian, Late Triassic	Upper Carnian, Late Triassic	233.5	228.35	[95]
Simbirskiasaurus birjukovi	*Praeoxyteuthis pugio* Belemnite Biozone, lower Barremian, Lower Cretaceous	*Praeoxyteuthis pugio* Belemnite Biozone, lower Barremian, Lower Cretaceous	130.77	129.41	[75]
Sisteronia seeleyi	Cambridge Greensand Member, early Cenomanian, Upper Cretaceous	Cambridge Greensand Member, early Cenomanian, Upper Cretaceous	100.5	96.24	[96]
Stenopterygius aaleniensis	*Leioceras opalinum* Ammonite Biozone, Aalenian, Middle Jurassic	*Leioceras opalinum* Ammonite Biozone, Aalenian, Middle Jurassic	174.1	172.13	[97]
Stenopterygius quadriscissus	*Dactylioceras tenuicostatum* Ammonite Biozone, Toarcian, Lower Jurassic	*Harpoceras falciferum* Ammonite Biozone, Toarcian, Lower Jurassic	182.7	180.36	[43, 98]
Stenopterygius triscissus	*Dactylioceras tenuicostatum* Ammonite Biozone, Toarcian, Lower Jurassic	*Harpoceras falciferum* Ammonite Biozone, Toarcian, Lower Jurassic	182.7	180.36	[43, 98]

Supplementary table 3 continued

Taxon	FAD stratigraphy	LAD stratigraphy	FAD (Ma)	LAD (Ma)	References
Stenopterygius uniter	*Harpoceras falciferum* Ammonite Biozone, Toarcian, Lower Jurassic	*Harpoceras falciferum* Ammonite Biozone, Toarcian, Lower Jurassic	181.7	180.36	[43, 98]
Suevoleviathan disinteger	*Dactylioceras tenuicostatum* Ammonite Biozone, Toarcian, Lower Jurassic	*Harpoceras falciferum* Ammonite Biozone, Toarcian, Lower Jurassic	181.7	180.36	[99, 100]
Suevoleviathan integer	*Dactylioceras tenuicostatum* Ammonite Biozone, Toarcian, Lower Jurassic	*Harpoceras falciferum* Ammonite Biozone, Toarcian, Lower Jurassic	181.7	180.36	[99, 100]
Sveltonectes insolitus	Upper Barremian, Cretaceous	Upper Barremian, Cretaceous	129.6	126.3	[101]
Temnodontosaurus azerguensis	*Harpoceras bifrons* Ammonite Biozone, middle Toarcian, Lower Jurassic	*Harpoceras bifrons* Ammonite Biozone, middle Toarcian, Lower Jurassic	180.36	178.24	[102]
Temnodontosaurus crassimanus	*Dactylioceras tenuicostatum* Ammonite Biozone, Toarcian, Lower Jurassic	*Harpoceras falciferum* Ammonite Biozone, Toarcian, Lower Jurassic	182.7	180.36	[103]
Temnodontosaurus eurycephalus	*Arietites bucklandi* Ammonite Biozone, lower Sinemurian, Lower Jurassic	*Arietites bucklandi* Ammonite Biozone, lower Sinemurian, Lower Jurassic	199.3	197.8	[104]
Temnodontosaurus nuertingensis	*Uptonia jamesoni* Ammonite Biozone, Lower Pliensbachian, Lower Jurassic	*Tragophyllocera ibex* Ammonite Biozone, Pliensbachian, Lower Jurassic	190.8	187.56	[105, 106]
Temnodontosaurus platyodon	*Schlotheimia angulata* Ammonite Biozone, Hettangian	*Arnioceras semicostatum* Ammonite Biozone, Sinemurian	200.1	196.31	[104]
Temnodontosaurus trigonodon	*Dactylioceras tenuicostatum* Ammonite Biozone, Toarcian, Lower Jurassic	*Harpoceras falciferum* Ammonite Biozone, Toarcian, Lower Jurassic	182.7	180.36	[99, 107]
Thaisaurus chonglakmanii	Lower Triassic	Lower Triassic	250.01	247.2	[108]
Thalattoarchon saurophagis	*Nevadisculites taylori* Ammonite Biozone, Anisian, Middle Triassic	*Nevadisculites taylori* Ammonite Biozone, Anisian, Middle Triassic	247.2	244.6	[109]
Tholodus schmidi	*Decurtella decurtata* Conodont Biozone, Ladinian (Pelsonian), Middle Triassic	*Judicarites/Neoschizodus orbicularis* Conodont Biozone, Ladinian (Pelsonian), Middle Triassic	241	237	[110]
Toretocnemus californicus	*Metapolygnathus polygnathiformis* Conodont Biozone, Carnian, Upper Triassic	*Metapolygnathus polygnathiformis* Conodont Biozone, Carnian, Upper Triassic	233.5	228.35	[49, 111]
Toretocnemus zitteli	*Metapolygnathus polygnathiformis* Conodont Biozone, Carnian, Upper Triassic	*Metapolygnathus polygnathiformis* Conodont Biozone, Carnian, Upper Triassic	233.5	228.35	[49, 111]
Undorosaurus gorodischensis	*Epivirgatites nikitini*, Virgatites virgatus AmmoniteBiozone, Tithonian, Upper Jurassic	*Epivirgatites nikitini*, Virgatites virgatus AmmoniteBiozone, Tithonian, Upper Jurassic	147.9	146.9	[112]
Undorosaurus trautscholdi	*Epivirgatites nikitini* Ammonite Biozone, Tithonian, Upper Jurassic	*Kachpurites fulgens* Ammonite Biozone, Tithonian, Upper Jurassic	150	144.1	[113]
Utatsusaurus hataii	*Subcolumbites* Ammonite Biozone, Olenekian, Lower Triassic	*Arnautoceratites* Ammonite Biozone, Olenekian, Lower Triassic	247.7	247.2	[114]
Wahlisaurus massarae	Pre-*Planorbis* beds, Hettangian, Lower Jurassic	Pre-*Planorbis* beds, Hettangian, Lower Jurassic	201.3	200.85	[115]
Wimanius odontopalatus	Anisian , Middle Triassic	Ladinian, Middle Triassic	241.5	237	[116]
Xinminosaurus catactes	*Nicoraella kockeli* Conodont Biozone, Pelsonian, Anisian, Middle Triassic	*Nicoraella kockeli* Conodont Biozone, Pelsonian, Anisian, Middle Triassic	244.94	241.5	[117]

Supplementary table 4. **Skull lengths of Ichthyosauriformes included in the analyses.** Logarithm values are shown to 3 d.p.

Taxon	Skull length (mm)	$\log_{10}$ (Skull length (mm))	References
Acamptonectes densus	1000	3.000	[9]
Aegirosaurus leptospondylus	570	2.756	[10]
Barracudasauroides panxianensis	212	2.326	[13]
Besanosaurus leptorhynchus	510	2.708	[14]
Brachypterygius extremus	1155	3.062	[15]
Callawayia neoscapularis	453	2.656	[80, 118]
Cartorhynchus lenticarpus	55	1.740	[20]
Caypullisaurus bonapartei	1300	3.114	[21]
Chacaicosaurus cayi	980	2.991	[22]
Chaohusaurus geishanensis	117	2.068	[23, 24]
Contectopalatus atavus	130	2.114	[26, 119]
Cryopterygius kristiansenae	1220	3.086	[28]
Cymbospondylus petrinus	1166	3.067	[18]
Eurhinosaurus longirostris	1250	3.097	[32, 33]
Excalibosaurus costini	1540	3.188	[33, 34]
Guizhouichthyosaurus tangae	800	2.903	[39]
Guizhouichthyosaurus wolonggangense	645	2.810	[40]
Gulosaurus helmi	87	1.940	[41, 42]
Hauffiopteryx typicus	380	2.580	[43–45]
Hudsonelpidia brevirostris	131	2.117	[47]
Hupehsuchus nanchangensis	126	2.100	[48]
Ichthyosaurus anningae	390	2.591	[50]
Ichthyosaurus breviceps	240	2.380	[51]
Ichthyosaurus communis	256	2.408	[51]
Ichthyosaurus conybeari	216	2.334	[51]
Ichthyosaurus larkini	355	2.550	[52]
Ichthyosaurus somersetensis	438	2.641	[52]
Leptonectes moorei	328	2.516	[57]
Leptonectes solei	1585	3.200	[58]
Leptonectes tenuirostris	523	2.719	[49, 59]
Macgowania janiceps	505	2.703	[60]
Mixosaurus cornalianus	195	2.290	[49, 64]
Mixosaurus kuhnschneyderi	160	2.204	[65]
Mixosaurus xindianensis	223	2.348	[66]
Nannopterygius enthekiodon	600	2.778	[15]
Ophthalmosaurus icenicus	965	2.985	[70]
Ophthalmosaurus natans	1082	3.034	[71]
Palvennia hoybergeti	860	2.934	[28]
Parvinatator wapitiensis	120	2.079	[74]
Phalarodon callawayi	300	2.477	[77]
Phalarodon fraasi	205	2.312	[120, 121]
Phantomosaurus neubigi	550	2.740	[80]
Platypterygius americanus	1250	3.097	[81]
Platypterygius australis	1430	3.155	[82, 83]
Platypterygius hercynicus	1040	3.017	[85]
Platypterygius platydactylus	1170	3.068	[86]
Platypterygius sachicarum	870	2.940	[87]
Qianichthyosaurus xingyiensis	270	2.431	[88]

Supplementary table 4 continued

Taxon	Skull length (mm)	$\log_{10}$ (Skull length (mm))	References
Qianichthyosaurus zhoui	240	2.380	[89]
Sclerocormus parviceps	100	2.000	[91]
Shastasaurus liangae	750	2.875	[93]
Shastasaurus sikkaniensis	3000	3.477	[94]
Shonisaurus popularis	2750	3.439	[95]
Stenopterygius quadriscissus	625	2.796	[43, 98]
Stenopterygius triscissus	634	2.802	[43, 98]
Stenopterygius uniter	537	2.730	[43, 98]
Suevoleviathan disinteger	860	2.934	[99, 100]
Suevoleviathan integer	690	2.839	[99, 100]
Sveltonectes insolitus	570	2.756	[101]
Temnodontosaurus azerguensis	1700	3.230	[102]
Temnodontosaurus eurycephalus	1020	3.009	[104]
Temnodontosaurus platyodon	1790	3.253	[104]
Temnodontosaurus trigonodon	1090	3.037	[99, 107]
Thalattoarchon saurophagis	1200	3.079	[109]
Utatsusaurus hataii	215	2.332	[114]
Wimanius odontopalatus	250	2.398	[116, 122]
Xinminosaurus catactes	290	2.462	[117]

Supplementary code

Code 1 R code implementing the disparity, principal coordinates, diversity, and discrete character rates analyses. This set of five scripts contains the code used to run the main discrete character analyses in R. Outputs include time-scaled trees, discrete rates of evolution, stratigraphic congruence values; PDF files of all figures produced; CSV files of root ages from the time-scaled trees, stratigraphic congruence tests, and statistical tests (pairwise PERMANOVA between epochs for PCA data and pairwise *t*-tests of per-bin disparity).

Code 2 Continuous rates analyses in BayesTraits and plotting in R. Rates analyses were run individually on 100 time-scaled trees then combined into consensus trees with branch rates averaged across all runs. Also includes code to create the traitgram of Fig. 4.

Supplementary methods

Comparison of time-scaling methods To assess the effects of variation in the timing of ichthyosaur evolution on discrete evolutionary rates, we further used the minimum branch length (MBL) tree-scaling method[123,124]. This scales the tree according to occurrence dates, but ensures that each branch length is greater than a given value, rescaling ancestral branches as necessary to ensure this minimum length. Here, we used a MBL of 1 Ma as a reasonable minimum between speciation events and to avoid forcing excessive branch lengths where speciation may occur rapidly. We used the same sample of 120 phylogenetic trees as the main analysis from the Bayesian phylogenetic posterior distribution of Moon [1]. Trees were time-scaled in R[125] using the function `timePaleoPhy` in the package paleotree[123] with point ages sampled from a uniform distribution between their first and last occurrences. Each tree was resampled 10 times to account for the occurrence ranges for each taxon (100 tree topologies × 10 samples = 1000

time-scaled trees total). These MBL time-scaled trees were then used for a further set of discrete character evolutionary rates analyses using function `DiscreteCharacterRate` of R package Claddis[126]. The results of this were used to produce 'spaghetti' plots for epoch-length bins and equal-length bins using modified scripts from Close *et al.* [127]. Code for all these analyses is included in Supplementary Code 1.

Additional disparity metrics Our main results present ichthyosauriform disparity using per-bin pairwise differences between taxa from a distance matrix calculated using maximum observed rescaled distances[126]. Additionally, we compared different distance conversion and disparity metrics.

Claddis provides four distance metrics for discrete character data[126]: raw Euclidean distances (RAW), generalized Euclidean distances (GED)[128], Gower's coefficient (GOW)[129], and maximum observable rescaled distances (MAX)[126]. All four distance metrics were run through the same disparity work flow. Recent studies have shown that GED as implemented in Claddis is susceptible to the completeness of the original data matrix, which may have a strong effect on the resulting disparity[130,131]; therefore we prefer MAX.

Similarly, several different disparity metrics have been developed, each with varying properties. Our main results present mean and weighted mean pairwise distances on MAX as this comes directly from the original data matrix, but we also calculated the pairwise distances for RAW, GED, and GOW distances matrices (Supplementary Figure 1). We ordinated the data using Principal Coordinates Analysis (PCA), both with and without applying a correction to negative eigenvalues[132] and compared the correlation of the PCA data with the original distance matrix.

From the PCA data we used all the resultant axes to calculate per-bin sum of variances, sum of ranges, and centroid distances. These metrics have been used extensively in previous analyses[130,133,134], so we considered it pertinent to compare them. Binning, bootstrap resampling with 500 replicates, and complete rarefaction were completed using the functions `custom.subsets` and `boot.matrix`, and disparity calculations used the function `dispRity`, all from package dispRity[135] in R. Code for this is included in Supplementary Code 1.

Supplementary results

Pairwise disparity Broadly speaking, trends in disparity across all four distance matrices are similar: disparity peaks in the Late Triassic then declines through the Jurassic and Cretaceous (Supplementary Figure 1). The bins that preserve the most completely coded taxa (Supplementary Figure 1 CHAR: Early Jurassic; 201.3 Ma to 171.3 Ma) also show relatively increased disparity in RAW and GED distance matrices compared to GOW and MAX. Indeed, the earliest Jurassic bins are the most disparate for the RAW distance metric with both binning schemes, and for GED the earliest Jurassic bins have relatively higher disparity than GOW and MAX distance matrices. This is most likely a further effect of incompleteness degrading the disparity signal by averaging the difference between taxa[130,131], therefore we prefer the results given by GOW and MAX distance matrices. Rarefying the data shows that maximum disparity is reach quickly with minimal taxa included, and supports using the full taxon sample for each bin (Supplementary Figure 2).

Correlation of ordinated data Negative eigenvalue correction notably decreased the variance described by the first few principal coordinate axes Supplementary Figure 3. The highest correlations between the original and ordinated data were found when including all ordinated axes (Supplementary Figure 3). Without negative eigenvalue correction RAW and GED had the highest correlation, whereas GOW and MAX were reduced to ~0.8. With negative eigenvalue

correction the pattern of correlations with increasing number of axes was more complex: RAW gradually increased whereas GED strongly decreased, but both rapidly increased to 1.0 with the last axes; GOW and MAX correlations both immediately decreased, increased to a peak at ~axis 60, then rapidly increased again when including the last axes.

Disparity of ordinated data Wills [133] asserted that variance based disparity metrics are more suited to measuring overall dissimilarity whereas range-based metrics are appropriate for disparity as they are affected by occurrence and thus show the diversification of morphology. In this context, our results support our conclusions that ichthyosaurs represent an early burst of evolution: both of these metrics show initial high disparity from all distance matrices (Supplementary Figure 4). Sum of variances also has a marked increase between the Early to Middle Triassic and a substantial decline in disparity between the Late Triassic–Early Jurassic in the combination of GOW/MAX distance matrix and uncorrected PCO; otherwise all curves follow similar trends. Sum of variances proves more resilient to sample size in rarefaction than either sum of ranges or centroid distance (Supplementary Figure 5).

All sum of ranges curves display the same trends in disparity, differing only in the magnitude. Similarly, we find early high disparity and an increase between the Early–Middle Triassic (Supplementary Figure 4). Disparity decreases substantially through the later Triassic, but broadly recovers in the Early Jurassic before more log-term decline through to the extinction of the ichthyosaurs. Particularly low disparity (e.g. Middle Jurassic; 171.3 Ma to 161.3 Ma) are those bins represented by few taxa and relative incompleteness.

In the case of centroid distance, although this has been shown to be especially susceptible to issues of 'centroid slippage'[130,131], our results show the same trends as for sum of variances: high early disparity that is sustained through to the Late Jurassic/Early Cretaceous before decline, with dips that are most likely related to incompleteness of specimens (Supplementary Figure 4).

Morphospace occupation of ordinated data Morphospace occupation between Triassic and post-Triassic Ichthyosauriformes is separated in almost all cases (Supplementary Figure 6; except RAW and GED distances). Late Triassic taxa are also separated from earlier Triassic taxa in GOW and MAX distance without negative eigenvalue correction, and are consistently positioned more closely towards the Early Jurassic taxa. The variation in Jurassic and Cretaceous taxa is markedly increased in RAW and GED distances relative to GOW and MAX. Differences within Jurassic and Cretaceous taxa are more represented in PCo axis 2 than axis 3 in the RAW and GED morphospace plots, but in a combination of PCO axes 1 and 3 in GOW and MAX. All RAW and GED morphospace plots show more points towards the origins of the plots than GOW and MAX, a results of 'centroid slippage'[130,131]; in particular these represent the least complete taxa.

Time-scaling and rates Using the MBL time-scaling method created trees with a root age of 253.8 Ma to 268.5 Ma; older than the corresponding root ages from the Hedman scaling method. Rates of discrete character evolution are relatively lower for during the Early–Middle Triassic, but these earlier bins nonetheless show significantly higher rates of evolution that subsequent bins (Supplementary Figure 7). Trends across the whole of ichthyosaur evolution remain similar, although there are increased peaks in the later Early Jurassic and the Late Cretaceous bins. Significantly low rates of discrete character evolution are reached in the Early Jurassic (epoch bins) or Late Triassic (10 Ma bins).

References

1. Moon, B. C. A New Phylogeny of Ichthyosaurs (Reptilia: Diapsida). *J. Syst. Palaeontol.* **17,** 129–155 (2018).
2. Gradstein, F. M., Ogg, J. G., Schmitz, M. & Ogg, G. *The Geologic Time Scale 2012* 1143 pp. (Elsevier, 2012).
3. Reisz, R. R. A Diapsid Reptile from the Pennsylvanian of Kansas. *Special Publ. Mus. Nat. Hist. Univ. Kansas* **7,** 74 pp (1981).
4. Falcon-Lang, H. J., Benton, M. J. & Stimson, M. Ecology of the Earliest Reptiles Inferred from Basal Pennsylvanian Trackways. *J. Geol. Soc. Lond.* **164,** 1113–1118 (2007).
5. Ketchum, H. F. & Barrett, P. M. New Reptile Material from the Lower Triassic of Madagascar: Implications for the Permian–Triassic Extinction Event. *Can. J. Earth Sci.* **41,** 1–8 (2004).
6. Reisz, R. R., Modesto, S. P. & Scott, D. M. A New Early Permian Reptile and Its Significance in Early Diapsid Evolution. *Proc. Royal Soc. B: Biol. Sci.* **278,** 3731–3737 (2011).
7. Ruta, M., Cisneros, J. C., Liebrecht, T., Tsuji, L. A. & Müller, J. Amniotes through Major Biological Crises: Faunal Turnover among Parareptiles and the End-Permian Mass Extinction. *Palaeontol.* **54,** 1117–1137 (2011).
8. Kissel, R. A., Dilkes, D. W. & Reisz, R. R. *Captorhinus magnus* a New Captorhinid (Amniota: Eureptilia) from the Lower Permian of Oklahoma, with New Evidence on the Homology of the Astragalus. *Can. J. Earth Sci.* **39,** 1363–1372 (2002).
9. Fischer, V. *et al.* New Ophthalmosaurid Ichthyosaurs from the European Lower Cretaceous Demonstrate Extensive Ichthyosaur Survival across the Jurassic–Cretaceous Boundary. *PLoS ONE* **7,** e29234–2 (2012).
10. Bardet, N. & Fernández, M. S. A New Ichthyosaur from the Upper Jurassic Lithographic Limestones of Bavaria. *J. Paleontol.* **74,** 503–511 (2000).
11. Maxwell, E. E. Generic Reassignment of an Ichthyosaur from the Queen Elizabeth Islands, Northwest Territories, Canada. *J. Vertebrate Paleontol.* **30,** 403–415 (2010).
12. Druckenmiller, P. S. & Maxwell, E. E. A New Lower Cretaceous (Lower Albian) Ichthyosaur Genus from the Clearwater Formation, Alberta, Canada. *Can. J. Earth Sci.* **47,** 1037–1053 (2010).
13. Jiang, D., Schmitz, L., Hao, W. & Sun, Y.-L. A New Mixosaurid Ichthyosaur from the Middle Triassic of China. *J. Vertebrate Paleontol.* **26,** 60–69 (2006).
14. Dal Sasso, C. & Pinna, G. *Besanosaurus leptorhynchus* n. gen. n. sp. a New Shastasaurid Ichthyosaur from the Middle Triassic of Besano (Lombardy, N. Italy). *Paleontol. Lombarda* **4,** 3–23 (1996).
15. Moon, B. C. & Kirton, A. M. Ichthyosaurs of the British Middle and Upper Jurassic. Part 2, *Brachypterygius, Nannopterygius, Macropterygius* and *Taxa Invalida*. *Monogr. Palaeontogr. Soc.* **172,** 85–176 (2018).
16. Efimov, V. M. The Ichthyosaur *Otschevia pseudoscythica* gen. et sp. nov. from the Upper Jurassic of Ulyanovsk Volga. *Paleontol. Zhurnal* **1998,** 82–86 (1998).
17. Merriam, J. C. Triassic Ichthyopterygia from California and Nevada. *Univ. California, Bull. Dep. Geol.* **3,** 63–108 (1902).

18. Merriam, J. C. Triassic Ichthyosauria, with Special Reference to the American Forms. *Memoirs Univ. California* **1,** 1–252 (1908).

19. McGowan, C. A New Species of *Shastasaurus* (Reptilia: Ichthyosauria) from the Triassic of British Columbia: The Most Complete Exemplar of the Genus. *J. Vertebrate Paleontol.* **14,** 168–179 (1994).

20. Motani, R. *et al.* A Basal Ichthyosauriform with a Short Snout from the Lower Triassic of China. *Nat.* **517,** 485–488 (2015).

21. Fernández, M. S. A New Ichthyosaur from the Tithonian (Late Jurassic) of the Neuquén Basin, Northwestern Patagonia, Argentina. *J. Paleontol.* **71,** 479–484 (1997).

22. Fernández, M. S. A New Long-Snouted Ichthyosaur from the Early Bajocian of Neuquén Basin (Argentina). *Ameghiniana* **31,** 291–297 (1994).

23. Motani, R., Jiang, D., Tintori, A., Rieppel, O. C. & Chen, G. Terrestrial Origin of Viviparity in Mesozoic Marine Reptiles Indicated by Early Triassic Embryonic Fossils. *PLoS ONE* **9,** e88640 (2014).

24. Motani, R. *et al.* Status of *Chaohusaurus chaoxianensis* (Chen, 1985). *J. Vertebrate Paleontol.* **35,** e892011 (2015).

25. Chen, X., Sander, P. M., Cheng, L. & Wang, X. A New Triassic Primitive Ichthyosaur from Yuanan, South China. *Acta Geol. Sinica* **87,** 672–677 (2013).

26. Liu, J. *et al.* The First Specimen of the Middle Triassic Ichthyosaur *Phalarodon atavus* (Ichthyosauria: Mixosauridae) from South China, Showing Postcranial Anatomy and Peri-Tethyan Distribution. *Palaeontol.* **56,** 849–866 (2013).

27. Tyborowski, D. A New Ophthalmosaurid Ichthyosaur Species from the Late Jurassic of Owadów-Brzezinki Quarry, Poland. *Acta Palaeontol. Polonica* **61,** 791–803 (2016).

28. Druckenmiller, P. S., Hurum, J. H., Knutsen, E. M. & Nakrem, H. A. Two New Ophthalmosaurids (Reptilia: Ichthyosauria) from the Agardhfjellet Formation (Upper Jurassic: Volgian/Tithonian), Svalbard, Norway. *Nor. J. Geol.* **92,** 311–339 (2012).

29. Sander, P. M. The Large Ichthyosaur *Cymbospondylus buchseri*, sp. nov. from the Middle Triassic of Monte San Giorgio (Switzerland), with a Survey of the Genus in Europe. *J. Vertebrate Paleontol.* **9,** 163–173 (1989).

30. Fröbisch, N. B., Sander, P. M. & Rieppel, O. C. A New Species of *Cymbospondylus* (Diapsida, Ichthyosauria) from the Middle Triassic of Nevada and a Re-Evaluation of the Skull Osteology of the Genus. *Zool. J. Linnean Soc.* **147,** 515–538 (2006).

31. Brusatte, S. L. *et al.* Ichthyosaurs from the Jurassic of Skye, Scotland. *Scott. J. Geol.* **51,** 1–13 (2015).

32. Von Huene, F. F. Ein neuer Fund von *Eurhinosaurus longirostris*. *Neues Jahrbuch für Geol. und Paläontologie – Abhandlungen* **93,** 277–284 (1951).

33. McGowan, C. A Putative Ancestor for the Swordfish-like Ichthyosaur *Eurhinosaurus*. *Nat.* **322,** 454–456 (1986).

34. McGowan, C. A New Specimen of *Excalibosaurus* from the English Lower Jurassic. *J. Vertebrate Paleontol.* **23,** 950–956 (2003).

35. Paparella, I., Maxwell, E. E., Cipriani, A., Roncacè, S. & Caldwell, M. W. The First Ophthalmosaurid Ichthyosaur from the Upper Jurassic of the Umbrian–Marchean Apennines (Marche, Central Italy). *Geol. Mag.* **154,** 837–858 (2016).

36. Arkhangelsky, M. S. On a New Ichthyosaur of the Genus *Otschevia* from the Volgian Stage of the Volga Region near Ulyanovsk. *Paleontol. J.* **35,** 629–635 (2001).
37. Zverkov, N. G., Arkhangelsky, M. S. & Stenshin, I. M. A Review of Russian Upper Jurassic Ichthyosaurs with an Intermedium/Humeral Contact. Reassessing *Grendelius* McGowan, 1976. *Proc. Zool. Inst. RAS* **319,** 558–588 (2015).
38. Motani, R. Skull of *Grippia Longirostris*: No Contradiction with a Diapsid Affinity for the Ichthyopterygia. *Palaeontol.* **43,** 1–14 (2000).
39. Pan, X.-R. *et al.* Discussion on *Guizhouichthyosaurus tangae* Cao and Luo in Yin et al., 2000 (Reptilia, Ichthyosauria) from the Late Triassic of Guanling County, Guizhou. *Acta Sci. Nat. Univ. Pekinensis* **42,** 697–703 (2006).
40. Chen, X., Cheng, L. & Sander, P. M. A New Species of *Callawayia* (Reptilia: Ichthyosauria) from the Late Triassic in Guanling, Guizhou. *Geol. China* **34,** 974–982 (2007).
41. Brinkman, D. B., Xijin, Z. & Nicholls, E. L. A Primitive Ichthyosaur from the Lower Triassic of British Columbia, Canada. *Palaeontol.* **35,** 465–474 (1992).
42. Cuthbertson, R. S., Russell, A. P. & Anderson, J. S. Cranial Morphology and Relationships of a New Grippidian (Ichthyopterygia) from the Vega–Phroso Siltstone Member (Lower Triassic) of British Columbia, Canada. *J. Vertebrate Paleontol.* **33,** 831–847 (2013).
43. Maisch, M. W. Revision der Gattung *Stenopterygius* Jaekel, 1904 emend. von Huene, 1922 (Reptilia: Ichthyosauria) aus dem unteren Jura Westeuropas. *Palaeodiversity* **1,** 227–271 (2008).
44. Caine, H. & Benton, M. J. Ichthyosauria from the Upper Lias of Strawberry Bank, England. *Palaeontol.* **54,** 1069–1093 (2011).
45. Marek, R. D., Moon, B. C., Williams, M. & Benton, M. J. The Skull and Endocranium of a Lower Jurassic Ichthyosaur Based on Digital Reconstructions. *Palaeontol.* **58,** 723–742 (2015).
46. Motani, R., Manabe, M. & Dong, Z. M. The Status of *Himalayasaurus tibetensis* (Ichthyopterygia). *Paludicola* **2,** 174–181 (1999).
47. McGowan, C. A Remarkable Small Ichthyosaur from the Upper Triassic of British Columbia, Representing a New Genus and Species. *Can. J. Earth Sci.* **32,** 292–303 (1995).
48. Carroll, R. L. & Zhi-Ming, D. *Hupehsuchus*, an Enigmatic Aquatic Reptile from the Triassic of China, and the Problem of Establishing Relationships. *Philos. Transactions Royal Soc. B: Biol. Sci.* **331,** 131–153 (1991).
49. McGowan, C. & Motani, R. in *Handbook of Paleoherpetology* (ed Sues, H.-D.) 175 (Verlag Dr. Friedrich Pfeil, Munich, 2003).
50. Lomax, D. R. & Massare, J. A. A New Species of *Ichthyosaurus* from the Lower Jurassic of West Dorset, England, U.K. *J. Vertebrate Paleontol.* **35,** e903260 (2015).
51. McGowan, C. A Revision of the Latipinnate Ichthyosaurs of the Lower Jurassic of England (Reptilia: Ichthyosauria). *Life Sci. Contributions Royal Ontario Mus.* **100,** 1–30 (1974).
52. Lomax, D. R. & Massare, J. A. Two New Species of *Ichthyosaurus* from the Lowermost Jurassic (Hettangian) of Somerset, England. *Pap. Palaeontol.* **3,** 1–20 (2017).
53. Wiman, C. Ichthyosaurier Aus Der Trias Spitzbergens. *Bull. Geol. Inst. Upsala* **10,** 124–148 (1910).

54. Roberts, A. J., Druckenmiller, P. S., Sætre, G.-P. & Hurum, J. H. A New Upper Jurassic Ophthalmosaurid Ichthyosaur from the Slottsmøya Member, Agardhfjellet Formation of Central Spitsbergen. *PLoS ONE* **9,** e103152. pmid: 25084533 (2014).

55. Delsett, L. L., Roberts, A. J., Druckenmiller, P. S. & Hurum, J. H. A New Ophthalmosaurid (Ichthyosauria) from Svalbard, Norway, and Evolution of the Ichthyopterygian Pelvic Girdle. *PLoS ONE* **12,** e0169971–39 (2017).

56. Fischer, V., Arkhangelsky, M. S., Uspensky, G. N., Stenshin, I. M. & Godefroit, P. A New Lower Cretaceous Ichthyosaur from Russia Reveals Skull Shape Conservatism within Ophthalmosaurinae. *Geol. Mag.* **151,** 60–70 (2014).

57. McGowan, C. & Milner, A. C. A New Pliensbachian Ichthyosaur from Dorset, England. *Palaeontol.* **42,** 761–768 (1999).

58. McGowan, C. A New Species of Large, Long-Snouted Ichthyosaur from the English Lower Lias. *Can. J. Earth Sci.* **30,** 1197–1204 (1993).

59. McGowan, C. *Leptopterygius tenuirostris* and Other Long-Snouted Ichthyosaurs from the English Lower Lias. *Palaeontol.* **32,** 409–427 (1989).

60. McGowan, C. A New and Typically Jurassic Ichthyosaur from the Upper Triassic of British Columbia. *Can. J. Earth Sci.* **33,** 24–32 (1996).

61. Maxwell, E. E. & Caldwell, M. W. A New Genus of Ichthyosaur from the Lower Cretaceous of Western Canada. *Palaeontol.* **49,** 1043–1052 (2006).

62. Fischer, V. *et al.* A Basal Thunnosaurian from Iraq Reveals Disparate Phylogenetic Origins for Cretaceous Ichthyosaurs. *Biol. Lett.* **9,** 20130021–20130021. pmid: 23676653 (2013).

63. Maisch, M. W. & Matzke, A. T. *Mikadocephalus gracilirostris* n. gen., n. sp. a New Ichthyosaur from the Grenzbitumenzone (Anisian–Ladinian) of Monte San Giorgio (Switzerland). *Paläontologische Zeitschrift* **71,** 267–289 (1997).

64. Repossi, E. Il mixosauro degli strati triasici di Besano in lombardia. *Atti della Soc. Italiana di Scienza Nat. e del Museo Civ. di Storia Nat. di Milano* **41,** 361–371 (1902).

65. Brinkmann, W. *Sangiorgiosaurus* n. g. – eine neue Mixosaurier-gattung (Mixosauridae, Ichthyosauria) mit Quetschzähnen aus der Grenzbitumenzone (Mitteltrias) des Monte San Giorgio (Schweiz, Kanton Tessin). *Neues Jahrbuch für Geol. und Paläontologie, Abhandlungen* **207,** 125–144 (1998).

66. Chen, X. & Cheng, L. A New Species of *Mixosaurus* (Reptilia: Ichthyosauria) from the Middle Triassic of Pu'an, Guizhou, China. *Acta Palaeontol. Sinica* **49,** 251–260 (2010).

67. Fernández, M. S. & Talevi, M. Ophthalmosaurian (Ichthyosauria) Records from the Aalenian–Bajocian of Patagonia (Argentina): An Overview. *Geol. Mag.* **151,** 49–59 (2014).

68. Fernández, M. S. A New Ichthyosaur from the Los Molles Formation (Early Bajocian), Neuquen Basin, Argentina. *J. Paleontol.* **73,** 677–681 (1999).

69. Maxwell, E. E., Dick, D. G., Padilla, S. & Parra, M. L. A New Ophthalmosaurid Ichthyosaur from the Early Cretaceous of Colombia. *Pap. Palaeontol.* **2,** 59–70 (2016).

70. Moon, B. C. & Kirton, A. M. Ichthyosaurs of the British Middle and Upper Jurassic. Part 1, *Ophthalmosaurus*. *Monogr. Palaeontogr. Soc.* **170,** 1–84 (2016).

71. Gilmore, C. W. Osteology of *Baptanodon* (Marsh). *Memoirs Carnegie Mus.* **2,** 77–129 (1905).

72. Efimov, V. M. Ichthyosaurs of a New Genus *Yasykovia* from the Upper Jurassic Strata of European Russia. *Paleontol. Zhurnal* **1999,** 91–98 (1999).

73. Arkhangelsky, M. S. On a New Genus of Ichthyosaurs from the Lower Volgian Substage of the Saratov, Volga Region. *Paleontol. Zhurnal* **1997,** 87–91 (1997).
74. Nicholls, E. L. & Brinkman, D. B. in *Vertebrate Fossils and the Evolution of Scientific Concepts* (ed Sarjeant, W. A. S.) 521–535 (Gordon and Breach Publishers, London, 1995).
75. Fischer, V. *et al. Simbirskiasaurus* and *Pervushovisaurus* Reassessed: Implications for the Taxonomy and Cranial Osteology of Cretaceous Platypterygiine Ichthyosaurs. *Zool. J. Linnean Soc.* **171,** 822–841 (2014).
76. Fischer, V. Taxonomy of *Platypterygius campylodon* and the Diversity of the Last Ichthyosaurs. *PeerJ* **4,** e2604–21 (2016).
77. Schmitz, L., Sander, P. M., Storrs, G. W. & Rieppel, O. C. New Mixosauridae (Ichthyosauria) from the Middle Triassic of the Augusta Mountains (Nevada, USA) and Their Implications for Mixosaur Taxonomy. *Palaeontogr. Abteilung A: Paläozoologie—Stratigraphie* **270,** 133–162 (2004).
78. Merriam, J. C. The Skull and Dentition of a Primitive Ichthyosaurian from the Middle Triassic. *Univ. California, Bull. Dep. Geol.* **5,** 381–390 (1910).
79. Maisch, M. W. & Matzke, A. T. Observations on Triassic Ichthyosaurs. Part XIV: The Middle Triassic Mixosaurid *Phalarodon Major* (v. Huene, 1916) from Switzerland and a Reconsideration of Mixosaurid Phylogeny. *Neues Jahrbuch für Geol. und Paläontologie, Monatshefte* **2005,** 596–613 (2005).
80. Sander, P. M. in *Ancient Marine Reptiles* 1–27 (Academic Press, San Diego, 1997).
81. Romer, A. S. An Ichthyosaur Skull from the Cretaceous of Wyoming. *Contributions to Geol. Univ. Wyoming* **7,** 27–41 (1968).
82. Wade, M. *Platypterygius australis,* an Australian Cretaceous Ichthyosaur. *Lethaia* **17,** 99–113 (1984).
83. Wade, M. A Review of the Australian Cretaceous Longipinnate Ichthyosaur *Platypterygius,* (Ichthyosauria, Ichthyopterygia). *Memoirs Qld. Mus.* **28,** 115–137 (1990).
84. Fernández, M. S. & Aguirre-Urreta, M. B. Revision of *Platypterygius hauthali* von Huene, 1927 (Ichthyosauria: Ophthalmosauridae) from the Early Cretaceous of Patagonia, Argentina. *J. Vertebrate Paleontol.* **25,** 583–587 (2005).
85. Kolb, C. & Sander, P. M. Redescription of the Ichthyosaur *Platypterygius hercynicus* (Kuhn 1946) from the Lower Cretaceous of Salzgitter (Lower Saxony, Germany). *Palaeontogr. Abteilung A: Paläozoologie—Stratigraphie* **288,** 151–192 (2009).
86. Broili, F. Ichthyosaurierreste aus der Kreide. *Neues Jahrbuch für Mineral. Geol. und Paläontologie. Beilage-Band* **25,** 422–442 (1908).
87. Páramo, M. E. *Platypterygius sachicarum* (Reptilia, Ichthyosauria) Nueva Especie Del Cretácico de Colombia. *Revista Ingeominas* **6,** 1–12 (1997).
88. Yang, P.-F. *et al.* A New Species of *Qianichthyosaurus* (Reptilia: Ichthyosauria) from Xingyi Fauna (Ladinian, Middle Triassic) of Guizhou. *Acta Sci. Nat. Univ. Pekinensis* **49,** 1002–1008 (2013).
89. Li, C. Ichthyosaur from Guizhou, China. *Chin. Sci. Bull.* **44,** 1329–1333 (1999).
90. Maisch, M. W. & Matzke, A. T. Observations on Triassic Ichthyosaurs. Part XII. A New Early Triassic Ichthyosaur Genus from Spitzbergen. *Neues Jahrbuch für Geol. und Paläontologie, Abhandlungen* **229,** 317–338 (2003).

91. Jiang, D. *et al.* A Large Aberrant Stem Ichthyosauriform Indicating Early Rise and Demise of Ichthyosauromorphs in the Wake of the End-Permian Extinction. *Sci. Reports* **6,** 26232–9 (2016).

92. Motani, R., Jiang, D., Tintori, A., Ji, C. & Huang, J.-D. Pre- versus Post-Mass Extinction Divergence of Mesozoic Marine Reptiles Dictated by Time-Scale Dependence of Evolutionary Rates. *Proc. Royal Soc. B: Biol. Sci.* **284,** 20170241 (2017).

93. Yin, G.-z., Zhou, X., Cao, Z., Yu, Y. & Luo, Y. A Preliminary Study on the Early Late Triassic Marine Reptiles from Guanling, Guizhou, China. *Geol. Geochem.* **28,** 1–23 (2000).

94. Nicholls, E. L. & Manabe, M. Giant Ichthyosaurs of the Triassic—a New Species of *Shonisaurus* from the Pardonet Formation (Norian: Late Triassic) of British Columbia. *J. Vertebrate Paleontol.* **24,** 838–849 (2004).

95. Camp, C. L. Large Ichthyosaurs from the Upper Triassic of Nevada. *Palaeontogr. Abteilung A: Paläozoologie—Stratigraphie* **170,** 139–200 (1980).

96. Fischer, V., Bardet, N., Guiomar, M. & Godefroit, P. High Diversity in Cretaceous Ichthyosaurs from Europe Prior to Their Extinction. *PLoS ONE* **9,** e84709 (2014).

97. Maxwell, E. E., Fernández, M. S. & Schoch, R. R. First Diagnostic Marine Reptile Remains from the Aalenian (Middle Jurassic): A New Ichthyosaur from Southwestern Germany. *PLoS ONE* **7,** e41692 (2012).

98. Maxwell, E. E. New Metrics to Differentiate Species of *Stenopterygius* (Reptilia: Ichthyosauria) from the Lower Jurassic of Southwestern Germany. *J. Paleontol.* **86,** 105–115 (2012).

99. Von Huene, F. F. *Die Ichthyosaurier des Lias und Ihre Zusammenhänge* 114 pp. (Verlag von Gebrüder Borntraeger, Berlin, 1922).

100. Maisch, M. W. A New Ichthyosaur Genus from the Posidonia Shale (Lower Toarcian, Jurassic) of Holzmaden, SW-Germany with Comments on the Phylogeny of Post-Triassic Ichthyosaurs. *Neues Jahrbuch für Geol. und Paläontologie, Abhandlungen* **209,** 47–78 (1998).

101. Fischer, V., Masure, E., Arkhangelsky, M. S. & Godefroit, P. A New Barremian (Early Cretaceous) Ichthyosaur from Western Russia. *J. Vertebrate Paleontol.* **31,** 1010–1025 (2011).

102. Martin, J. E., Fischer, V., Vincent, P. & Suan, G. A Longirostrine *Temnodontosaurus* (Ichthyosauria) with Comments on Early Jurassic Ichthyosaur Niche Partitioning and Disparity. *Palaeontol.* **55,** 995–1005 (2012).

103. Melmore, S. A Description of the Type-Specimen of *Ichthyosaurus crassimanus*, Blake (Owen MS.) *Annals Mag. Nat. Hist.* **6,** 615–619 (1930).

104. McGowan, C. A Revision of the Longipinnate Ichthyosaurs of the Lower Jurassic of England, with Descriptions of Two New Species. *Life Sci. Contributions Royal Ontario Mus.* **97,** 1–37 (1974).

105. Von Huene, F. F. Neue Ichthyosaurier aus Württemburg. *Neues Jahrbuch für Mineral. Geol. und Paläontologie, Beilage-Band B* **65,** 305–320 (1931).

106. Maisch, M. W. & Hungerbühler, A. Revision of *Temnodontosaurus nuertingensis* (v. Huene, 1931), a Large Ichthyosaur from the Lower Pliensbachian (Lower Jurassic) of Nürtingen, South Western Germany. *Stuttgarter Beiträge zur Naturkunde, Ser. B (Geologie und Paläontologie)* **248,** 1–11 (1997).

107. Fraas, E. *Die Ichthyosaurier Der Süddeutschen Trias-Und Jura-Ablagerungen* (Verlag der H. Laupp'schen Buchhandlung, Tübingen, 1891).

108. Mazin, J.-M., Suteethorn, V., Buffetaut, E., Jaeger, J.-J. & Elmcke-Ingavat, R. Preliminary Description of *Thaisaurus chonglakmanii* n.g., n.Sp., a New Ichthyopterygian (Reptilia) from the Early Triassic of Thailand. *Comptes rendus de l'Académie des sciences. Série 2, Mécanique, Physique, Chimie, Sci. de l'univers, Sci. de la Terre* **313,** 1207–1212 (1991).

109. Fröbisch, N. B., Fröbisch, J., Sander, P. M., Schmitz, L. & Rieppel, O. C. Macropredatory Ichthyosaur from the Middle Triassic and the Origin of Modern Trophic Networks. *Proc. Natl. Acad. Sci.* **110,** 1393–1397 (2013).

110. Dalla Vecchia, F. M. First Record of the Rare Marine Reptile *Tholodus schmidi* from the Middle Triassic of the Southern Alps. *Rivista Italiana di Paleontol. e Stratigr.* **110,** 479–492 (2004).

111. Merriam, J. C. New Ichthyosauria from the Upper Triassic of California. *Univ. California, Bull. Dep. Geol.* **3,** 249–263 (1903).

112. Efimov, V. M. A New Family of Ichthyosaurs, the Undorosauridae Fam. Nov. from the Volgian Stage of the European Part of Russia. *Paleontol. Zhurnal* **1999,** 174–181 (1999).

113. Arkhangelsky, M. S. & Zverkov, N. G. On a New Ichthyosaur of the Genus *Undorosaurus*. *Proc. Zool. Inst. RAS* **318,** 187–196 (2014).

114. Shikama, T., Kamei, T. & Murata, M. Early Triassic *Ichthyosaurus, Utatsusaurus hataii* Gen. et Sp. Nov., from the Kitakami Massif, Northeast Japan. *Sci. Reports Tohoku Univ. (Geology)* **48,** 77–97 (1978).

115. Lomax, D. R. A New Leptonectid Ichthyosaur from the Lower Jurassic (Hettangian) of Nottinghamshire, England, UK, and the Taxonomic Usefulness of the Ichthyosaurian Coracoid. *J. Syst. Palaeontol.* **15,** 1–15 (2016).

116. Maisch, M. W. & Matzke, A. T. Observations on Triassic Ichthyosaurs. Part II: A New Ichthyosaur with Palatal Teeth from Monte San Giorgio. *Neues Jahrbuch für Geol. und Paläontologie, Monatshefte* **1998,** 26–41 (1998).

117. Jiang, D. *et al.* New Primitive Ichthyosaurian (Reptilia, Diapsida) from the Middle Triassic of Panxian, Guizhou, Southwestern China and Its Position in the Triassic Biotic Recovery. *Prog. Nat. Sci.* **18,** 1315–1319 (2008).

118. Nicholls, E. L. & Manabe, M. A New Genus of Ichthyosaur from the Late Triassic Pardonet Formation of British Columbia: Bridging the Triassic Jurassic Gap. *Can. J. Earth Sci.* **38,** 983–1002 (2001).

119. Maisch, M. W. & Matzke, A. T. The Mixosaurid Ichthyosaur *Contectopalatus* from the Middle Triassic of the German Basin. *Lethaia* **33,** 71–74 (2000).

120. Jiang, D., Schmitz, L., Motani, R., Hao, W. & Sun, Y.-L. The Mixosaurid Ichthyosaur *Phalarodon* Cf. *P. fraasi* from the Middle Triassic of Guizhou Province, China. *J. Paleontol.* **81,** 602–605 (2007).

121. Økland, I. H., Delsett, L. L., Roberts, A. J. & Hurum, J. H. A *Phalarodon Fraasi* (Ichthyosauria: Mixosauridae) from the Middle Triassic of Svalbard. *Nor. J. Geol.* **98,** 267–288 (2018).

122. Maisch, M. W. & Matzke, A. T. Observations on Triassic Ichthyosaurs. Part V. The Skulls of *Mikadocephalus* and *Wimanius* Reconstructed. *Neues Jahrbuch für Geol. und Paläontologie, Monatshefte* **1999,** 345–356 (1999).

123. Bapst, D. W. Paleotree: An R Package for Paleontological and Phylogenetic Analysis of Evolution. *Methods Ecol. Evol.* **3,** 803–807 (2012).

124. Laurin, M. The Evolution of Body Size, Cope's Rule and the Origin of Amniotes. *Syst. Biol.* **53,** 594–622 (2004).

125. R Core Team. *R: A Language and Environment for Statistical Computing* version 3.6.1. Vienna, Austria: R Foundation for Statistical Computing, 2019.

126. Lloyd, G. T. Estimating Morphological Diversity and Tempo with Discrete Character-Taxon Matrices: Implementation, Challenges, Progress, and Future Directions. *Biol. J. Linnean Soc.* **118,** 131–151 (2016).

127. Close, R. A., Friedman, M., Lloyd, G. T. & Benson, R. B. J. Evidence for a Mid-Jurassic Adaptive Radiation in Mammals. *Curr. Biol.* **25,** 2137–2142 (2015).

128. Wills, M. A., Briggs, D. E. G. & Fortey, R. A. Disparity as an Evolutionary Index: A Comparison of Cambrian and Recent Arthropods. *Paleobiology* **20,** 93–130 (1994).

129. Gower, J. C. A General Coefficient of Similarity and Some of Its Properties. *Biom.* **24,** 774–786 (1971).

130. Flannery Sutherland, J. T., Moon, B. C., Stubbs, T. L. & Benton, M. J. Does Exceptional Preservation Distort Our View of Disparity in the Fossil Record? *Proc. Royal Soc. B: Biol. Sci.* **286,** 20190091 (2019).

131. Lehmann, O. E. R., Ezcurra, M. D., Butler, R. J. & Lloyd, G. T. Biases with the Generalized Euclidean Distance Measure in Disparity Analyses with High Levels of Missing Data. *Palaeontol.,* 1–13 (2019).

132. Caillez, F. The Analytical Solution of the Additive Constant Problem. *Psychom.* **48,** 305–308 (1983).

133. Wills, M. A. Crustacean Disparity through the Phanerozoic: Comparing Morphological and Stratigraphic Data. *Biol. J. Linnean Soc.* **65,** 455–500 (1998).

134. Thorne, P. M., Ruta, M. & Benton, M. J. Resetting the Evolution of Marine Reptiles at the Triassic-Jurassic Boundary. *Proc. Natl. Acad. Sci.* **108,** 8339–8344 (2011).

135. Guillerme, T. dispRity: A Modular R Package for Measuring Disparity. *Methods Ecol. Evol.* **9,** 1755–1763 (2018).
